# Integrating and mapping single-cell transcriptomics across the entire gene expression space

**DOI:** 10.1093/bib/bbag204

**Published:** 2026-04-30

**Authors:** Shuzhen Ding, Xintong Zhai, Zhou Yu, Jingsi Ming

**Affiliations:** KLATASDS-MOE, School of Statistics, East China Normal University, 3663 North Zhongshan Road, Shanghai, 200062, China; KLATASDS-MOE, School of Statistics, East China Normal University, 3663 North Zhongshan Road, Shanghai, 200062, China; KLATASDS-MOE, School of Statistics, East China Normal University, 3663 North Zhongshan Road, Shanghai, 200062, China; KLATASDS-MOE, School of Statistics, East China Normal University, 3663 North Zhongshan Road, Shanghai, 200062, China; Academy of Statistics and Interdisciplinary Sciences, East China Normal University, 3663 North Zhongshan Road, Shanghai, 200062, China

**Keywords:** scRNA-seq, data integration and mapping, gene expression denoising, deep learning

## Abstract

The exponential growth of single-cell transcriptomics datasets has made it essential to integrate heterogeneous datasets for constructing large-scale single-cell reference atlases and mapping query datasets onto these references. However, this integration process is significantly hampered by batch effects, which introduce systematic biases and mask the true biological signals. Moreover, most existing integration methods are mainly limited to the latent space of highly variable genes, restricting their capacity to comprehensively correct the entire transcriptomic landscape and potentially overlooking crucial biological information encoded in genes with lower variability. We introduce scGES, a novel deep learning framework designed to effectively correct batch effects across the entire gene expression space, which leverages information from both highly and lowly variable genes. scGES consists of two main models: scGESI for data integration and scGESM for query mapping. Comprehensive analyses of real data demonstrate that scGES outperforms state-of-the-art methods in batch effect correction and biological variation conservation, thereby enhancing downstream analyses and offering broader biological insights by utilizing information from all genes.

## Introduction

Single-cell RNA-sequencing (scRNA-seq) has revolutionized our understanding of cellular heterogeneity and biomedical research [1, 2]. In recent years, the rapid advancement of single-cell technologies has led to a substantial increase in experimental throughput, providing a wealth of data from different samples, platforms, and conditions [3–5]. The construction of large-scale single-cell reference atlases is of great importance as it offers a comprehensive view of cellular states, their distributions, and relationships across various biological conditions [6–8]. By mapping newly acquired query data onto the reference atlas, researchers can promptly assign cell identities, understand the similarities and differences between new and existing cell populations, and discover rare cell types that might have been previously overlooked [9]. However, the presence of batch effects poses a significant challenge to achieving accurate and reliable integration and mapping [10].

Many methods have been developed for the integration and mapping of scRNA-seq data [11–15]. These methods can be divided into two categories based on their primary focus: (i) the low-dimensional embedding space, and (ii) the original gene expression space. Most published studies, such as scVI [16], Seurat [17], Harmony [18], scANVI [19], cFIT [20], INSCT [21], and Symphony [22], belong to the first category. These methods concentrate on dimensionality reduction while correcting batch effects. Although these methods are highly effective for analyzing overall cellular characteristics, they have limitations in gene-level analysis. Specifically, tasks such as differential expression analysis and co-expression network analysis require precise gene expression data, which are often lost or distorted in low-dimensional embedding. The second category aims to harmonize and denoise the gene expression data. A recent benchmark study suggests that correcting batch effects directly in gene expression space is more challenging than in the embedding space [23]. This is mainly because the majority of genes in the genome are lowly variable genes (LVGs), and batch effects in LVGs are typically more pronounced and difficult to correct compared with highly variable genes (HVGs). The expression of HVGs varies considerably across different cell types, facilitating the identification and correction of batch effects. In contrast, the expression fluctuations of LVGs are relatively minor and are easily masked by batch-related noise. Despite their subtle expression changes, LVGs hold important biological significance, which is crucial for understanding cell biology and enhancing the accuracy of downstream analysis. Among the existing methodologies, CarDEC [24] is designed with a batch correction module for its LVGs compartment, enabling a more comprehensive biological analysis that leverages information beyond HVGs. Scanorama [25] is capable of both integration and denoising. However, it applies a uniform correction strategy across the feature space without distinguishing the distinct biological and technical characteristics inherent to HVGs and LVGs.

The proposed scGES method is designed to construct an integration and mapping approach in the entire gene expression space. Accounting for the different characteristics of HVGs and LVGs, scGES effectively incorporates information from both HVGs and LVGs for data alignment and denoising. Through the correction of batch effects in the original gene expression space, scGES can facilitate downstream analyses and offer more profound biological insights. We conducted comparisons of scGES with other state-of-the-art methods, evaluating their performance in integration, mapping, and denoising across multiple scRNA-seq datasets. The consistent results demonstrate that scGES outperforms other competitive methods, highlighting its advantages in batch effect removal, preservation of biological variation, and facilitation of downstream analysis.

## Methods

### Overview of scGES

**Figure 1:**
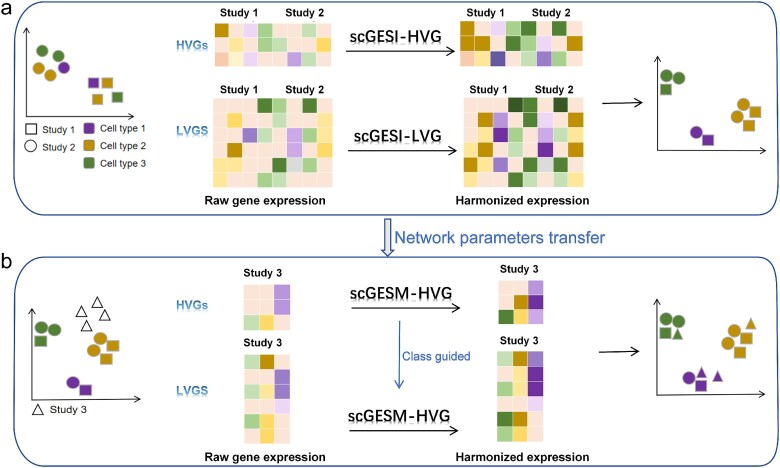
Schematic view of the scGES framework. (a) The scGESI model integrates scRNA-seq data from different batches to construct a reference atlas and obtain harmonized and denoised data in the entire gene expression space. Different modules, scGESI-HVG and scGESI-LVG, are designed for HVGs and LVGs, respectively. (b) The scGESM model projects new data onto the built reference atlas for harmonized and denoised gene expression. Using transfer learning, its two modules (scGESM-HVG and scGESM-LVG) leveraged network parameters learned from scGESI. Guided by scGESM-HVG’s predicted labels, scGESM-LVG aligns query data with the reference atlas for LVGs without label information.

The scGES (Fig. 1) is a unified deep learning framework designed to integrate and map single-cell transcriptomic data across the entire gene expression space. The proposed model consists of two main components: a data integration model, scGESI (Fig. 1a), and a query mapping model, scGESM (Fig. 1b). The scGESI model is intended to construct a reference atlas by integrating heterogeneous scRNA-seq data from different batches. This integration aims to obtain harmonized and denoised data in the entire gene expression space. Given the different characteristics of HVGs and LVGs, scGESI is composed of two modules, scGESI-HVG and scGESI-LVG, which are used to align cells with similar biological states across batches. The scGESM model projects new data onto the constructed reference atlas to obtain harmonized and denoised expression for all genes. Utilizing the idea of transfer learning, the two modules of scGESM (scGESM-HVG and scGESM-LVG) are constructed in a similar manner to those in the scGESI model and leverage the network parameters learned from large reference datasets. Guided by the predicted class labels from scGESM-HVG, scGESM-LVG can align query data with the reference atlas for LVGs without relying on label information. The harmonized gene expression data can facilitate downstream analyses, such as cell annotation, differential expression analysis, and trajectory inference, providing more biological insights as the information from all genes is available.

### Data preprocessing

For each batch, the gene count matrix is denoted as $\widetilde{X}_{\textrm{counts}}^{(b)} \in \mathbb{R}^{N_{b} \times P_{b}}, \quad b = 1, 2, \ldots , B,$ where $N_{b}$ and $P_{b}$ represent the number of cells and genes in batch $b$, respectively. The data are first normalized and log-transformed as follows: 


\begin{align*} & x_{\textrm{norm}, ij}^{(b)} = \log\left(1 + 10\,000 \frac{\tilde{x}_{\textrm{counts}, ij}^{(b)}}{l_{i}^{(b)}}\right) \end{align*}

where $\tilde{x}_{\textrm{counts}, ij}^{(b)}$ represents the gene expression level of the $j$th gene in the $i$th cell of batch $b$, $l_{i}^{(b)}=\tilde{\Sigma }_{j^{\prime}} \tilde{x}_{\textrm{counts}, ij^{\prime}}^{(b)}$ is the library size for the $i$th cell of batch $b$. Subsequently, z-score normalization is applied within each batch using 


\begin{align*} & x_{\textrm{scale}, ij}^{(b)} = \frac{x_{\textrm{norm}, ij}^{(b)}-\mu_{b, j}}{\sigma_{b, j}} \end{align*}

where $\mu _{b, j}$ and $\sigma _{b, j}$ denote the mean and standard deviation of $X_{\textrm{norm}}$ for the $j$th gene in batch $b$, respectively. The data for all the batches are formed as $X = [X^{(1)}_{{\textrm{scale}}}, X^{(2)}_{{\textrm{scale}}},..., X^{(B)}_{{\textrm{scale}}}] \in \mathbb{R}^{N\times P}$ with library size $l=[l^{(1)}, l^{(b)},...,l^{(B)}] \in \mathbb{R}^{N}$, where $N = \sum _{b} N_{b}$, and $P$ is the number of overlapped genes. Next, we identify 2000 HVGs, and the remaining genes that are not selected as HVGs are considered LVGs. The preprocessing steps described above are implemented using the SCANPY package [26].

### The scGESI model

The scGESI model integrates scRNA-seq data using two blocks, which separately consider HVGs and LVGs. HVGs are generally regarded as providing crucial information for distinguishing different cell types among batches, and thus can be used to construct effective anchors for data integration. LVGs can offer additional information for analyzing cell characteristics, but they often exhibit severe batch effects among different datasets. The information from HVGs can assist in eliminating batch effects in LVGs, while LVGs can, in turn, help extract important cell features. Based on these characteristics, we construct the scGESI-HVG and scGESI-LVG blocks (Supplementary Fig. S1).

#### The scGESI-HVG block

The scGESI-HVG block utilizes autoencoders (AE), which consist of an encoder and a decoder. The encoder maps high-dimensional HVG expression data to a low-dimensional latent space, represented as $z_{i,H} = f_{E,H}(x_{i, H}, s_{i}; W_{E, H})$. Here $x_{i,H} \in \mathbb{R}^{P_{H}}$ is scaled-level expression data of HVGs in cell $i$, $P_{H}$ is the number of HVGs, $s_{i}$ is the one-hot batch label vector for cell $i$, $z_{i,H}$ is the low-dimensional embedding of cell $i$ within the scGESI-HVG block, and $W_{E,H}$ represents the corresponding network parameter in the encoder. The decoder maps the distribution from the latent space back to the data space, reconstructing the original data. We assume that the raw counts data $\tilde{x}$ follows a negative binomial distribution with library size $l$, mean $\mu $, and dispersion $\theta $: $NB(\tilde{x};l, \mu , \theta ) = \frac{\Gamma (\tilde{x}+\theta )}{\Gamma (\theta )}\left (\frac{\theta }{\theta +l\mu }\right )^{\theta }\left (\frac{l\mu }{\theta +\tilde{x}}\right )^{\tilde{x}}$. The decoder for scGESI-HVG is denoted as $(\mu _{i,H}, \theta _{i,H}) = f_{D,H}(z_{i,H}, s_{i}; W_{D,H})$, where $\mu _{i,H}$ and $\theta _{i,H}$ are vectors of gene-wise means and dispersions of HVGs in cell $i$, and $W_{D,H}$ is the trainable network parameter in the decoder. When label information is available, we incorporate a classification layer to predict the cell-type label probability, denoted as $q_{i,H} = f_{C,H}(z_{i,H}; W_{C,H})$, where $W_{C,H}$ is the network parameter.

The scGESI-HVG is trained using triplet loss, classification loss, and reconstruction loss (Supplementary Note S1). We define a triplet composed of an anchor cell, a positive cell, and a negative cell. The anchor and positive cells are cells from two different batches but share the same cell-type label or identified based on mutual nearest neighbors (MNNs) (Supplementary Note S1). The negative cell is randomly sampled from the same batch as the anchor cell. The triplet loss ensures that the distance between the anchor and the positive sample is less than the distance between the anchor and the negative sample, and is formulated as: 


\begin{align*} &\mathcal{L}_{\textrm{tri}} = \frac{1}{N_{\textrm{tri}}} \sum^{N_{\textrm{tri}}}_{(a,p,n) \in \mathcal{C}_{\textrm{tri}}} \max\left(\|z_a - z_p\|^2 - \|z_a - z_n\|^2 + \alpha, 0\right),\end{align*}

where $a$, $p$, and $n$ represent the indices of the anchor, positive, and negative cells, respectively, $\mathcal{C}_{\textrm{tri}}$ is the set of identified cell triplets with size $N_{\textrm{tri}}$, and $\alpha $ (default value of 1) is the margin used to enforce the distance between positive and negative pairs.

To quantify the difference between the predicted cell-type label probabilities and the true labels, we define the classification loss as the following cross entropy function: 


\begin{align*} &\mathcal{L}_{\textrm{cls},H} = H(p_c,q_H) = -\sum_i^N p_{i,c} \ln q_{i,H}, \end{align*}

where $p_{i,c}$ is the one-hot true cell-type label vector of cell $i$.

To preserve the original biological information, we define the reconstruction loss using the negative log-likelihood of the negative binomial distribution: 


\begin{align*} & {\mathcal{L}_{\textrm{recon},H} = -\frac{1}{N P_H}\sum_i^N \sum^{P_H}_{j = 1} \ln \left(NB(\tilde{x}_{ij};l_i, \mu_{ij}, \theta_{ij})\right)}. \end{align*}

The total loss for the scGESI-HVG model is the weighted sum of the three losses: 


\begin{align*} & \mathcal{L}_{\mathrm{scGESI-HVG}} = \beta_1 \mathcal{L}_{\textrm{tri}} + \beta_2 \mathcal{L}_{\textrm{cls},H} + \mathcal{L}_{\textrm{recon},H}, \end{align*}

where $\beta _{1}$ and $\beta _{2}$ are hyper-parameters that regulate the proportion between triplet loss, classification loss, and reconstruction loss, with default values of $\beta _{1}=1$ and $\beta _{2} = 5$.

#### The scGESI-LVG block

The scGESI-LVG block also utilizes AE. The encoder in the scGESI-LVG block maps high-dimensional LVG expression to a low-dimensional latent space using $z_{i,L} = f_{E,L}(x_{i, L}, s_{i}; W_{E, L})$. Here $x_{i,L} \in \mathbb{R}^{P_{L}}$ is scaled-level expression data of LVGs in cell $i$, $P_{L}$ is the number of LVGs, $z_{i,L}$ is the low-dimensional embedding of cell $i$ in the scGESI-LVG block, and $W_{E, L}$ is the corresponding network parameter in the encoder. The decoder for scGESI-LVG maps the embeddings of both scGESI-HVG and scGESI-LVG back to the data space using $(\mu _{i,L}, \theta _{i,L}) = f_{D,L}(z_{i,H}, z_{i,L}, s_{i}; W_{D,L})$, where $\mu _{i,L}$ and $\theta _{i,L}$ are vectors of gene-wise means and dispersions of LVGs in cell $i$, and $W_{D,L}$ is the trainable network parameter in the decoder. A classification layer is added to predict the cell-type label probability, denoted as $q_{i,L} = f_{C,L}(z_{i,H}, z_{i,L}; W_{C,L})$, where $W_{C,L}$ is the network parameter.

The classification loss is defined as: 


\begin{align*} &\mathcal{L}_{\textrm{cls},L} = H(p_c,q_L) = -\sum_{i=1}^N p_{i,c} \ln q_{i,L}. \end{align*}

The reconstruction loss of scGESI-LVG is defined by the following loss function: 


\begin{align*} &\mathcal{L}_{\textrm{recon},L} = -\frac{1}{N P_L}\sum_{i=1}^N \sum^{P_L}_{j = 1} \ln \left(NB(\tilde{x}_{ij};l_i, \mu_{ij}, \theta_{ij})\right). \end{align*}

We used an alternating iterative training strategy to optimize the reconstruction loss and the classification loss in separate steps within each training cycle.

### The scGESM model

The scGESM model designates the pre-integrated dataset as the “reference” batch and the new data as the “query” batch. We adopt the concept of transfer learning to construct a mapping structure for the query data by utilizing the weights learned from large reference datasets. Similar to scGESMI, we introduce two blocks, scGESM-HVG and scGESM-LVG (Supplementary Fig. S1), for HVGs and LVGs, respectively. HVGs are used to construct effective anchors for mapping, thereby facilitating the elimination of batch effects in LVGs.

We represent the gene count matrix of the query data as $\tilde{Y} \in \mathbb{R}^{N_{q}\times P_{q}}$, where $N_{q}$ and $P_{q}$ denote the number of cells and genes in the query data, respectively. After following the same preprocessing procedure, we obtain the library size $l^{\prime} \in \mathbb{R}^{N_{q}}$ and scaled data $Y_{\textrm{scale}}\in \mathbb{R}^{N_{q}\times P}$.

#### The scGESM-HVG block

Building upon the scGESI-HVG block, the scGESM-HVG block incorporates an additional batch dimension to represent the query data. The encoder of scGESM-HVG is defined as $z^{\prime}_{i,H} = f^{\prime}_{E,H}(y_{i, H}, s^{\prime}_{i}; W_{E, H}, W^{\prime}_{E, H})$, where $y_{i,H} \in \mathbb{R}^{P_{H}}$ is the scaled expression data of HVGs in cell $i$ of the query data, $s^{\prime}$ is the one-hot batch label vector for cell $i$ in the query data, $z^{\prime}_{i,H}$ is the low-dimensional embedding of cell $i$, and $W^{\prime}_{E,H}$ is the network parameter introduced by the added batch dimension. The decoder for scGESM-HVG is given by $(\mu ^{\prime}_{i,H}, \theta ^{\prime}_{i,H}) = f^{\prime}_{D,H}(z^{\prime}_{i,H}, s^{\prime}_{i}; W_{D,H}, W^{\prime}_{D,H})$, where $W^{\prime}_{D,H}$ is the network parameter introduced by the added batch dimension. The classification layer in scGESI-HVG is directly utilized to predict the cell-type labels for cells in the query data using $q^{\prime}_{i,H} = f_{C,H}(z^{\prime}_{i,H}; W_{C,H})$. Among the parameters of scGESM-HVG, $W_{E,H}$, $W_{D,H}$, and $W_{C,H}$, which are from scGESI-HVG, are fixed and do not participate in training, while $W^{\prime}_{E,H}$ and $W^{\prime}_{D,H}$ are trainable.

The scGESM-HVG is trained using triplet loss and reconstruction loss. A new triplets set $\mathcal{C}^{\prime}_{\textrm{tri}}$ is constructed between the reference and the query data, with cells from the query data serving as the anchor cells (Supplementary Note S1). The loss function of scGESM-HVG is defined as follows: 


\begin{align*} & \mathcal{L}_{\mathrm{scGESM-HVG}} = \beta_{3} \mathcal{L}^{\prime}_{\textrm{tri}} + \mathcal{L}^{\prime}_{\textrm{recon},H}, \end{align*}

where 


\begin{align*} &\mathcal{L}^{\prime}_{\textrm{tri}} = \frac{1}{N^{\prime}_{\textrm{tri}}} \sum_{(a,p,n) \in \mathcal{C}^{\prime}_{\textrm{tri}}} \max\left(\|z^{\prime}_a - z^{\prime}_p\|^2 - \|z^{\prime}_a - z^{\prime}_n\|^2 + \alpha, 0\right),\end{align*}

\begin{align*} &{\mathcal{L}^{\prime}_{\textrm{recon},H} = -\frac{1}{N_q P_H}\sum_{i=1}^{N_q}} \sum^{P_H}_{j = 1} \ln \left(NB(\tilde{y}_{ij};l^{\prime}_i, \mu^{\prime}_{ij}, \theta^{\prime}_{ij})\right),\end{align*}

Here, $\beta _{3}$ regulates the proportion between the triplet loss and the reconstruction loss, with a default value of $\beta _{3}=1$.

#### The scGESM-LVG block

Based on scGESI-LVG, the scGESM-LVG block also adds a batch dimension to represent the query data. The encoder of scGESM-LVG is $z^{\prime}_{i,L} = f^{\prime}_{E,L}(y_{i, L}, s^{\prime}_{i}; W_{E, L}, W^{\prime}_{E, L})$, where $y_{i,L} \in \mathbb{R}^{P_{L}}$ and $z^{\prime}_{i,L}$ are the scaled expression and the low-dimensional embedding of LVGs in cell $i$ in the query data, respectively, and $W^{\prime}_{E, L}$ is the network parameter introduced by the added batch dimension. The decoder for scGESM-LVG is $(\mu ^{\prime}_{i,L}, \theta ^{\prime}_{i,L}) = f^{\prime}_{D,L}(z^{\prime}_{i,H}, z^{\prime}_{i,L}, s^{\prime}_{i}; W_{D,L}, W^{\prime}_{D,L})$, where $W^{\prime}_{D,L}$ is the network parameter introduced by the added batch dimension. The classification layer in scGESI-LVG is directly applied to predict the cell-type labels for cells in the query data using $q^{\prime}_{i,L} = f_{C,L}(z^{\prime}_{i,H}, z^{\prime}_{i,L}; W_{C,L})$. Among the parameters of scGESM-LVG, $W_{E,L}$, $W_{D,L}$ and $W_{C,L}$, which are from scGESI-LVG, are fixed and do not participate in training, while $W^{\prime}_{E,L}$ and $W^{\prime}_{D,L}$ are trainable.

The scGESM-LVG is trained using reconstruction loss and classification loss. The reconstruction loss is 


\begin{align*} &{\mathcal{L}^{\prime}_{\textrm{recon},L} = -\frac{1}{N_q P_L}\sum_{i=1}^{N_q}} \sum^{P_L}_{j = 1} \ln \left(NB(\tilde{y}_{ij};l^{\prime}_i, \mu^{\prime}_{ij}, \theta^{\prime}_{ij})\right).\end{align*}

Unlike the scGESI-LVG model, where cell type labels can be used to guide the classification and remove batch effects in LVGs, the cell type label information is not available for the query data. Therefore, we use the predicted cell type probabilities obtained in scGESM-HVG to guide the classification in scGESM-LVG. The classification loss is defined as 


\begin{align*} &\mathcal{L}^{\prime}_{\textrm{cls},L} = H(q^{\prime}_H,q^{\prime}_L) = -\sum_{i=1}^{N_q} q^{\prime}_{i,H} \ln q^{\prime}_{i,L}.\end{align*}

We used an alternating iterative training strategy to optimize the reconstruction loss and the classification loss in separate steps within each training cycle. Detailed information on the model architecture, training procedures, and parameter configurations has been provided in the Supplementary Note S1.

## Results

### The superiority of scGESI for data integration

To demonstrate the advantages of our method in data integration, we compared scGESI with other integration methods, including CarDEC [24], Harmony [18], scANVI [19], scVI [16], Scanorama [25], cFIT [20], Seurat [17], and INSCT [21] (Supplementary Note S2). Among these methods, scGESI and CarDEC leveraged both HVGs and LVGs, while the rest only utilized HVGs. To illustrate the impact of LVGs, we considered two versions of scGESI and CarDEC: one based on the entire gene set (scGESI and CarDEC) and the other based on the top 2000 HVGs (scGESI-HVG and CarDEC-HVG). Note that, scGESI, scGESI-HVG, scANVI and INSCT incorporated cell-type label information in the integration task. Following recent benchmark studies [23], we conducted a quantitative benchmark evaluation using eleven performance metrics, with five for batch correction and six for biological variation conservation (bio-conservation). The overall score was calculated as a weighted average (40:60) between batch correction and bio-conservation (Supplementary Note S3).

**Figure 2:**
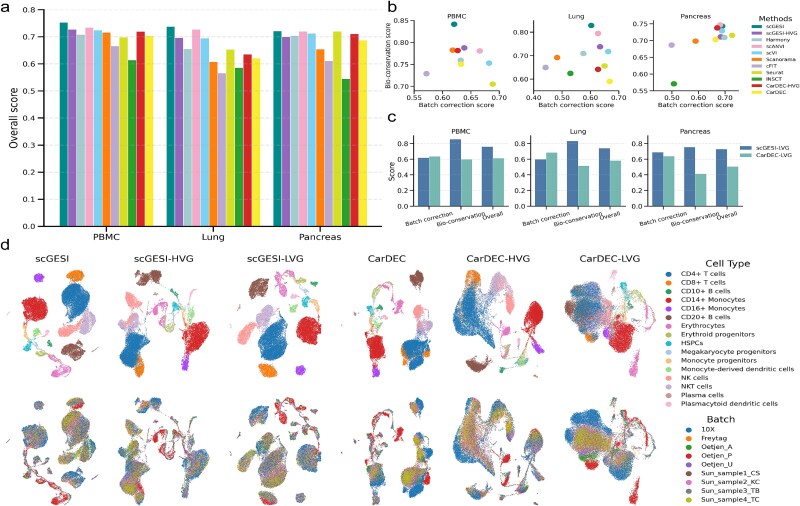
Performance of scGESI and benchmarking methods for data integration. (a) Overall scores for all the benchmarking methods across three integration tasks. (b) Batch correction scores and bio-conservation scores for all the benchmarking methods across three integration tasks. (c) Comparison of the integration performance between scGESI-LVG and CarDEC-LVG. (d) Visualizations of scGESI and CarDEC using the entire gene set (scGESI and CarDEC), HVGs (scGESI-HVG and CarDEC-HVG), and LVGs (scGESI-LVG and CarDEC-LVG) on the human PBMC data. UMAP plots are colored by cell identity annotations (top) and batch labels (bottom).

We conducted a comprehensive evaluation on three integration tasks: human PBMC data with nine batches, and human lung data with 16 batches, and human pancreas data with nine batches [23]. Given the substantial batch discrepancies across different scRNA-seq platforms, these integration tasks were particularly challenging. When analyzing the raw data, this led to lower batch correction and bio-conservation scores (Supplementary Figs S2–S4). Specifically, HVGs typically contain more information about the biological structure and exhibit fewer batch effects across different batches. This suggests that it is more reasonable to construct anchors among batches based on HVGs for integration. However, severe batch effects were observed for LVGs, indicating that their expression levels may be more vulnerable to technical factors. When analyzing the entire gene set, obvious discrepancies were found among different batches. These phenomena highlight the necessity and rationality of our proposed method.

For all three integration tasks, scGESI demonstrated superior performance in eliminating batch effects across individuals and platforms while effectively preserving the biological variation of cell types, resulting in the highest overall scores (Fig. 2a). In particular, scGESI showed remarkable advantages in bio-conservation, especially in the PBMC and lung tasks (Fig. 2b). In contrast, INSCT and cFIT performed poorly, often leading to over-clustering or the merging of distinct cell populations, thereby distorting the true biological diversity (Supplementary Figs S5–S7). Moreover, CarDEC, INSCT, and Scanorama showed less satisfactory performance in batch effect removal in either the PBMC or the pancreas task (Fig. 2d and Supplementary Fig. S7). Meanwhile, scGESI, which is based on the entire gene set, demonstrated better bio-conservation performance and higher overall scores compared with scGESI-HVG. These results confirm that incorporation of scGESI-LVG can effectively enhance the model’s ability to retain biological information.

Given that scGESI and CarDEC can incorporate information from both HVGs and LVGs, we further investigated their integration performance for HVGs and LVGs separately. At both the HVG and whole-gene level, scGESI outperformed CarDEC in terms of batch correction and biological information retention (Figs 2 and Supplementary Figs S5–S7). Regarding LVGs, scGESI-LVG consistently outperformed CarDEC-LVG across multiple integration tasks, attaining higher bio-conservation scores and overall scores (Fig. 2c). Furthermore, CarDEC-LVG showed a significant overlap of distinct cell types, indicating poor preservation of biological heterogeneity (Fig. 2d and Supplementary Figs S6 and S7). For example, in the human PBMC data, CarDEC-LVG failed to distinguish between various cell types such as CD4+ T cells, CD8+ T cells, CD20+ B cells, NKT cells, and NK cells (Fig. 2d). These findings verifies that scGESI-LVG effectively uses the biological information of the HVG part in its branch architecture, and effectively harmonizes the data of the LVG part, which helps preserve the valuable biological information of LVGs.

### The superiority of scGESM for data mapping

Then we investigate the performance of our method in data mapping. After constructing a reference atlas using the scGESI model, the scGESM model can map query datasets onto the reference atlas. For each of the three applications in the integration part, we designated one batch as the query data and the remaining batches as the reference datasets. For this mapping task, we benchmarked scGES against existing data-mapping methods, including Symphony [22], scArches [9] (scANVI, scVI), Seurat, and cFIT [20]. The quantitative benchmark evaluation is the same as that for the integration tasks when treating the reference and query data as two separate batches.

**Figure 3:**
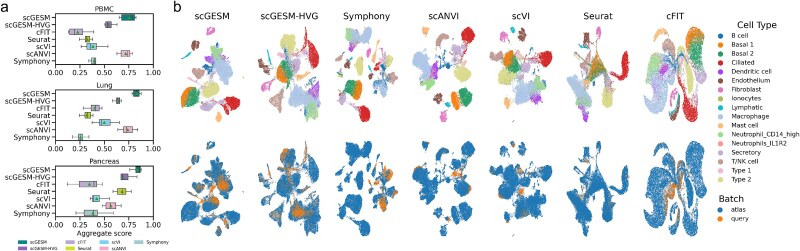
Performance of scGESM and benchmarking methods for data mapping. (a) Overall scores for all the benchmarking methods in the PBMC, lung and pancreas data with each batch designated as the query dataset and the remaining batches as the reference dataset. (b) Visualizations of mapping the B2 batch onto the reference constructed using other batches in human lung data. UMAP plots are colored by cell identity annotations (top) and batch labels (bottom).

The benchmarking results for the three mapping tasks clearly demonstrated that scGESM outperformed other methods in batch correction while excellently preserving biological information, achieving the highest overall scores among all the evaluated methods (Fig. 3a and Supplementary Figs S8–S41). Notably, scGESM outperformed scGESM-HVG, further validating that incorporating LVGs can enhance scGESM’s ability to preserve biological signals. Using the human lung dataset as an example, the results confirmed scGESM’s effectiveness in maintaining crucial cell-type distinctions during integration and successfully harmonizing the reference and query batch (B2) (Fig. 3b). In contrast, Symphony, cFIT, scANVI, and scVI showed unsatisfactory performance in eliminating batch effects for lymphatic and mast cells, resulting in low batch correction scores (Supplementary Fig. S9). In terms of bio-conservation, Seurat failed to clearly distinguish between two types of basal cells, and cFIT incorrectly mapped the query lymphatic cells to the atlas endothelium (Fig. 3b). To assess cell annotation performance, scGESM employs a two-step KNN-based label propagation strategy (Supplementary Note S1), which demonstrated competitive accuracy across multiple datasets (Supplementary Figs S42–S44).

### The superior denoising capability of scGES

One of the primary objectives of scGES is to obtain denoised and harmonized gene expression data for the entire gene set, including both HVGs and LVGs, to enhance the reliability of downstream analysis. Since Scanorama and CarDEC are also able to perform denoising in the entire gene space, we compared their denoising performance with that of scGESI using the data from the three integration tasks. Specifically, we performed PCA analysis on the denoised data obtained from these methods and then conducted UMAP for visualization. Quantitative metrics were calculated based on the PCA embeddings.

**Figure 4:**
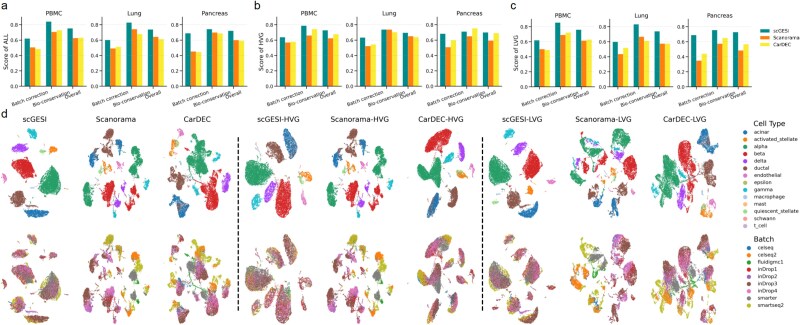
Performance of scGESI, Scanorama, and CarDEC for whole-genome denoising analysis. (a–c) Integration performance metrics on three integration tasks based on denoised gene expression data for the entire gene set (a), HVGs (b), and LVGs (c). (d) Visualizations based on the denoised gene expression data for the entire gene set, HVGs and LVGs for human pancreas data. UMAP plots are colored by cell identity annotations (top) and batch labels (bottom).

The comparative analysis revealed that scGESI outperformed Scanorama and CarDEC in whole-genome space denoising and harmonization, achieving the highest comprehensive scores (Fig. 4a). Moreover, scGESI obtained the highest batch correction scores while effectively preserving fine biological structures. This superior performance was consistently observed for both HVGs and LVGs (Fig. 4b and c). Specifically, in the human pancreas data, scGESI maintained excellent performance across all gene selection conditions and consistently preserved rare cell populations (e.g. schwann and mast cells) (Fig. 4d and Supplementary Fig. S45). In contrast, after data integration using Scanorama and CarDEC, obvious batch effects remained (Fig. 4d). In the case of the lung and pancreas data, Scanorama and CarDEC also exhibited unsatisfactory batch correction performance, as indicated by the presence of many distinct batch-specific clusters that substantially hindered the proper mixing of samples (Supplementary Figs S46 and S47).

### Application to HSC data with pseudotemporal structure

Denoised data can facilitate downstream analyses, such as trajectory analysis with a pseudo-temporal structure. We analyzed a dataset [27, 28] generated through single-cell RNA sequencing, which captured the expression profiles during the differentiation processes of hematopoietic stem cells and progenitor cells. These data consist of 774 cells from SS2 platform and 2401 cells from MARS platform, which exhibit pronounced batch effects (Supplementary Fig. S48). Integrative analysis can help resolve progenitor heterogeneity and test whether the differentiation of common myeloid progenitors (CMP) follows the classical hierarchical commitment towards megakaryocyte erythrocyte progenitors (MEP) and granulocyte macrophage progenitors (GMP) lineages. After integration, scGESI exhibited better performance in both batch correction and biological signal conservation compared with Scanorama and CarDEC (Supplementary Fig. S49). While Scanorama and CarDEC achieved acceptable integration results for HVGs, they failed to fully eliminate batch effects for LVGs. Furthermore, to directly validate the biological nature and robustness of the information captured by the LVG branch, we performed a systematic downsampling analysis. Experiments showed that while the integration performance of scGESI-HVG exhibited a measurable decline as sequencing depth was progressively reduced to 20% of the original data, the performance of scGESI-LVG remained highly stable throughout (Supplementary Fig. S50). This differential robustness indicates that the LVG branch leverages stable biological signals that are resilient to technical fluctuations, rather than dataset-specific technical noise or sampling artifacts. This finding substantiates the core premise that HVGs and LVGs provide complementary biological information for robust single-cell characterization. Additionally, we evaluated the raw and denoised HSC counts using three methods (scGES, CarDEC, and Scanorama) on the following metrics: per-gene expression fidelity, differential expression preservation, and gene–gene covariance structure recovery. scGES consistently outperformed existing methods across these measures (Supplementary Note S5).

**Figure 5:**
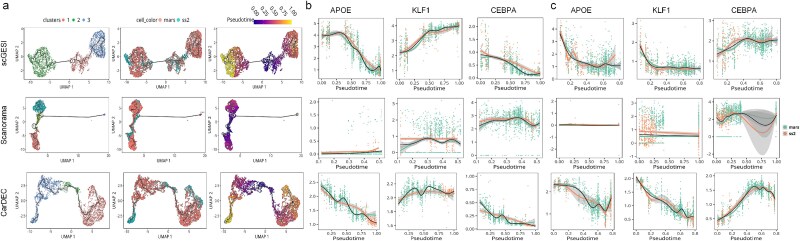
Pseudotime analysis based on the denoised HSC data. (a) Visualizations based on denoised data obtained by scGESI, Scanorama, and CarDEC. UMAP plots are colored by cell identity annotations (left), batch labels (middle), and pseudotime (right). (b and c) The distributions of marker genes KLF1, APOE, and CEBPA in the HVG set against pseudotime from CMP to MEP (b) and GMP (c) based on the denoised gene expression data.

Subsequently, we conducted trajectory analysis on the denoised data using Monocle 3 [29]. The results obtained using scGESI displayed clear pseudo-temporal paths (Fig. 5a). For HVGs, such as APOE, KLF1, and CEBPA, which are specific marker genes for CMP, MEP, and GMP, respectively [27], their expression profiles showed consistent patterns along the pseudotemporal trajectory across batches (Fig 5b and c and Supplementary Fig. S51). For LVGs, we focused on analyzing genes LIMS1, CAR2, and TRF [27]. LIMS1 is a representative gene of CMP; CAR2 can discriminate between MEP and GMP clusters; TRF is a target gene for the genealogy-determining transcription factors CEBPA [28]. Our scGESI results demonstrated that the expression dynamics of these LVGs aligned with the anticipated temporal patterns (Supplementary Figs S52 and S53). In contrast, the denoised expression trends of the marker genes obtained by Scanorama did not show strong variation along the trajectory, suggesting that Scanorama confounded biological signals and blurred the signals of specific genes (Fig 5b and c and Supplementary Figs S51–S53). The pseudotime distributions of marker genes from CarDEC varied dramatically across batches, indicating that CarDEC did not effectively remove batch effects (Fig 5b and c and Supplementary Figs S51–S53). The results for both HVGs and LVGs provided evidence that scGESI effectively restored biological signals by eliminating batch effects along the trajectory, as demonstrated by consistent and aligned pseudotime distributions across batches.

## Discussion

scGES introduces an innovative deep learning framework that addresses critical challenges in single-cell data integration and mapping across the entire gene expression space. Compared with existing state-of-the-art methods for integration, scGESI is capable of correcting batch effects in the complete gene expression space. This approach ensures more comprehensive and accurate data integration, preserves all biological information, and avoids information loss caused by gene selection bias. In mapping tasks, scGESM has established an efficient query mapping process, which enables new single-cell data to be quickly and accurately integrated with compressed reference data, greatly improving analysis efficiency and scalability. After mapping, scGESM directly leverages existing cell state annotations, eliminating the need to re-cluster and re-annotate the integrated data from the beginning. For downstream analysis, scGESI is capable of delivering denoised and harmonized data in the gene expression space, facilitating the acquisition of more comprehensive and in-depth insights. Computational benchmarking further reveals that scGES achieved competitive efficiency on datasets of various scales (Supplementary Note S1 and Figs S54 and S55). As scGES operates on the entire gene set, its memory footprint during training does not inherently possess an advantage over methods that filter genes. Memory usage scales approximately linearly with the total number of input cells.

To assess the contribution of each component in the loss function of scGES, we conducted ablation studies, which demonstrated their effectiveness (Supplementary Note S1 and Fig. S56). Additionally, we conducted systematic sensitivity analyses to validate key parameter choices. The model proved robust over a wide range of loss weights, maintaining stable integration, and mapping performance (Supplementary Figs S57 and S58). Regarding the number of HVGs, our evaluation determined an optimal range of 2000–5000, which effectively balances batch correction and biological signal preservation (Supplementary Fig. S59). The model also demonstrated robustness to incomplete cell-type labels, effectively leveraging both supervised and self-supervised learning modes (Supplementary Fig. S60). When novel cell types are present in the query data, our method mitigates the risk of label-error propagation by utilizing soft labels generated from the HVG branch as the supervisory signal (Supplementary Fig. S61).

We further evaluated scGES under several challenging scenarios using synthetic data generated using scDesign3 [30] (Supplementary Note S4). First, in LVG-only biology tests where discriminatory signals resided exclusively in LVGs, our method successfully recovered the biological separation after integration, confirming its ability to extract meaningful information from LVGs (Supplementary Figs S62 and S63). Second, under varied batch effect structures (shared, independent, or antagonistic effects between HVGs and LVGs) and batch-specific dropout, scGES robustly removed technical artifacts while preserving biological variation (Supplementary Figs S64–S67). Third, in donor–batch confounding tests, results aligned with theoretical identifiability limits: batch effects were corrected when biological and technical variables were not perfectly confounded, highlighting the method’s assumption and practical boundary (Supplementary Figs S68–S70).

**Key Points**

- We propose scGES that can integrate and map scRNA-seq data while correcting batch effects across the entire gene expression space, avoiding information loss and gene selection bias.
- scGES utilizes information from both highly variable and lowly variable genes for alignment and batch correction, improving integration comprehensiveness.
- scGES outputs denoised and harmonized data in the original gene expression space, which is directly suitable for downstream analysis and biological interpretation.
- scGES shows state-of-the-art performance in batch-effect removal and preserving biological signals robustly across various gene selection strategies.

## Supplementary Material

Supplementary_Material_bbag204

## Data Availability

We reprocessed the following public datasets for our tasks: pancreas datasets include GSE81076, GSE85241, GSE86469, GSE84133, GSE81608, and E-MTAB-5061; PBMC of human datasets include GSE120221 and 10X, GSE115189, and GSE128066; The lung datasets were available from GSE130148. Those datasets can be accessed online at https://doi.org/10.6084/m9.figshare.12420968.v7. Additionally, we utilized hematopoietic stem cell datasets from GSE72857 and GSE81682. scGES is available as a python package at https://github.com/szding/scGES. The codes for reproducing the results are available at https://doi.org/10.5281/zenodo.19464476.
