# Supplementary material for: Integrating and mapping single-cell transcriptomics across the entire gene expression space
Source: Brief Bioinform. 2026 Apr 30;27(2):bbag204. doi: 10.1093/bib/bbag204 (PMC13130072; doi:10.1093/bib/bbag204)
Supplement: Supplementary_Material_bbag204 [file supplementary_material_bbag204.pdf]

Supplementary Information for

Integrating and mapping single-cell transcriptomics across the entire gene expression space

Shuzhen Ding[1], Xintong Zhai[1], Zhou Yu[1], Jingsi Ming[1,2*]

[1] *KLATASDS-MOE, School of Statistics, East China Normal University, Shanghai, China*

[2] *Academy of Statistics and Interdisciplinary Sciences, East China Normal University, Shanghai, China*

[*] Corresponding author. Email: jsming@fem.ecnu.edu.cn

This file includes the following subsections:
• Supplementary Notes
• Supplementary Figures

Supplementary Note S1

Triplet neural networks (TNN). Eric Hoffer and Nir Weinberger introduced the concept of TNN in 2015 to train networks to learn a metric space where distances between data points correspond to their semantic similarity [1]. TNN have traditionally been applied in image recognition [2, 3] and more recently for unsupervised dimensionality reduction in scRNA-seq data [4]. Triplet loss compares the similarity between data samples, allowing the model to learn how to map similar samples into closely aligned feature spaces. This boosts discriminative learning and classification efficacy, resulting in a compact yet highly separable latent space. Specifically, a TNN comprises three inputs: an anchor sample, a positive sample, and a negative sample. The network is trained to learn an triplet loss function that maps each sample to a high-dimensional feature space. The objective is to ensure that the distance between the anchor and the positive sample is smaller than the distance between the anchor and the negative sample. Utilizing a batch-aware triplet sampling strategy, scGES enables the scalable and efficient integration of single-cell transcriptomic datasets.

Mutual Nearest Neighbor (MNN). In scRNA-seq, an anchor and a positive sample in a triplet are considered similar cells, while an anchor and a negative sample are considered dissimilar cells. In this article, we construct similar cell pairs in the following situations. Firstly, if cell type information is available, cells with the same type are defined as similar, and cells with different types are defined as dissimilar. Secondly, for batch-specific cells with no common cell types between batches or with only partial cell type information, we supplement anchor-positive pairs by finding the MNN and k-nearest neighbors (KNN) pairs of the anchor cell. Furthermore, for cells not found in the MNN pairs, we calculate the KNN of the remaining cells with all cells to find their positive samples. Here, scGES implements efficient KNN approximation using the

Python-hwnslib package. MNN and KNN calculations are based on the low-dimensional embeddings obtained from the pretrain scGESI model.

Constructing triplets $\mathcal{C}_{tri}$. To overcome batch effects, anchor-positive pairs are typically sampled from different batches. Here we take MNNS between any two batches as anchor-positive pairs. The parameter k defines the number of nearest neighbors and is typically much smaller than the total number of cells in the batch. Therefore, the probability of incorrectly drawing a cell from the KNN is very low, and the randomly sampled cell is likely to be transcriptionally different from the anchor. Random sampling from the same batch thus provides a quick and practical method for defining anchor-negative pairs. The identified set of cell triplets is denoted as $\mathcal{C}_{tri}$.

Constructing mapping triplets $\mathcal{C}'_{tri}$. scGESM need to identify anchor-positive pairs between the query and the reference atlas. Here, cells from the query data serve as anchors, and their corresponding MNNs found in the atlas are designated as positive sample. Moreover, for cells in the query data without an MNN, we compute their KNN among all cells to identify positive samples for the unpaired cells. There are two methods to identify anchor-positive pairs: finding MNNs between the query and each batch in the atlas datasets, or finding MNNs between the query and the entire atlas datasets irrespective of batch distinctions. Our method defaults to the latter approach. Similar to the construction of $\mathcal{C}_{tri}$, negative samples are randomly sampled within the query batch for mapping triplet $\mathcal{C}'_{tri}$.

Loss Function of scGES. In the context of scGES, **triplet loss** accounts for the proximity of transcriptionally similar cells across different batches, helping to overcome batch effects. Through **supervised loss**, the model learns to distinguish between different biological categories, enabling accurate classification and capturing the underlying structure and features of the data to improve data representation quality. By leveraging known cell category labels, scGES efficiently integrates single-cell sequencing data, progressively providing precise label information for previously unlabeled data as correlations among datasets increase. **Reconstruction loss** plays a crucial role in assessing the model's effectiveness in learning data representations. We choose the negative binomial distribution because previous studies have shown that UMI counts are not zero-inflated, and the negative binomial distribution fits the data well [5-7]. By minimizing negative binomial reconstruction loss, the model learns to map input data to output data accurately, achieving effective data reconstruction and denoising. While both triplet loss and clustering loss have the capability to address batch effects, relying solely on triplet loss may lead to excellent batch effect removal at the expense of retaining critical biological information, as observed in the INSCT method. On the other hand, using only clustering loss without incorporating triplet loss may fail to adequately remove batch effects, as seen in the CarDEC method. Therefore, by minimizing a combination of different loss functions, scGES not only enhances low-dimensional embeddings for clustering but also denoises and corrects batch effects in gene features reconstructed from these embeddings, thereby improving the overall quality of gene expression data.

Ablation study. To evaluate the contributions of these components in scGESI, we conducted an ablation study by removing each module individually. In HVG part of scGESI, creating two ablated versions: "scGESI-HVG w/o triplet" (without the triplet loss) and "scGESI-HVG w/o surpervise" (without the surpervise loss). In whole genome space of scGESI, creating three

ablated versions: “scGESI w/o triplet” (without the triplet loss), “scGESI w/o surpervise in HVGs” (without the surpervise loss in HVGs) and “scGESI w/o surpervise in LVGs” (without the surpervise loss in LVGs). To evaluate the contributions of these components in scGESM, we conducted an ablation study by removing each module individually. In HVG part of scGESM, creating one ablated versions: “scGESM-HVG w/o triplet” (without the triplet loss). In whole genome space of scGESM, creating two ablated versions: “scGESM w/o triplet” (without the triplet loss) and “scGESM w/o surpervise in LVGs” (without the surpervise loss in LVGs). Using the PBMC dataset as an example, we illustrate the ablation results of scGESI and scGESM (Supplementary Fig. S54). The full version of scGESI and scGESM achieved the highest overall score, outperforming all ablated versions. These results highlight the critical role of triple loss and supervision guidance in scGESI and scGESM, enabling the model to achieve superior performance in remove batches and retain biometric information.

Label transfer. The scGESM labeling strategy operates through a two-step procedure: reference-based annotation and query propagation. First, for each query cell, we first identify its KNN in the reference dataset. A label is assigned only if over 50% of these neighbors share the same annotation. This ensures high-confidence labeling from reference data. Secound, cells remaining unlabeled after step 1 undergo secondary annotation. Here, we identify their KNN among already-labeled query cells, assigning the most frequent label among these neighbors.

Comparison of label accuracy. For evaluating the accuracy of label transfer, we used MedF1 and ACC indicators to measure the precision of labeling query data (Supplementary Note S1). The scGESM labeling strategy operates through a two-step procedure: reference-based annotation and query propagation (Supplementary Note S3). First, for each query cell, we first identify its k-nearest neighbors (KNN) in the reference dataset. A label is assigned only if over 50% of these neighbors share the same annotation. This ensures high-confidence labeling from reference data. Secound, cells remaining unlabeled after step 1 undergo secondary annotation. Here, we identify their KNN among already-labeled query cells, assigning the most frequent label among these neighbors. In three real datsets analysis, we observed that, compared to other methods, the cell annotation results of scGESM demonstrated competitive performance compared to other mapping methods (Supplementary Figs. S42-S44).

Comparison of computational time and memory usage. To comprehensively analyze the computational costs of mapping task at varying scales, subsets of 100k, 250k, and 500k cells were sampled from the immune cell human-mouse dataset [8]. We evaluated the performance of scGESI and scGESI-HVG against other integration methods. Additionally, we assessed scGESM and scGESM-HVG in comparison to other mapping methods, conducting these analyses on both the data subsets and the complete dataset. cFIT was excluded from the runtime comparison due to termination during large-scale (100k) analysis caused by excessive memory and time constraints. In this computational costs of mapping task design, the MCA_PB_2 batch was designated as the query data, others batch as the reference datasets. The duration required for each method to map the query data onto the reference was meticulously recorded. This study utilized a computing node equipped with a single NVIDIA GeForce RTX 4090 graphics processor with 24,564 MiB (approximately 24 GB) of video memory to compare the memory usage of different methods.

Hyperparameter setting. The base dimensions of our model's autoencoder are [2000, 128, 32, 128, 200]. Set the batch size of all models to 128. For all scGES models, the learning rate is set to $lr = 0.001, iter_{pretrain} = 5$; $iter_{atlas}$=10 or 20; $iter_{map}$= 5. To prevent overfitting in the atlas-LVG and map-LVG model, we employ an iterative training procedure combining semi-supervised loss and reconstruction loss. The default is 1 iterations, where each iteration consists of 1 training sessions for reconstruction loss followed by 1 for semi-supervised loss. For constructing triplets, when labels are available, we randomly select 5 cells of the same type as positives. In the absence of labels, we set the KNN parameter to 20. The hyper-parameters for our loss function are set as follows: $\beta_1 = 1$, $\beta_2 = 5$, $\beta_3 = 1$.

Supplementary Note S2

Benchmarking methods

For evaluation purposes we compared scGESI to existing scRNA-seq integration tools including CarDEC [9], Harmony [10], scANVI [11], scVI [12], Scanorama [13], cFIT [14], Seurat [15] and INSCT [16]. For the mapping task, we benchmarked scGESM against Symphony [17], scArches [18] (scANVI, scVI), Seurat, and cFIT. In our assessment, we employed the default parameters for all methods unless specific parameters were outlined in the original text or tutorial.

CarDEC. CarDEC is a deep learning model that simultaneously corrects batch effects, denoises, and clusters scRNA-seq data. It treats HVGs and LVGs separately, optimizing both embedding and gene expression spaces for improved biological signal recovery. We downloaded the package from https://github.com/jlakkis/CarDEC, and ran CarDEC following its tutorial https://github.com/jlakkis/CarDEC_Codes.

Harmony. Harmony begins with a low-dimensional embedding (for example PCA) and iteratively learns a cell-specific linear correction function until the algorithm converges. In each iteration, it first groups all the cells into different groups based on k-means clustering and then computes the centroids for each cluster and dataset. We install the package through "pip install harmonypy", and ran Harmony function "hm.run_harmony".

scANVI. scANVI is a deep generative model that integrates single-cell RNA-seq datasets by harmonizing data and transferring cell type annotations probabilistically. It combines variational inference with semi-supervised learning to handle labeled and unlabeled cells, enabling robust downstream analyses like differential expression. We downloaded the package from https://github.com/YosefLab/scvi-tools ， and ran scANVI following its tutorial in https://zenodo.org/records/2529945.

scVI. scVI is a variational autoencoder, which takes a count matrix as input and outputs a number of variables including the low-dimensional representation of the latent space. We downloaded the package from https://github.com/YosefLab/scvi-tools，and ran scVI following its tutorial in https://zenodo.org/records/1442920.

Scanorama. Scanorama is based on pairwise analysis in which a list of paired cells from different batches are used to align the datasets into a shared integrated embedding space. In integration task, we used "integrate_scanpy" function from the Scanorama package with default parameters. In denoised task, we used the "correct_scanpy" function from the Scanorama package with default parameters. We install the package through "pip install scanorama".

Seurat. we applied Seurat as in the walk through (https://github.com/satijalab/seurat) with default parameters.

INSCT. INSCT is based on TNNs by sampling triplets in a batch-aware manner. INSCT uses KNNs and MNNs to define similar cell pairs. The k-to-m-ratio parameter determines the number of anchor–positive pairs that are sampled based on KNN relative to MNN pairs; the margin parameter affects the attraction–repulsion theorym which can lead to more or less discrete/continuous embeddings. INSCT as well as all analyses and results presented in the manuscript are available via Github https://github.com/lkmklsmn/insct.

Symphony. Symphony is an efficient algorithm for mapping single-cell query datasets to large, integrated reference atlases. It compresses reference data into portable formats, enabling rapid query localization in a stable low-dimensional embedding. We install the Symphony R package from CRAN, and Jupyter notebooks and scripts to reproduce figures for the analyses in the manuscript are available at https://github.com/immunogenomics/symphony_reproducibility.

scArches. scArches is a deep learning method that uses transfer learning to map new single-cell datasets to existing reference atlases efficiently, preserving biological variation while removing batch effects without sharing raw data. Under this structure, we compare scANVI and scVI. We downloaded the package from https://github.com/theislab/scarches, and ran scArches following its tutorial https://github.com/theislab/scarches-reproducibility.

cFIT. cFIT is a linear model for single-cell RNA-seq data integration and transfer learning. It uses nonnegative matrix factorization to capture shared biological factors while accounting for domain-specific technical effects through location-scale shifts. We downloaded the package from https://github.com/pengminshi/cFIT/ , and ran cFIT following its tutorial https://htmlpreview.github.io/?https://github.com/pengminshi/cFIT/blob/master/vignettes/vignette.html.

Supplementary Note S3

Evaluation metrics

Following recent benchmark studies [8], quantitative benchmark evaluation has been carried out on different methods. An ideal method should remove batch effects while preserving genuine biological variations and efficiently mapping query data to the reference. To assess this, we quantitatively evaluated the integration performance of each method across datasets groups. Batch correction performance was measured using a range of metrics, including the k-nearest neighbor

batch effect test (kBET) [19], principal component regression for batch effects (PCR batch) [19], average silhouette width between batches (batch ASW) [19], graph integral local inverse Simpson index (graph iLISI) [8, 20], and graph connectivity [8]. To evaluate the preservation of biological signals, we calculated biological variation preservation scores using metrics such as the adjusted Rand index (ARI) [21], normalized mutual information (NMI) [22], cell type ASW, graph cell type local inverse Simpson index (graph cLISI) [8, 20], and silhouette scores for label separation [8]. Finally, we calculated an overall performance score as a weighted average of 40:60 between batch correction and biological variation preservation scores. Additionally, in assessing mapping effectiveness, we treated the reference and query data as two separate batches and applied the aforementioned indicators. For evaluating the accuracy of label transfer, we used MedF1 and ACC indicators to measure the precision of labeling query data. Higher values for each indicator reflect better performance.

PCR batch. Principal component regression (PCR) quantifies batch removal by calculating the (R^2) from a linear regression of the batch variable onto each principal component (PC). The variance contribution of the batch effect per PC is determined by the product of the variance explained by each PC and the corresponding (R^2). Summing these contributions across all PCs provides the total variance explained by the batch variable.

BatchASW. To evaluate the output of data integration, we use an improved method (batchASW score) to measure batch mixing. This indicator is calculated on the embedding provided by the integration method, or in the case of feature output, using the PCA of the expression matrix. Overall, a batchASW of 1 indicates ideal batch mixing, while a value of 0 indicates severe batch separation. We use scikit-learn (v.1.5.0) implementation to calculate these scores.

Graph iLISI. The original iLISI metric quantifies dataset mixing by measuring the effective number of datasets in a local neighborhood, with a value of 1 indicating poor mixing and 2 indicating good mixing between two datasets. Its extension, Graph iLISI, adapts this calculation for graph structures. For interpretation, the values are rescaled to the [0, 1] interval by subtracting 1.

Graph connectivity. Graph connectivity assesses whether the graph correctly connects cells of the same cell type labels among batches. We used the Scanpy pipeline to derive a graph representation of integrated cell embeddings. The score ranges from 0 to 1, with 1 indicating perfect connectivity.

kBET. The kBET algorithm employs a Pearson χ^2-based test to evaluate whether the label composition of k nearest neighbors (kNN) aligns with the expected global batch labels. In this study, we used k=50 and computed kNN graphs through Scanpy preprocessing. kBET analyses were conducted on the batch variable for each cell identity, accounting for technical and cell type frequency variations. The minimum and maximum k values were set to 10 and 100, respectively. Results from connected components too small for kBET were assigned a score of 1. Diffusion-based correction was applied to standardize kNN sizes, enabling consistent output comparison.

NMI. NMI measures the overlap between two clusterings and was used to compare cell-type labels with optimized Louvain clusters on integrated data, ranging from 0 (no correlation) to 1 (perfect match). We used the scikit-learn (v.1.5.0) implementation, optimizing Louvain clustering across resolutions from 0.1 to 2 to achieve the highest NMI score.

ARI. The Rand index measures the agreement between two clusterings, considering matches and mismatches, with the Adjusted Rand Index (ARI) correcting for random labeling. We utilized the scikit-learn (v.1.5.0) implementation to compare cell-type labels with NMI-optimized Louvain clustering on integrated data.

Cell type ASW. The cell type ASW score measures the conservation of dataset-specific cell types. we compute the ASW of isolated versus nonisolated labels on the PCA embedding (ASW metric above) and scale this score to be between 0 and 1. The final score for each metric version consists of the mean isolated score of all isolated labels.

Isolated label F1. The isolated label metric to assess data integration methods for cell identity labels present in few batches. This score, based on the F1 metric, optimizes cluster assignments across Louvain resolutions from 0.1 to 2. The F1 score, a weighted mean of precision and recall, ranges from 0 to 1, with 1 indicating perfect capture of isolated label cells.

Isolated label silhouette. The isolated label silhouette metric, while conceptually related to the isolated label F1 score, employs the ASW (Average Silhouette Width) to assess the preservation of dataset-specific cell types. In our experimental setup, the resulting scores were normalized to the [0, 1] interval.

Graph cLISI. The original cLISI measures the effective number of cell types in a neighborhood, where 1 means that the cell population is well preserved and larger values indicate the mixing of different cell populations. Graph cLISI extends cLISI by enabling the calculation on graphs. The values were rescaled to [0, 1], where higher values indicate good performance in preserving biological variation.

Per-cluster mapping metric. The isolated label silhouette metric, while conceptually related to the isolated label F1 score, employs the ASW (Average Silhouette Width) to assess the preservation of dataset-specific cell types. In our experimental setup, the resulting scores were normalized to the [0, 1] interval.

MedF1. We calculated overall accuracy across all query cells and cell type F1-scores (the harmonic mean of precision and recall, ranging from 0 to 1). Specifically defined as: Precision $=\frac{TP}{TP+FP}$, recall $=\frac{TP}{TP+FN}$, $\mathrm{F1} = 2\frac{\mathrm{precision}*\mathrm{recall}}{\mathrm{precision}+\mathrm{recall}}$. The MedF1 of the final report is the median F1 score of all cell types, used to comprehensively reflect the classification consistency of the model on different cell types.

ACC. The definition of ACC is the proportion of correctly predicted labels compared to true labels, with a value range of [0, 1]. The closer to 1, the better the prediction result.

Supplementary Note S4

The simulated dataset is derived from the 10X Genomics human PBMC dataset (https://figshare.com/ndownloader/files/40581965), which was preprocessed using scDesign3 [23]. It consists of two batches, comprising 6,276 cells and 1,000 genes. In our simulation setup, the number of highly variable genes (HVGs) was set to 400, and the number of lowly variable genes (LVGs) was set to 600. The details of the simulated operation are as follows:

First, we aimed to construct a scenario for LVG-only biology where the distinction between two biologically related cell states (Cytotoxic T cells and Natural Killer cells) is absent in the HVG space but present in the LVG space. Using scDesign3, the HVGs were designed to have no mean expression difference between the two states (by setting the cell state effect $\alpha_{jc} = \alpha_{jc'}$ for all HVGs, where c denote Cytotoxic T and c' denote Natural Killer). The LVGs were allowed to carry differential expression signals by keeping the original cell state effects, thereby containing the discriminatory biological information.

Secound, to systematically evaluate the robustness of our method under different batch effect structures, we have followed your recommendation and performed a series of experiments. We considered the following five scenarios by generating synthetic data using scDesign3:

(1) Shared batch effects of HVGs and LVGs: Applied identical batch effects shifts ($\alpha_{jb} \sim N(2, 0.25)$ for j in HVGs and LVGs) to both HVGs and LVGs.

(2) Independent batch effects of HVGs and LVGs: Applied strong batch effects to HVGs ($\alpha_{jb} \sim N(3, 0.25)$ for j in HVGs) and weaker, independent effects to LVGs ($\alpha_{jb} \sim N(1, 0.25)$ for j in LVGs).

(3) Antagonistic batch effects of HVGs and LVGs: Applied positive batch effects to HVGs ($\alpha_{jb} \sim N(2, 0.25)$ for j in HVGs) and negative effects to LVGs ($\alpha_{jb} \sim N(-2, 0.25)$ for j in LVGs).

(4) Batch-specific dropout: Introduced a higher dropout rate in 10x Chromium (v2) to 10%.

Finally, we performed a systematic identifiability stress test using three designed scenarios to clarify the assumptions and limitations of our method when donor identity is confounded with batch.

(1) Complete confounding (1-donor-per-batch): Donor_1 is exclusively in Batch_1, and Donor_2 is exclusively in Batch_2. Here, batch and donor are perfectly aliased.

(2) Partial Confounding: Donor_1 is only in Batch_1, Donor_3 is only in Batch_2, and Donor_2 is split across both batches (e.g., 40% in Batch_1, 40% in Batch_2). This creates a shared biological anchor across batches.

(3) Fully Balanced (No Confounding): All three donors are evenly distributed across both batches. This is the ideal, identifiable scenario.

Supplementary Note S5

We systematically evaluated raw and denoised HSC counts using three methods (scGES, CarDEC, and Scanorama) across multiple dimensions. For gene expression fidelity, we calculated several basic statistical metrics. In differential expression preservation, the F1-score metric is used to compare the consistency between the true and predicted data in differential expression analysis, where logFC calculates the log fold change for each gene between two groups of cells to identify differentially expressed genes. In gene–gene structure preservation, for computational efficiency, we randomly selected 1,000 genes from the LVG set and computed their pairwise correlations, based on which we employed the Louvain clustering algorithm to construct a graph representing gene-gene relationships. The structural consistency of the genes was then evaluated by comparing the graph derived from the predicted data with that from the ground truth data.

Table S1. Metric for the integration output of scGES、CarDEC and Scanorama on the HSC data

	metrics	scGES	CarDEC	Scanorama
Per-gene fidelity	Average Pearson correlation	0.7	0.2	0.02
	Median Pearson correlation	0.73	0.21	0.01
	RMSE on log1p counts	1.26	1.56	1.76
	MAE on log1p counts	0.83	0.9	0.91
	Bias^2/Variance	2.97/2.6	2.94/0.02	3.8/0.0001
Differential expression preservation	Average Spearman (logFC)	0.77	0.73	-0.0004
	Average F1-score	0.91	0.51	0.06
Gene–gene structure preservation	edge_precision	0.41	0.37	0.17
	edge_recall	0.92	0.4	0.0002
	edge_f1	0.57	0.38	0.0004

Supplementary Figures

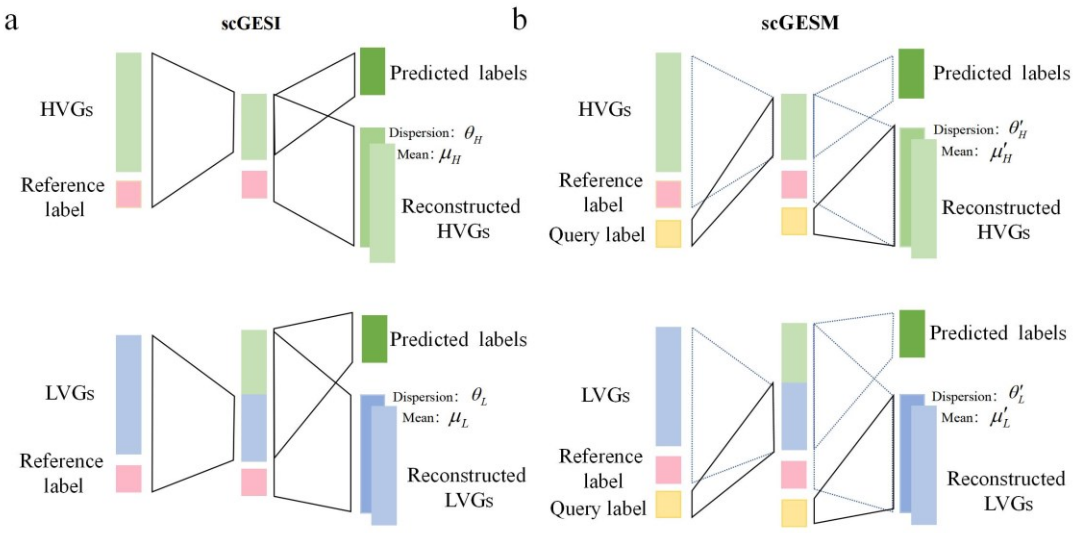

Fig.S1 Schematic diagram of scGES network structure. a, scGESI; The scGESI model aims to construct a reference atlas by integrating scRNA-seq data across the entire gene expression space. According to the different characteristics of HVGs and LVGs, we consider HVGs and LVGs as different feature blocks and use conditional autoencoder models to define them separately. b, scGESM; The scGESM model aims to project new data onto the pre-constructed reference atlas, thereby acquiring harmonized expression for all genes. We utilize the idea of transfer learning to construct a mapping structure for query data by leveraging weights learned from scGESI model. Here, HVGs are used to construct effective anchors for mapping, thus helping eliminate batch effects in LVGs. In the network architecture diagram, the solid line indicates the active training network, while the dashed line represents fixed network weights.

a

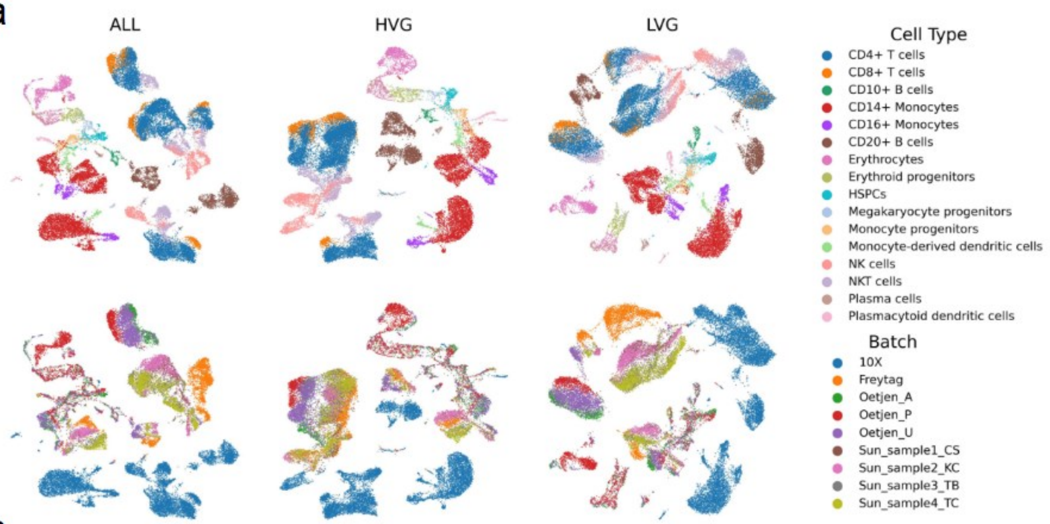

b

Method	Batch correction: PCR batch	Batch correction: Batch ASW	Batch correction: Graph iLISI	Batch correction: Graph connectivity	Batch correction: kBET	Bio-conservation: NMI cluster/label	Bio-conservation: ARI cluster/label	Bio-conservation: Cell type ASW	Bio-conservation: Isolated label F1	Bio-conservation: Isolated label silhouette	Bio-conservation: Graph cLISI	Aggregate score: Batch correction	Aggregate score: Bio-conservation	Aggregate score: Total
HVG	0.39	0.84	0.10	0.97	0.16	0.69	0.44	0.58	0.84	0.67	1.00	0.49	0.70	0.62
ALL	0.15	0.84	0.03	0.95	0.14	0.64	0.31	0.55	0.82	0.64	1.00	0.42	0.66	0.57
LVG	0.00	0.82	0.04	0.95	0.16	0.61	0.27	0.52	0.83	0.61	0.99	0.39	0.64	0.54

Fig.S2 Analysis on the raw human PBMC data. a, Visualizations using all genes, HVGs and LVGs with the Scanpy Louvain workflow. UMAP plots are colored by cell identity annotations (top) and batch labels (bottom). b, Metrics for evaluating the integration performance.

a

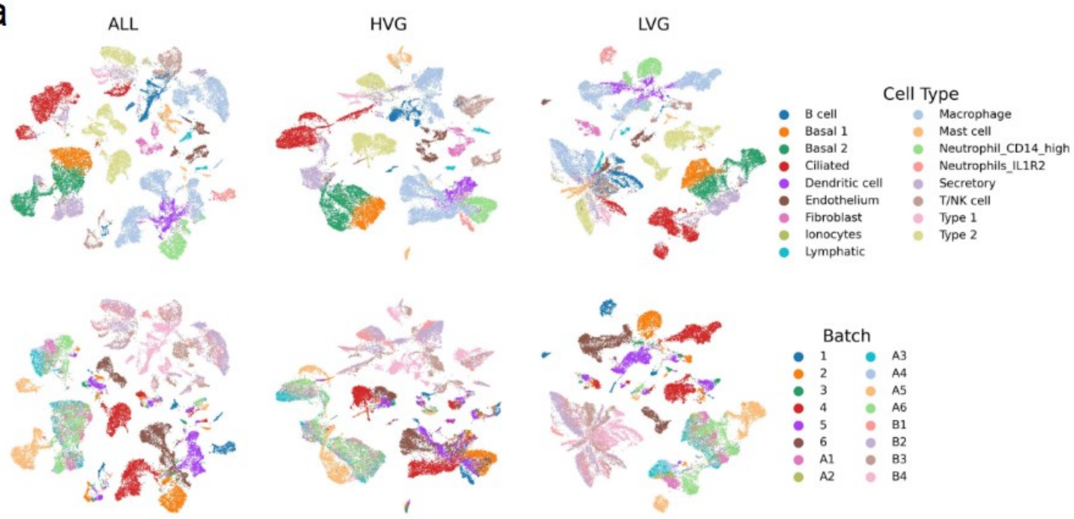

b

Method	Batch correction: PCR batch	Batch correction: Batch ASW	Batch correction: Graph iLISI	Batch correction: Graph connectivity	Batch correction: kBET	Bio-conservation: NMI cluster/label	Bio-conservation: ARI cluster/label	Bio-conservation: Cell type ASW	Bio-conservation: Isolated label F1	Bio-conservation: Isolated label silhouette	Bio-conservation: Graph cLISI	Aggregate score: Batch correction	Aggregate score: Bio-conservation	Aggregate score: Total
HVG	0.22	0.84	0.03	0.95	0.27	0.76	0.52	0.61	0.91	0.72	1.00	0.46	0.75	0.64
ALL	0.00	0.85	0.01	0.92	0.23	0.72	0.39	0.56	0.87	0.68	1.00	0.40	0.70	0.58
LVG	0.00	0.86	0.01	0.90	0.24	0.68	0.35	0.52	0.82	0.63	1.00	0.40	0.67	0.56

Fig.S3 Analysis on the raw human lung data. a, Visualizations using all genes, HVGs and LVGs with the Scanpy Louvain workflow. UMAP plots are colored by cell identity annotations (top) and batch labels (bottom). b, Metrics for evaluating the integration performance.

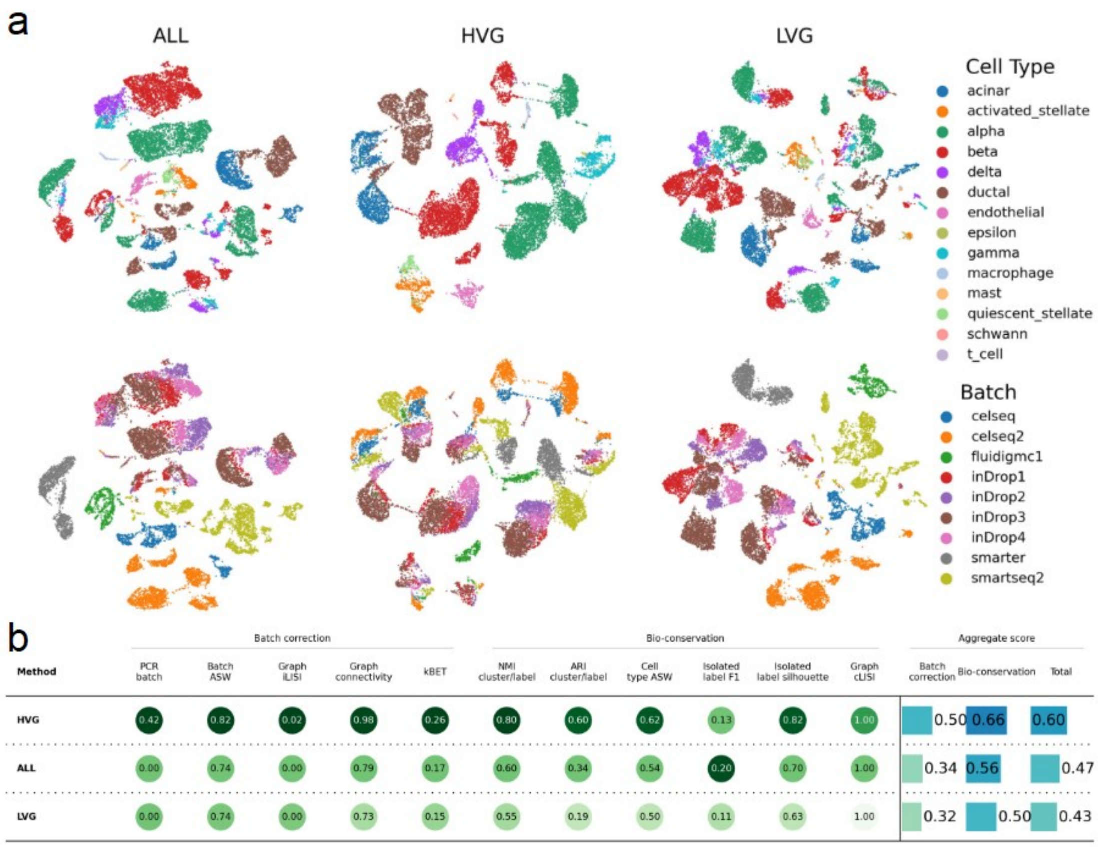

Fig.S4 Analysis on the raw human pancreas data. a, Visualizations using all genes, HVGs and LVGs with the Scanpy Louvain workflow. UMAP plots are colored by cell identity annotations (top) and batch labels (bottom). b, Metrics for evaluating the integration performance.

a

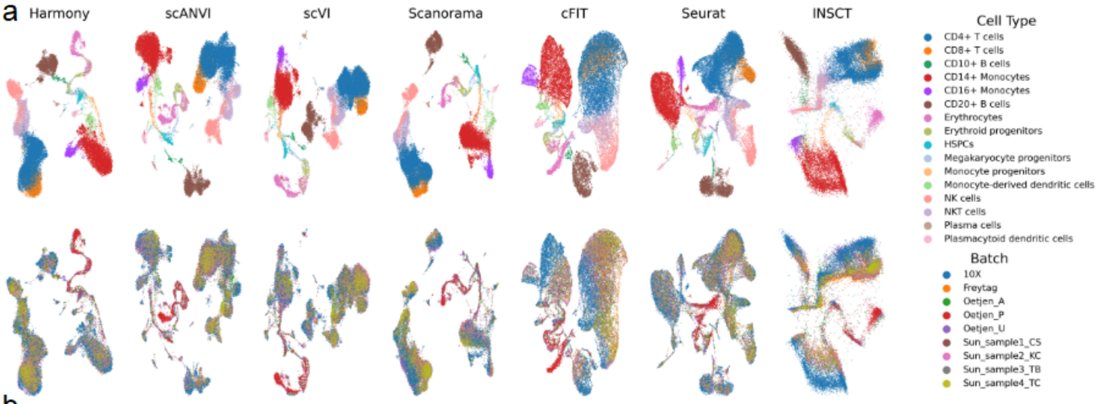

b

Method	Batch correction					Bio-conservation						Aggregate score		
	PCR batch	Batch ASW	Graph iLISI	Graph connectivity	kBET	NMI cluster/label	ARI cluster/label	Cell type ASW	Isolated label F1	Isolated label silhouette	Graph cLISI	Batch correction	Bio-conservation	Total
scGESI	0.80	0.85	0.24	0.98	0.22	0.91	0.93	0.62	0.91	0.67	1.00	0.62	0.84	0.75
scANVI	0.82	0.90	0.33	0.99	0.29	0.81	0.77	0.60	0.87	0.64	1.00	0.67	0.78	0.73
scGESI-HVG	0.89	0.87	0.25	0.98	0.20	0.83	0.85	0.60	0.82	0.63	1.00	0.64	0.79	0.73
scVI	0.89	0.91	0.34	0.99	0.28	0.81	0.77	0.55	0.80	0.60	0.99	0.68	0.75	0.72
CarDEC-HVG	0.90	0.82	0.22	0.97	0.22	0.80	0.82	0.68	0.69	0.70	1.00	0.63	0.78	0.72
Scanorama	0.69	0.93	0.20	0.98	0.28	0.85	0.80	0.58	0.86	0.60	1.00	0.62	0.78	0.72
Harmony	0.67	0.86	0.36	0.98	0.28	0.81	0.77	0.58	0.81	0.58	1.00	0.63	0.76	0.71
CarDEC	0.91	0.91	0.21	0.97	0.17	0.80	0.75	0.57	0.77	0.61	1.00	0.63	0.75	0.70
Seurat	0.88	0.85	0.41	0.97	0.34	0.77	0.73	0.57	0.63	0.53	0.99	0.69	0.71	0.70
cFIT	0.53	0.83	0.27	0.98	0.24	0.76	0.71	0.57	0.76	0.59	0.99	0.57	0.73	0.67
INSCT	0.72	0.73	0.25	0.88	0.33	0.66	0.37	0.55	0.67	0.57	0.99	0.58	0.64	0.61

Fig.S5 Performance of benchmarking methods for data integration on the PBMC data. a, Visualization based on the integration results of benchmarking methods. UMAP plots are colored by cell identity annotations (top) and batch labels (bottom). b, Metrics for evaluating the integration performance for benchmarking methods.

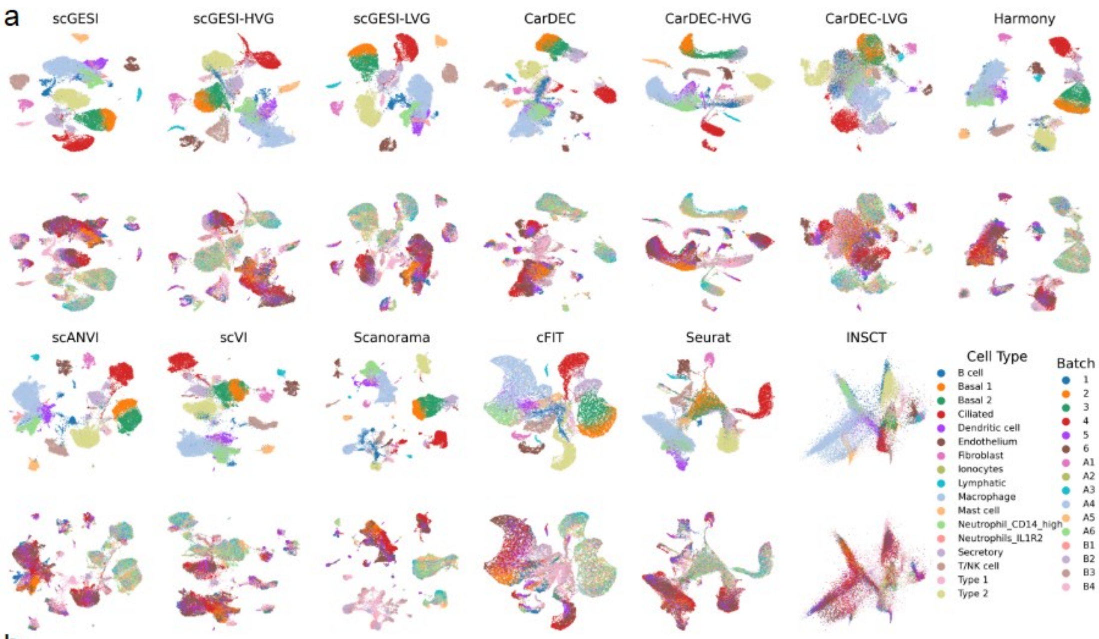

b

Method	Batch correction					Bio-conservation						Aggregate score		
	PCR batch	Batch ASW	Graph iLISI	Graph connectivity	kBET	NMI cluster/label	ARI cluster/label	Cell type ASW	Isolated label F1	Isolated label silhouette	Graph cLISI	Batch correction	Bio-conservation	Total
scGESI	0.67	0.84	0.13	0.99	0.38	0.84	0.76	0.62	0.99	0.76	1.00	0.60	0.83	0.74
scANVI	0.74	0.89	0.13	0.99	0.39	0.81	0.72	0.61	0.89	0.73	1.00	0.63	0.79	0.73
scGESI-HVG	0.84	0.84	0.14	0.96	0.38	0.73	0.57	0.59	0.86	0.69	0.99	0.63	0.74	0.70
scVI	0.88	0.90	0.17	0.97	0.39	0.72	0.56	0.54	0.85	0.64	0.99	0.66	0.72	0.70
Harmony	0.44	0.87	0.18	0.95	0.44	0.73	0.60	0.59	0.76	0.58	0.99	0.58	0.71	0.66
Seurat	0.83	0.80	0.23	0.88	0.52	0.62	0.50	0.52	0.69	0.64	0.96	0.65	0.66	0.65
CarDEC-HVG	0.86	0.84	0.12	0.96	0.36	0.73	0.55	0.62	0.29	0.67	0.99	0.63	0.64	0.64
CarDEC	0.93	0.94	0.13	0.97	0.38	0.71	0.48	0.54	0.26	0.57	0.99	0.67	0.59	0.62
Scanorama	0.17	0.93	0.08	0.92	0.31	0.73	0.49	0.57	0.74	0.63	1.00	0.48	0.69	0.61
INSCT	0.55	0.62	0.20	0.77	0.51	0.60	0.43	0.52	0.61	0.62	0.96	0.53	0.62	0.59
cFIT	0.03	0.75	0.11	0.96	0.35	0.69	0.45	0.53	0.67	0.56	0.99	0.44	0.65	0.57

Fig.S6 Performance of benchmarking methods for data integration on the lung data. a, Visualization based on the integration results of benchmarking methods. UMAP plots are colored by cell identity annotations (top) and batch labels (bottom). b, Metrics for evaluating the integration performance for benchmarking methods.

a

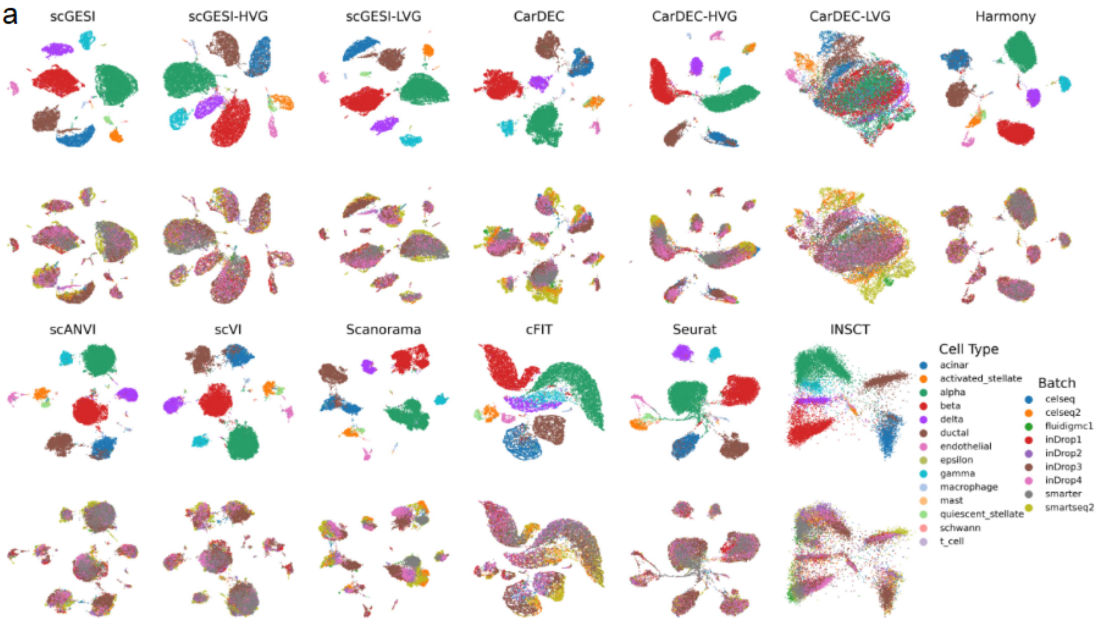

b

	Batch correction					Bio-conservation						Aggregate score		
Method	PCR batch	Batch ASW	Graph iLISI	Graph connectivity	kBET	NMI cluster/label	ARI cluster/label	Cell type ASW	Isolated label F1	Isolated label silhouette	Graph cLISI	Batch correction	Bio-conservation	Total
scGESI	0.86	0.81	0.29	0.99	0.50	0.96	0.98	0.65	0.10	0.76	1.00	0.69	0.74	0.72
scANVI	0.82	0.86	0.29	0.99	0.44	0.92	0.95	0.62	0.18	0.81	1.00	0.68	0.75	0.72
Seurat	0.85	0.86	0.37	0.98	0.57	0.89	0.91	0.69	0.08	0.72	1.00	0.73	0.72	0.72
scVI	0.84	0.87	0.30	0.99	0.44	0.91	0.95	0.59	0.13	0.81	1.00	0.69	0.73	0.71
CarDEC-HVG	0.90	0.85	0.24	0.98	0.38	0.91	0.95	0.78	0.12	0.68	1.00	0.67	0.74	0.71
Harmony	0.73	0.87	0.38	0.98	0.53	0.91	0.95	0.66	0.09	0.64	1.00	0.70	0.71	0.70
scGESI-HVG	0.88	0.77	0.30	0.98	0.49	0.91	0.94	0.73	0.10	0.59	1.00	0.68	0.71	0.70
CarDEC	0.93	0.92	0.21	0.94	0.32	0.91	0.95	0.58	0.14	0.63	1.00	0.66	0.70	0.69
Scanorama	0.57	0.92	0.17	0.98	0.31	0.93	0.96	0.61	0.11	0.59	1.00	0.59	0.70	0.65
cFIT	0.20	0.77	0.27	0.92	0.33	0.83	0.76	0.65	0.22	0.65	1.00	0.50	0.69	0.61
INSCT	0.35	0.67	0.29	0.80	0.43	0.72	0.55	0.65	0.06	0.44	1.00	0.51	0.57	0.55

Fig.S7 Performance of benchmarking methods for data integration on the pancreas data. a, Visualization based on the integration results of benchmarking methods. UMAP plots are colored by cell identity annotations (top) and batch labels (bottom). b, Metrics for evaluating the integration performance for benchmarking methods.

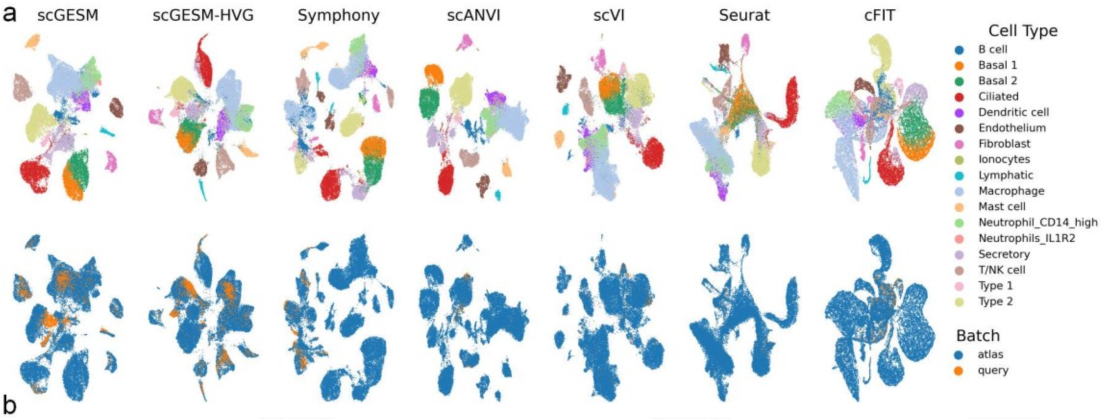

b

Method	Batch correction: PCR batch	Batch correction: Batch ASW	Batch correction: Graph iLISI	Batch correction: Graph connectivity	Batch correction: kBET	Bio-conservation: NMI cluster/label	Bio-conservation: ARI cluster/label	Bio-conservation: Cell type ASW	Bio-conservation: Isolated label F1	Bio-conservation: Isolated label silhouette	Bio-conservation: Graph cLISI	Aggregate score: Batch correction	Aggregate score: Bio-conservation	Aggregate score: Total
scGESM	0.88	0.83	0.01	0.99	0.65	0.80	0.68	0.62	0.72	0.59	1.00	0.67	0.74	0.71
scANVI	0.72	0.88	0.01	0.97	0.63	0.84	0.66	0.61	0.72	0.58	1.00	0.64	0.74	0.70
scGESM-HVG	0.93	0.80	0.02	0.97	0.65	0.74	0.58	0.59	0.62	0.54	0.99	0.67	0.68	0.68
scVI	0.87	0.89	0.03	0.97	0.66	0.71	0.58	0.54	0.60	0.54	0.98	0.68	0.66	0.67
Seurat	0.88	0.85	0.10	0.88	0.69	0.62	0.50	0.52	0.36	0.48	0.96	0.68	0.58	0.62
cFIT	0.00	0.66	0.00	0.96	0.61	0.68	0.43	0.53	0.67	0.59	0.99	0.45	0.65	0.57
Symphony	0.00	0.56	0.00	0.86	0.54	0.70	0.46	0.50	0.64	0.57	0.99	0.39	0.64	0.54

Fig.S8 Performance of benchmarking methods for mapping the B1 batch onto the reference constructed using other batches in the human lung data. a, Visualization based on the mapping results of benchmarking methods. UMAP plots are colored by cell identity annotations (top) and batch labels (bottom). b, Metrics for evaluating the mapping performance for benchmarking methods.

Method	Batch correction: PCR batch	Batch correction: Batch ASW	Batch correction: Graph iLISI	Batch correction: Graph connectivity	Batch correction: kBET	Bio-conservation: NMI cluster/label	Bio-conservation: ARI cluster/label	Bio-conservation: Cell type ASW	Bio-conservation: Isolated label F1	Bio-conservation: Isolated label silhouette	Bio-conservation: Graph cLISI	Aggregate score: Batch correction	Aggregate score: Bio-conservation	Aggregate score: Total
scGESM	0.90	0.82	0.04	0.98	0.45	0.80	0.62	0.62	0.68	0.57	1.00	0.64	0.72	0.68
scANVI	0.53	0.87	0.02	0.97	0.41	0.83	0.76	0.61	0.80	0.58	1.00	0.56	0.76	0.68
scGESM-HVG	0.95	0.80	0.04	0.96	0.45	0.71	0.56	0.59	0.65	0.55	0.99	0.64	0.68	0.66
Seurat	0.97	0.86	0.10	0.88	0.59	0.63	0.49	0.52	0.36	0.48	0.96	0.68	0.57	0.62
scVI	0.43	0.86	0.02	0.97	0.38	0.69	0.51	0.55	0.60	0.54	0.98	0.53	0.64	0.60
cFIT	0.00	0.65	0.00	0.97	0.43	0.71	0.52	0.54	0.65	0.56	0.99	0.41	0.66	0.56
Symphony	0.00	0.55	0.00	0.84	0.25	0.70	0.45	0.50	0.63	0.57	0.99	0.33	0.64	0.52

Fig.S9 Performance of benchmarking methods for mapping the B2 batch onto the reference constructed using other batches in the human lung data. Metrics for evaluating the mapping performance for benchmarking methods.

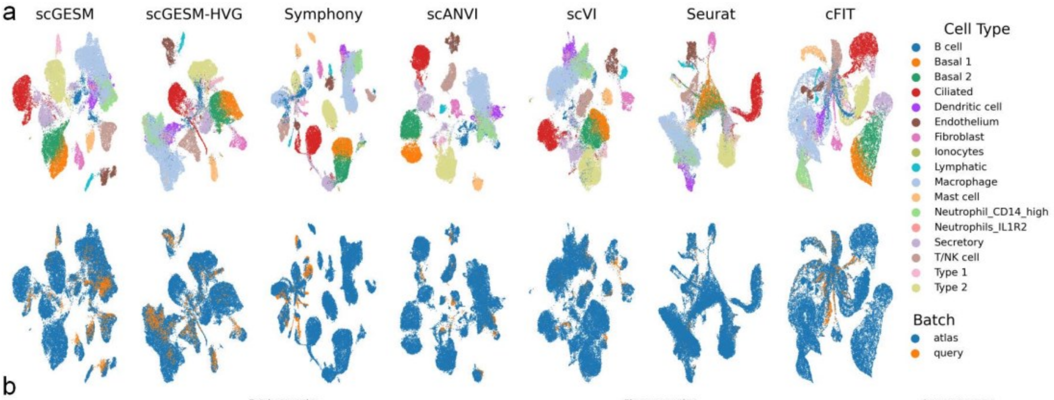

b

Method	Batch correction: PCR batch	Batch correction: Batch ASW	Batch correction: Graph iLISI	Batch correction: Graph connectivity	Batch correction: kBET	Bio-conservation: NMI cluster/label	Bio-conservation: ARI cluster/label	Bio-conservation: Cell type ASW	Bio-conservation: Isolated label F1	Bio-conservation: Isolated label silhouette	Bio-conservation: Graph cLISI	Aggregate score: Batch correction	Aggregate score: Bio-conservation	Aggregate score: Total
scGESM	0.85	0.78	0.01	0.99	0.58	0.81	0.73	0.62	0.71	0.58	1.00	0.64	0.74	0.70
scGESM-HVG	0.90	0.81	0.02	0.97	0.63	0.72	0.61	0.59	0.64	0.55	0.99	0.66	0.68	0.68
scANVI	0.40	0.84	0.01	0.97	0.54	0.83	0.68	0.62	0.69	0.58	1.00	0.55	0.73	0.66
scVI	0.44	0.85	0.01	0.97	0.56	0.70	0.52	0.54	0.61	0.54	0.98	0.57	0.65	0.62
Seurat	0.82	0.83	0.06	0.88	0.65	0.63	0.47	0.52	0.36	0.48	0.96	0.65	0.57	0.60
cFIT	0.00	0.61	0.00	0.97	0.52	0.70	0.46	0.55	0.65	0.57	0.99	0.42	0.65	0.56
Symphony	0.00	0.53	0.00	0.84	0.46	0.71	0.46	0.50	0.64	0.57	0.99	0.37	0.65	0.53

Fig.S10 Performance of benchmarking methods for mapping the B3 batch onto the reference constructed using other batches in the human lung data. a, Visualization based on the mapping results of benchmarking methods. UMAP plots are colored by cell identity annotations (top) and batch labels (bottom). b, Metrics for evaluating the mapping performance for benchmarking methods.

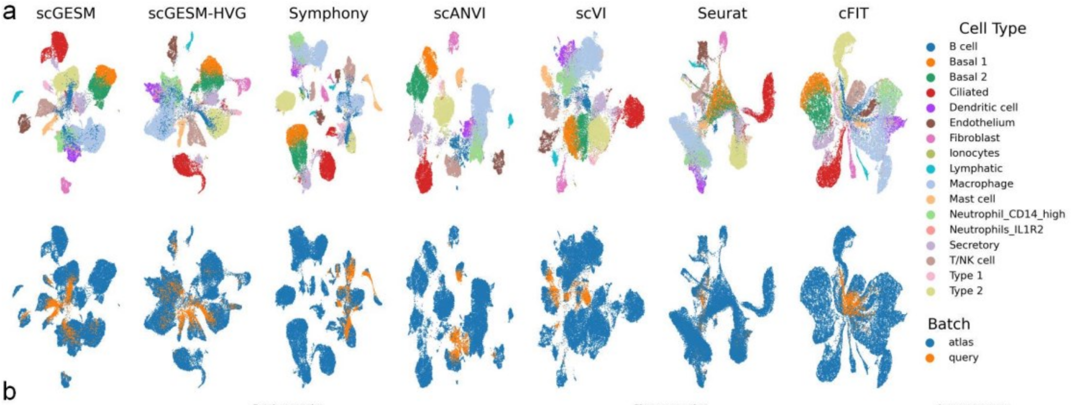

b

Method	Batch correction: PCR batch	Batch correction: Batch ASW	Batch correction: Graph iLISI	Batch correction: Graph connectivity	Batch correction: kBET	Bio-conservation: NMI cluster/label	Bio-conservation: ARI cluster/label	Bio-conservation: Cell type ASW	Bio-conservation: Isolated label F1	Bio-conservation: Isolated label silhouette	Bio-conservation: Graph cLISI	Aggregate score: Batch correction	Aggregate score: Bio-conservation	Aggregate score: Total
scGESM	0.88	0.79	0.00	0.98	0.61	0.79	0.65	0.62	0.69	0.58	1.00	0.65	0.72	0.69
scGESM-HVG	0.94	0.76	0.01	0.96	0.65	0.72	0.54	0.59	0.65	0.55	0.99	0.66	0.67	0.67
scANVI	0.28	0.77	0.00	0.98	0.56	0.82	0.68	0.60	0.71	0.57	1.00	0.52	0.73	0.64
Seurat	0.94	0.84	0.07	0.88	0.74	0.63	0.53	0.52	0.37	0.48	0.96	0.69	0.58	0.63
scVI	0.39	0.80	0.00	0.97	0.56	0.72	0.53	0.54	0.62	0.54	0.98	0.55	0.65	0.61
cFIT	0.00	0.70	0.00	0.96	0.66	0.71	0.48	0.55	0.54	0.56	0.99	0.46	0.64	0.57
Symphony	0.00	0.51	0.00	0.89	0.57	0.70	0.46	0.50	0.63	0.57	0.99	0.39	0.64	0.54

Fig.S11 Performance of benchmarking methods for mapping the B4 batch onto the reference constructed using other batches in the human lung data. a, Visualization based on the mapping results of benchmarking methods. UMAP plots are colored by cell identity annotations (top) and batch labels (bottom). b, Metrics for evaluating the mapping performance for benchmarking methods.

a

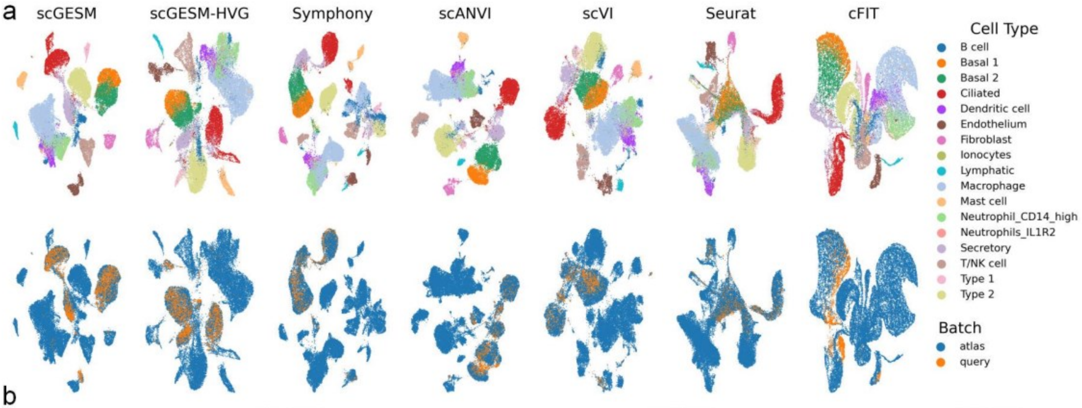

b

Method	Batch correction: PCR batch	Batch ASW	Graph iLISI	Graph connectivity	kBET	Bio-conservation: NMI cluster/label	ARI cluster/label	Cell type ASW	Isolated label F1	Isolated label silhouette	Graph cLISI	Aggregate score: Batch correction	Bio-conservation	Total
scGESM	0.74	0.82	0.00	0.99	0.73	0.82	0.69	0.62	0.84	0.71	1.00	0.66	0.78	0.73
scGESM-HVG	0.91	0.80	0.00	0.97	0.73	0.72	0.59	0.59	0.80	0.67	0.99	0.68	0.73	0.71
scANVI	0.69	0.80	0.00	0.97	0.70	0.80	0.67	0.60	0.77	0.66	1.00	0.63	0.75	0.70
scVI	0.76	0.82	0.00	0.96	0.71	0.73	0.61	0.54	0.78	0.63	0.98	0.65	0.71	0.69
Seurat	0.85	0.82	0.01	0.88	0.76	0.63	0.50	0.52	0.67	0.60	0.96	0.66	0.65	0.65
cFIT	0.61	0.72	0.00	0.95	0.58	0.68	0.46	0.54	0.73	0.54	0.99	0.57	0.66	0.62
Symphony	0.01	0.74	0.00	0.87	0.76	0.70	0.46	0.50	0.70	0.55	0.99	0.48	0.65	0.58

Fig.S12 Performance of benchmarking methods for mapping the A6 batch onto the reference constructed using other batches in the human lung data. a, Visualization based on the mapping results of benchmarking methods. UMAP plots are colored by cell identity annotations (top) and batch labels (bottom). b, Metrics for evaluating the mapping performance for benchmarking methods.

a

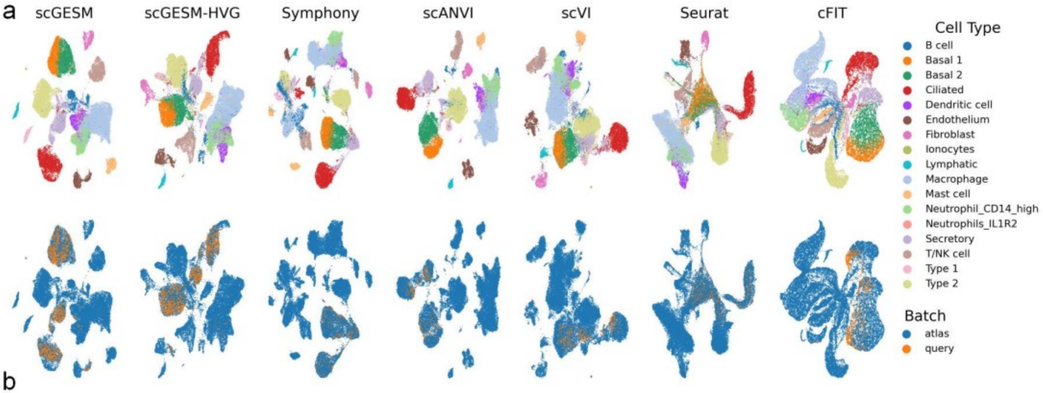

b

Method	Batch correction: PCR batch	Batch ASW	Graph iLISI	Graph connectivity	kBET	Bio-conservation: NMI cluster/label	ARI cluster/label	Cell type ASW	Isolated label F1	Isolated label silhouette	Graph cLISI	Aggregate score: Batch correction	Bio-conservation	Total
scGESM	0.62	0.82	0.00	0.98	0.79	0.83	0.65	0.63	0.85	0.71	1.00	0.64	0.78	0.72
scANVI	0.51	0.85	0.00	0.97	0.78	0.81	0.66	0.60	0.81	0.68	1.00	0.62	0.76	0.70
scGESM-HVG	0.78	0.80	0.00	0.97	0.79	0.73	0.54	0.60	0.80	0.66	0.99	0.67	0.72	0.70
scVI	0.53	0.85	0.00	0.97	0.74	0.70	0.54	0.54	0.77	0.62	0.98	0.62	0.69	0.66
cFIT	0.60	0.72	0.00	0.97	0.74	0.72	0.53	0.54	0.76	0.59	1.00	0.61	0.69	0.66
Seurat	0.80	0.83	0.01	0.88	0.74	0.63	0.49	0.52	0.66	0.60	0.96	0.65	0.65	0.65
Symphony	0.08	0.74	0.00	0.90	0.82	0.70	0.46	0.49	0.67	0.55	0.99	0.51	0.64	0.59

Fig.S13 Performance of benchmarking methods for mapping the A1 batch onto the reference constructed using other batches in the human lung data. a, Visualization based on the mapping results of benchmarking methods. UMAP plots are colored by cell identity annotations (top) and batch labels (bottom). b, Metrics for evaluating the mapping performance for benchmarking methods.

a

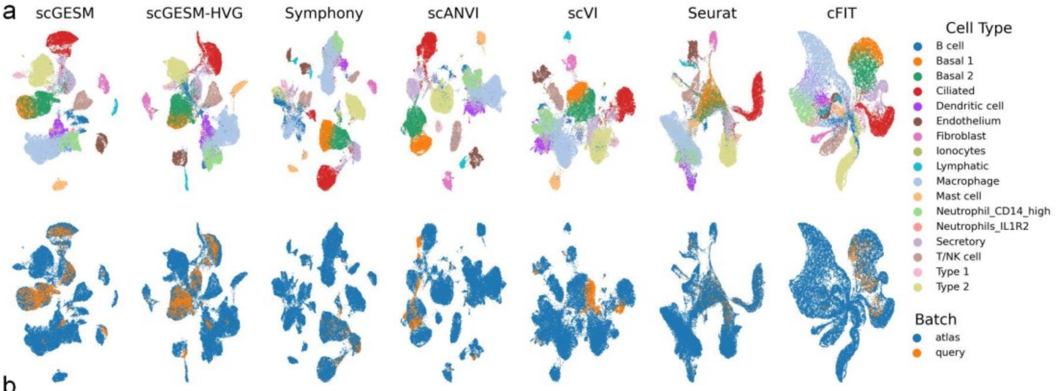

b

Method	Batch correction: PCR batch	Batch correction: Batch ASW	Batch correction: Graph iLISI	Batch correction: Graph connectivity	Batch correction: kBET	Bio-conservation: NMI cluster/label	Bio-conservation: ARI cluster/label	Bio-conservation: Cell type ASW	Bio-conservation: Isolated label F1	Bio-conservation: Isolated label silhouette	Bio-conservation: Graph cLISI	Aggregate score: Batch correction	Aggregate score: Bio-conservation	Aggregate score: Total
scGESM	0.75	0.84	0.00	0.98	0.66	0.81	0.65	0.62	0.77	0.65	1.00	0.65	0.75	0.71
scGESM-HVG	0.87	0.82	0.00	0.97	0.64	0.73	0.56	0.60	0.70	0.61	0.99	0.66	0.70	0.68
scANVI	0.29	0.83	0.00	0.97	0.63	0.81	0.64	0.60	0.71	0.63	1.00	0.54	0.73	0.66
Seurat	0.88	0.86	0.02	0.88	0.75	0.63	0.50	0.52	0.56	0.56	0.96	0.68	0.62	0.65
cFIT	0.53	0.77	0.00	0.96	0.65	0.70	0.51	0.54	0.71	0.61	0.99	0.58	0.68	0.64
scVI	0.23	0.83	0.00	0.96	0.64	0.70	0.54	0.54	0.65	0.58	0.98	0.53	0.67	0.61
Symphony	0.16	0.72	0.00	0.91	0.72	0.70	0.47	0.50	0.65	0.56	0.99	0.50	0.65	0.59

Fig.S14 Performance of benchmarking methods for mapping the A5 batch onto the reference constructed using other batches in the human lung data. a, Visualization based on the mapping results of benchmarking methods. UMAP plots are colored by cell identity annotations (top) and batch labels (bottom). b, Metrics for evaluating the mapping performance for benchmarking methods.

a

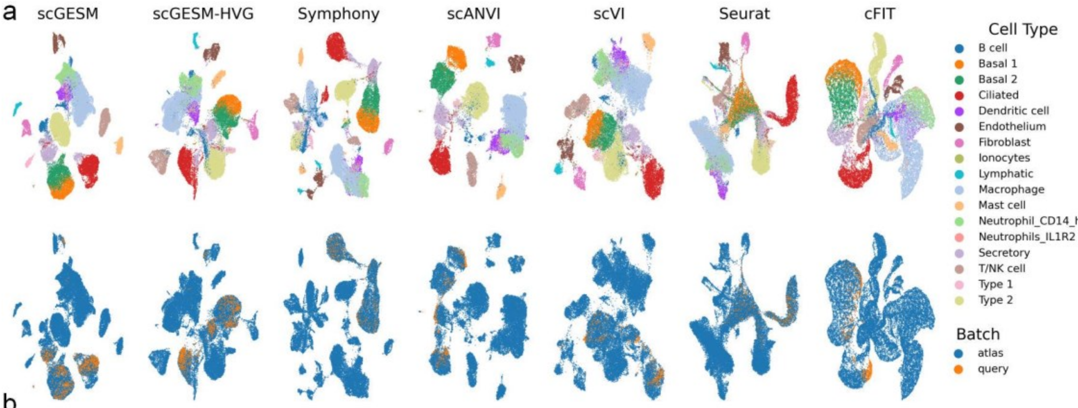

b

Method	Batch correction: PCR batch	Batch correction: Batch ASW	Batch correction: Graph iLISI	Batch correction: Graph connectivity	Batch correction: kBET	Bio-conservation: NMI cluster/label	Bio-conservation: ARI cluster/label	Bio-conservation: Cell type ASW	Bio-conservation: Isolated label F1	Bio-conservation: Isolated label silhouette	Bio-conservation: Graph cLISI	Aggregate score: Batch correction	Aggregate score: Bio-conservation	Aggregate score: Total
scGESM	0.50	0.85	0.00	0.98	0.80	0.81	0.65	0.63	0.86	0.70	1.00	0.63	0.78	0.72
scGESM-HVG	0.79	0.83	0.00	0.97	0.79	0.73	0.54	0.60	0.80	0.65	0.99	0.67	0.72	0.70
scANVI	0.36	0.83	0.00	0.97	0.74	0.81	0.68	0.61	0.78	0.66	1.00	0.58	0.76	0.69
scVI	0.41	0.84	0.00	0.97	0.74	0.71	0.56	0.54	0.76	0.59	0.98	0.59	0.69	0.65
cFIT	0.57	0.73	0.00	0.97	0.73	0.70	0.46	0.54	0.73	0.61	0.99	0.60	0.67	0.64
Seurat	0.69	0.80	0.00	0.88	0.81	0.62	0.50	0.52	0.65	0.58	0.96	0.64	0.64	0.64
Symphony	0.00	0.73	0.00	0.89	0.79	0.70	0.45	0.50	0.68	0.57	0.99	0.48	0.65	0.58

Fig.S15 Performance of benchmarking methods for mapping the A3 batch onto the reference constructed using other batches in the human lung data. a, Visualization based on the mapping results of benchmarking methods. UMAP plots are colored by cell identity annotations (top) and batch labels (bottom). b, Metrics for evaluating the mapping performance for benchmarking methods.

a

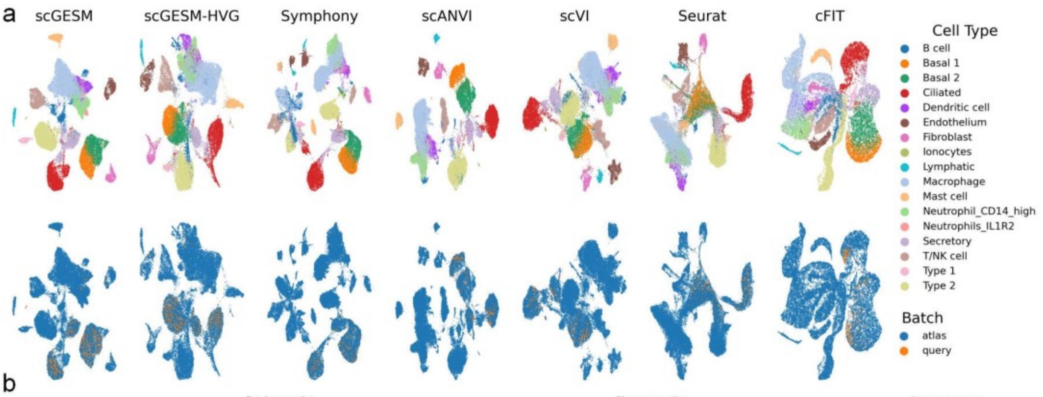

b

Method	Batch correction					Bio-conservation						Aggregate score		
	PCR batch	Batch ASW	Graph iLISI	Graph connectivity	kBET	NMI cluster/label	ARI cluster/label	Cell type ASW	Isolated label F1	Isolated label silhouette	Graph cLISI	Batch correction	Bio-conservation	Total
scGESM	0.56	0.81	0.00	0.99	0.86	0.81	0.69	0.63	0.87	0.67	1.00	0.64	0.78	0.72
scGESM-HVG	0.81	0.79	0.00	0.97	0.86	0.74	0.59	0.60	0.79	0.65	0.99	0.69	0.72	0.71
scANVI	0.44	0.83	0.00	0.97	0.82	0.80	0.64	0.61	0.78	0.65	1.00	0.61	0.75	0.69
cFIT	0.55	0.76	0.00	0.98	0.81	0.72	0.53	0.56	0.79	0.58	1.00	0.62	0.70	0.66
scVI	0.50	0.84	0.00	0.97	0.81	0.70	0.56	0.54	0.76	0.59	0.98	0.62	0.69	0.66
Seurat	0.73	0.81	0.00	0.88	0.83	0.63	0.49	0.52	0.67	0.57	0.96	0.65	0.64	0.64
Symphony	0.00	0.76	0.00	0.87	0.85	0.71	0.46	0.50	0.65	0.52	0.99	0.49	0.64	0.58

Fig.S16 Performance of benchmarking methods for mapping the A4 batch onto the reference constructed using other batches in the human lung data. a, Visualization based on the mapping results of benchmarking methods. UMAP plots are colored by cell identity annotations (top) and batch labels (bottom). b, Metrics for evaluating the mapping performance for benchmarking methods.

a

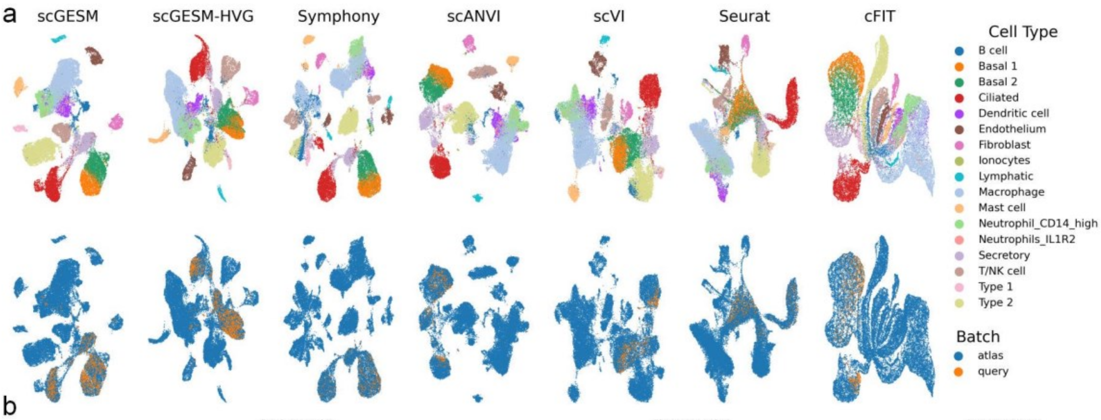

b

Method	Batch correction					Bio-conservation						Aggregate score		
	PCR batch	Batch ASW	Graph iLISI	Graph connectivity	kBET	NMI cluster/label	ARI cluster/label	Cell type ASW	Isolated label F1	Isolated label silhouette	Graph cLISI	Batch correction	Bio-conservation	Total
scGESM	0.49	0.85	0.00	0.98	0.82	0.83	0.74	0.63	0.99	0.73	1.00	0.63	0.82	0.74
scGESM-HVG	0.77	0.80	0.00	0.97	0.81	0.72	0.54	0.59	0.94	0.70	0.99	0.67	0.75	0.72
scANVI	0.32	0.82	0.00	0.97	0.77	0.79	0.63	0.60	0.94	0.71	0.99	0.58	0.78	0.70
Seurat	0.80	0.81	0.00	0.88	0.77	0.63	0.49	0.52	0.84	0.61	0.96	0.65	0.67	0.67
scVI	0.43	0.81	0.00	0.97	0.75	0.70	0.53	0.55	0.87	0.65	0.98	0.59	0.71	0.67
cFIT	0.43	0.74	0.00	0.96	0.76	0.71	0.48	0.54	0.85	0.60	0.99	0.58	0.70	0.65
Symphony	0.00	0.74	0.00	0.84	0.80	0.70	0.47	0.50	0.77	0.54	0.99	0.48	0.66	0.59

Fig.S17 Performance of benchmarking methods for mapping the A2 batch onto the reference constructed using other batches in the human lung data. a, Visualization based on the mapping results of benchmarking methods. UMAP plots are colored by cell identity annotations (top) and batch labels (bottom). b, Metrics for evaluating the mapping performance for benchmarking methods.

a

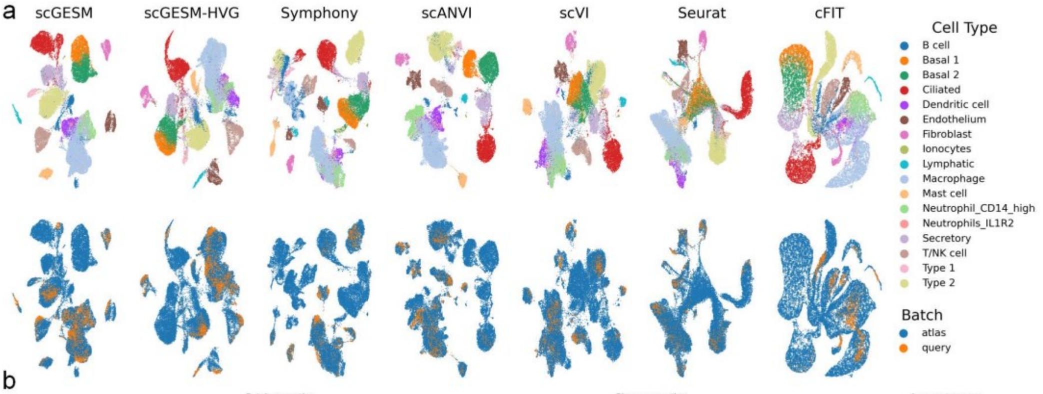

b

Method	Batch correction: PCR batch	Batch correction: Batch ASW	Batch correction: Graph iLISI	Batch correction: Graph connectivity	Batch correction: kBET	Bio-conservation: NMI cluster/label	Bio-conservation: ARI cluster/label	Bio-conservation: Cell type ASW	Bio-conservation: Isolated label F1	Bio-conservation: Isolated label silhouette	Bio-conservation: Graph cLISI	Aggregate score: Batch correction	Aggregate score: Bio-conservation	Aggregate score: Total
scGESM	0.70	0.81	0.02	0.99	0.63	0.82	0.70	0.64	0.93	0.75	1.00	0.63	0.81	0.74
scANVI	0.67	0.85	0.03	0.97	0.68	0.81	0.69	0.61	0.86	0.67	1.00	0.64	0.77	0.72
scGESM-HVG	0.83	0.76	0.02	0.97	0.59	0.72	0.54	0.60	0.81	0.70	0.99	0.63	0.73	0.69
scVI	0.66	0.87	0.04	0.96	0.66	0.70	0.53	0.54	0.82	0.66	0.98	0.64	0.70	0.68
cFIT	0.68	0.75	0.00	0.97	0.55	0.72	0.51	0.55	0.80	0.64	0.99	0.59	0.70	0.66
Symphony	0.11	0.68	0.00	0.87	0.60	0.70	0.46	0.50	0.87	0.74	0.99	0.45	0.71	0.61
Seurat	0.76	0.81	0.04	0.88	0.71	0.62	0.49	0.52	0.35	0.56	0.96	0.64	0.59	0.61

Fig.S18 Performance of benchmarking methods for mapping the 5 batch onto the reference constructed using other batches in the human lung data. a, Visualization based on the mapping results of benchmarking methods. UMAP plots are colored by cell identity annotations (top) and batch labels (bottom). b, Metrics for evaluating the mapping performance for benchmarking methods.

a

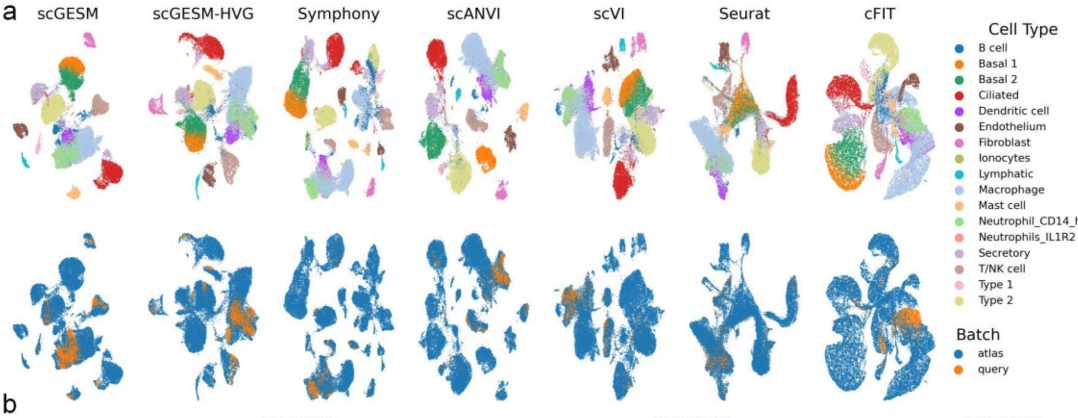

b

Method	Batch correction: PCR batch	Batch correction: Batch ASW	Batch correction: Graph iLISI	Batch correction: Graph connectivity	Batch correction: kBET	Bio-conservation: NMI cluster/label	Bio-conservation: ARI cluster/label	Bio-conservation: Cell type ASW	Bio-conservation: Isolated label F1	Bio-conservation: Isolated label silhouette	Bio-conservation: Graph cLISI	Aggregate score: Batch correction	Aggregate score: Bio-conservation	Aggregate score: Total
scGESM	0.83	0.78	0.00	0.99	0.69	0.80	0.64	0.61	0.93	0.73	1.00	0.66	0.78	0.73
scGESM-HVG	0.88	0.77	0.00	0.96	0.70	0.73	0.59	0.58	0.88	0.67	0.99	0.66	0.74	0.71
scANVI	0.39	0.85	0.00	0.97	0.73	0.80	0.64	0.61	0.95	0.70	1.00	0.59	0.78	0.71
scVI	0.47	0.84	0.00	0.96	0.72	0.71	0.54	0.54	0.87	0.64	0.98	0.60	0.72	0.67
cFIT	0.69	0.76	0.00	0.97	0.66	0.71	0.50	0.54	0.76	0.65	1.00	0.62	0.69	0.66
Seurat	0.75	0.79	0.01	0.88	0.79	0.63	0.50	0.52	0.59	0.59	0.96	0.64	0.63	0.64
Symphony	0.30	0.70	0.00	0.90	0.70	0.71	0.47	0.51	0.78	0.62	0.99	0.52	0.68	0.62

Fig.S19 Performance of benchmarking methods for mapping the 2 batch onto the reference constructed using other batches in the human lung data. a, Visualization based on the mapping results of benchmarking methods. UMAP plots are colored by cell identity annotations (top) and batch labels (bottom). b, Metrics for evaluating the mapping performance for benchmarking methods.

a

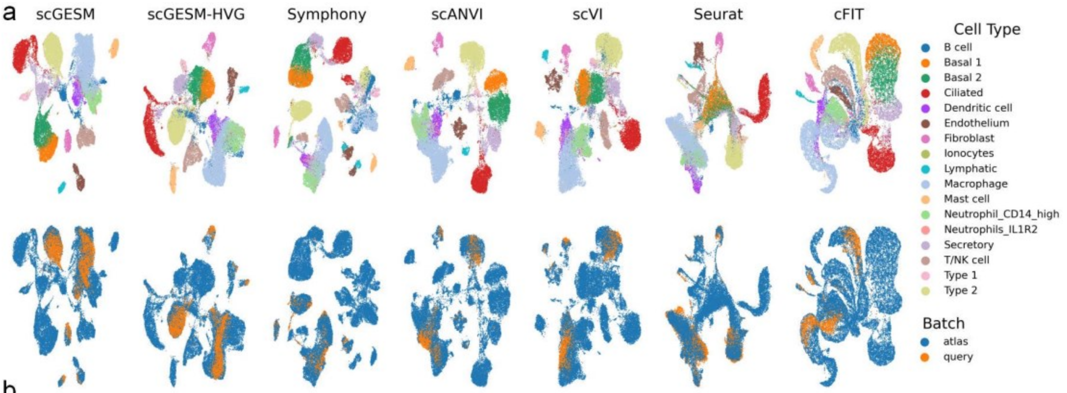

b

Method	Batch correction: PCR batch	Batch ASW	Graph iLISI	Graph connectivity	kBET	Bio-conservation: NMI cluster/label	ARI cluster/label	Cell type ASW	Isolated label F1	Isolated label silhouette	Graph cLISI	Aggregate score: Batch correction	Bio-conservation	Total
scGESM	0.60	0.82	0.01	0.99	0.69	0.80	0.64	0.64	0.80	0.70	1.00	0.62	0.76	0.71
scGESM-HVG	0.65	0.79	0.02	0.96	0.67	0.73	0.60	0.62	0.75	0.65	0.99	0.62	0.72	0.68
scANVI	0.20	0.84	0.01	0.97	0.68	0.82	0.67	0.61	0.78	0.65	1.00	0.54	0.76	0.67
scVI	0.29	0.85	0.01	0.97	0.70	0.74	0.61	0.56	0.73	0.64	0.99	0.56	0.71	0.65
cFIT	0.32	0.72	0.00	0.98	0.65	0.70	0.50	0.54	0.70	0.63	0.99	0.53	0.68	0.62
Seurat	0.87	0.68	0.01	0.88	0.71	0.62	0.50	0.52	0.41	0.55	0.96	0.63	0.59	0.61
Symphony	0.09	0.65	0.00	0.86	0.69	0.71	0.48	0.51	0.76	0.66	0.99	0.46	0.69	0.60

Fig.S20 Performance of benchmarking methods for mapping the 4 batch onto the reference constructed using other batches in the human lung data. a, Visualization based on the mapping results of benchmarking methods. UMAP plots are colored by cell identity annotations (top) and batch labels (bottom). b, Metrics for evaluating the mapping performance for benchmarking methods.

a

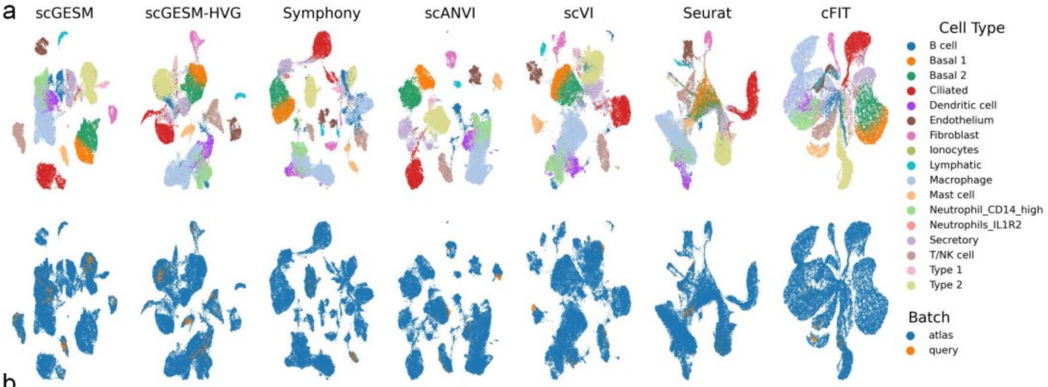

b

Method	Batch correction: PCR batch	Batch ASW	Graph iLISI	Graph connectivity	kBET	Bio-conservation: NMI cluster/label	ARI cluster/label	Cell type ASW	Isolated label F1	Isolated label silhouette	Graph cLISI	Aggregate score: Batch correction	Bio-conservation	Total
scGESM	0.66	0.83	0.00	0.98	0.86	0.81	0.63	0.63	0.94	0.68	1.00	0.67	0.78	0.74
scGESM-HVG	0.74	0.78	0.00	0.97	0.84	0.73	0.56	0.59	0.84	0.64	0.99	0.67	0.73	0.70
scANVI	0.07	0.86	0.00	0.97	0.81	0.83	0.68	0.60	0.94	0.64	1.00	0.54	0.78	0.69
Seurat	0.86	0.82	0.00	0.88	0.89	0.62	0.49	0.52	0.58	0.55	0.96	0.69	0.62	0.65
scVI	0.00	0.87	0.00	0.97	0.80	0.73	0.60	0.55	0.82	0.61	0.98	0.53	0.71	0.64
Symphony	0.39	0.71	0.00	0.87	0.86	0.71	0.46	0.51	0.75	0.57	0.99	0.57	0.67	0.63
cFIT	0.44	0.74	0.00	0.96	0.79	0.70	0.47	0.54	0.61	0.56	0.99	0.59	0.64	0.62

Fig.S21 Performance of benchmarking methods for mapping the 3 batch onto the reference constructed using other batches in the human lung data. a, Visualization based on the mapping results of benchmarking methods. UMAP plots are colored by cell identity annotations (top) and batch labels (bottom). b, Metrics for evaluating the mapping performance for benchmarking methods.

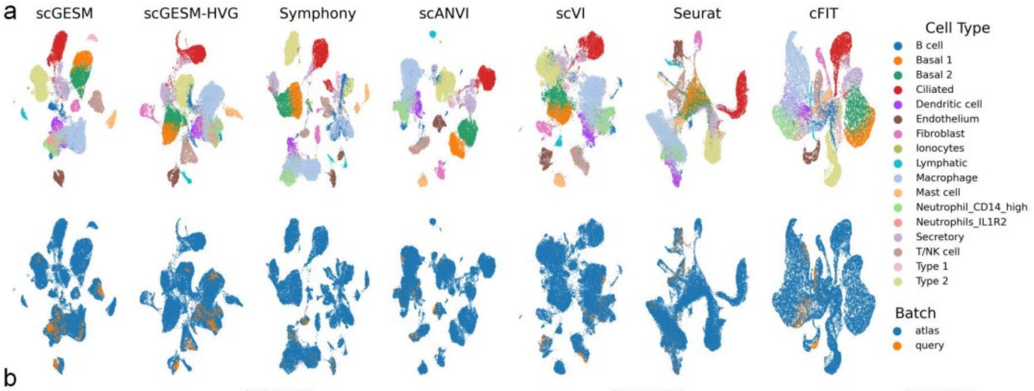

b

	Batch correction					Bio-conservation						Aggregate score		
Method	PCR batch	Batch ASW	Graph iLISI	Graph connectivity	kBET	NMI cluster/label	ARI cluster/label	Cell type ASW	Isolated label F1	Isolated label silhouette	Graph cLISI	Batch correction	Bio-conservation	Total
scGESM	0.77	0.84	0.00	0.98	0.78	0.81	0.66	0.63	0.94	0.71	1.00	0.68	0.79	0.75
scANVI	0.47	0.85	0.00	0.97	0.78	0.81	0.66	0.60	0.94	0.69	1.00	0.61	0.78	0.72
scGESM-HVG	0.84	0.79	0.00	0.96	0.79	0.72	0.58	0.59	0.89	0.63	0.99	0.67	0.73	0.71
scVI	0.46	0.84	0.00	0.97	0.78	0.70	0.59	0.55	0.86	0.65	0.98	0.61	0.72	0.68
cFIT	0.61	0.72	0.00	0.96	0.77	0.70	0.46	0.54	0.85	0.63	0.99	0.61	0.70	0.66
Seurat	0.77	0.83	0.00	0.88	0.84	0.62	0.46	0.52	0.61	0.59	0.96	0.66	0.63	0.64
Symphony	0.42	0.68	0.00	0.90	0.77	0.71	0.46	0.50	0.84	0.65	0.99	0.55	0.69	0.64

Fig.S22 Performance of benchmarking methods for mapping the 1 batch onto the reference constructed using other batches in the human lung data. a, Visualization based on the mapping results of benchmarking methods. UMAP plots are colored by cell identity annotations (top) and batch labels (bottom). b, Metrics for evaluating the mapping performance for benchmarking methods.

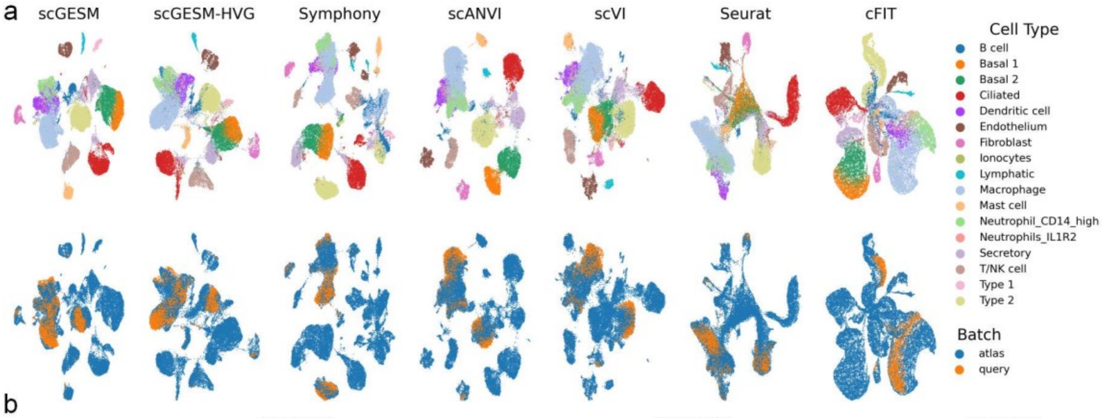

b

	Batch correction					Bio-conservation						Aggregate score		
Method	PCR batch	Batch ASW	Graph iLISI	Graph connectivity	kBET	NMI cluster/label	ARI cluster/label	Cell type ASW	Isolated label F1	Isolated label silhouette	Graph cLISI	Batch correction	Bio-conservation	Total
scGESM	0.69	0.79	0.01	0.99	0.60	0.82	0.65	0.64	0.94	0.68	1.00	0.61	0.79	0.72
scANVI	0.49	0.83	0.01	0.97	0.62	0.80	0.64	0.61	0.92	0.67	1.00	0.58	0.77	0.70
scGESM-HVG	0.79	0.79	0.01	0.97	0.60	0.74	0.58	0.60	0.82	0.63	0.99	0.63	0.73	0.69
scVI	0.71	0.85	0.02	0.97	0.62	0.70	0.54	0.55	0.83	0.61	0.98	0.63	0.70	0.67
Seurat	0.78	0.82	0.03	0.88	0.71	0.64	0.53	0.52	0.57	0.56	0.96	0.65	0.63	0.64
cFIT	0.50	0.70	0.00	0.97	0.54	0.69	0.50	0.54	0.73	0.60	1.00	0.54	0.68	0.62
Symphony	0.14	0.63	0.00	0.86	0.60	0.70	0.46	0.51	0.79	0.60	0.99	0.45	0.68	0.58

Fig.S23 Performance of benchmarking methods for mapping the 6 batch onto the reference constructed using other batches in the human lung data. a, Visualization based on the mapping results of benchmarking methods. UMAP plots are colored by cell identity annotations (top) and batch labels (bottom). b, Metrics for evaluating the mapping performance for benchmarking methods.

a

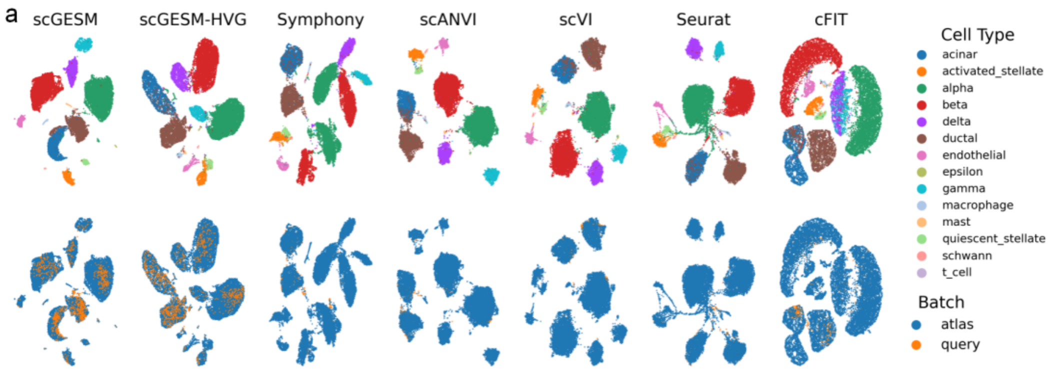

b

	Batch correction					Bio-conservation						Aggregate score		
Method	PCR batch	Batch ASW	Graph iLISI	Graph connectivity	kBET	NMI cluster/label	ARI cluster/label	Cell type ASW	Isolated label F1	Isolated label silhouette	Graph cLISI	Batch correction	Bio-conservation	Total
scGESM	0.84	0.82	0.03	0.99	0.86	0.95	0.97	0.67	0.13	0.81	1.00	0.71	0.75	0.73
scGESM-HVG	0.80	0.82	0.03	0.97	0.86	0.91	0.95	0.73	0.10	0.56	1.00	0.70	0.71	0.70
Seurat	0.56	0.86	0.06	0.98	0.88	0.89	0.91	0.69	0.08	0.72	1.00	0.67	0.72	0.70
scANVI	0.46	0.84	0.03	0.98	0.84	0.93	0.96	0.64	0.14	0.71	1.00	0.63	0.73	0.69
scVI	0.44	0.84	0.03	0.98	0.84	0.91	0.95	0.60	0.13	0.72	1.00	0.63	0.72	0.68
cFIT	0.72	0.74	0.02	0.93	0.65	0.88	0.92	0.64	0.10	0.63	1.00	0.61	0.70	0.66
Symphony	0.48	0.84	0.03	0.96	0.74	0.82	0.65	0.61	0.10	0.52	1.00	0.61	0.62	0.61

Fig.S24 Performance of benchmarking methods for mapping the celseq batch onto the reference constructed using other batches in the human pancreas data. a, Visualization based on the mapping results of benchmarking methods. UMAP plots are colored by cell identity annotations (top) and batch labels (bottom). b, Metrics for evaluating the mapping performance for benchmarking methods.

a

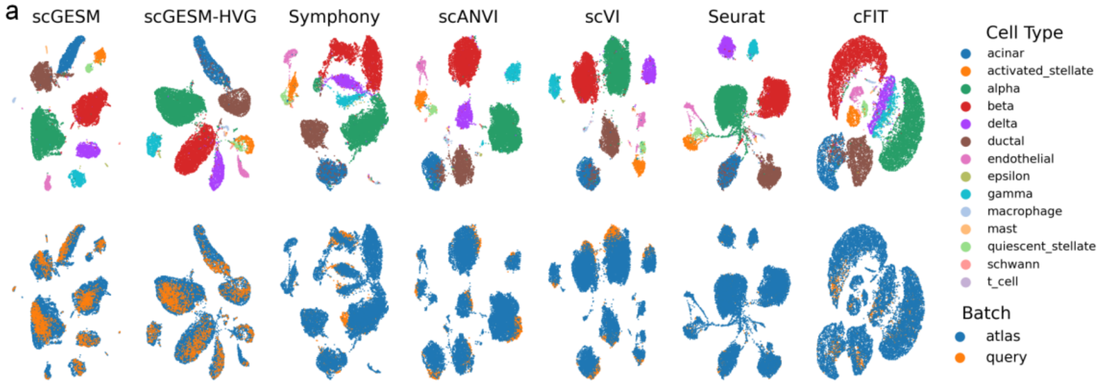

b

	Batch correction					Bio-conservation						Aggregate score		
Method	PCR batch	Batch ASW	Graph iLISI	Graph connectivity	kBET	NMI cluster/label	ARI cluster/label	Cell type ASW	Isolated label F1	Isolated label silhouette	Graph cLISI	Batch correction	Bio-conservation	Total
scGESM	0.97	0.82	0.20	0.99	0.65	0.94	0.97	0.67	0.25	0.83	1.00	0.73	0.78	0.76
Seurat	0.99	0.89	0.22	0.98	0.76	0.89	0.91	0.69	0.08	0.72	1.00	0.77	0.72	0.74
scGESM-HVG	0.98	0.81	0.22	0.98	0.60	0.91	0.94	0.72	0.09	0.59	1.00	0.72	0.71	0.71
scANVI	0.55	0.86	0.06	0.98	0.44	0.93	0.96	0.63	0.24	0.71	1.00	0.58	0.74	0.68
cFIT	0.77	0.77	0.15	0.93	0.43	0.87	0.92	0.66	0.09	0.69	1.00	0.61	0.71	0.67
Symphony	0.90	0.85	0.11	0.95	0.48	0.83	0.81	0.64	0.08	0.52	1.00	0.66	0.65	0.65
scVI	0.29	0.86	0.04	0.98	0.40	0.90	0.94	0.60	0.15	0.73	1.00	0.52	0.72	0.64

Fig.S25 Performance of benchmarking methods for mapping the CelSeq2 batch onto the reference constructed using other batches in the human pancreas data. a, Visualization based on the mapping results of benchmarking methods. UMAP plots are colored by cell identity annotations (top) and batch labels (bottom). b, Metrics for evaluating the mapping performance for benchmarking methods.

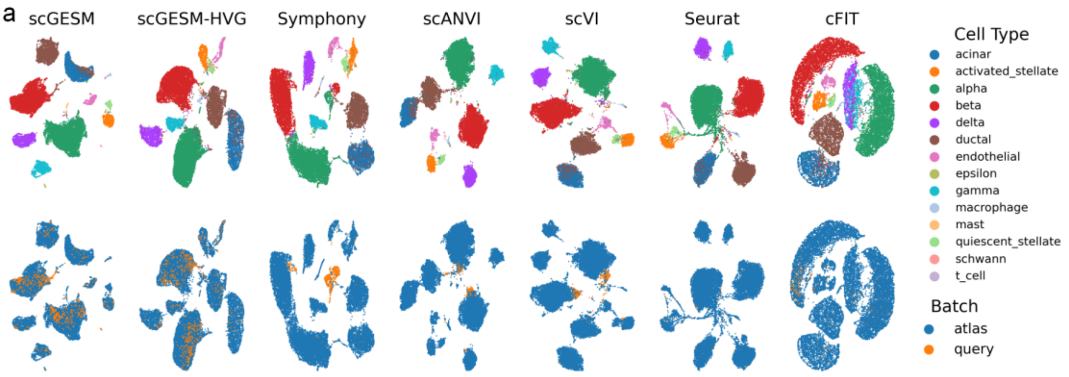

b

Method	PCR batch	Batch ASW	Graph iLISI	Graph connectivity	kBET	NMI cluster/label	ARI cluster/label	Cell type ASW	Isolated label F1	Isolated label silhouette	Graph cLISI	Batch correction	Bio-conservation	Total
	Batch correction					Bio-conservation						Aggregate score		
scGESM	0.81	0.73	0.02	0.99	0.92	0.94	0.97	0.66	0.19	0.79	1.00	0.70	0.76	0.73
Seurat	0.86	0.88	0.01	0.98	0.93	0.89	0.91	0.69	0.08	0.72	1.00	0.73	0.72	0.72
scGESM-HVG	0.94	0.77	0.02	0.98	0.83	0.91	0.94	0.72	0.10	0.59	1.00	0.71	0.71	0.71
cFIT	0.59	0.74	0.01	0.93	0.78	0.88	0.93	0.66	0.10	0.66	1.00	0.61	0.70	0.67
scANVI	0.28	0.84	0.00	0.99	0.79	0.91	0.95	0.63	0.07	0.76	1.00	0.58	0.72	0.66
scVI	0.00	0.82	0.00	0.98	0.80	0.91	0.95	0.60	0.10	0.74	1.00	0.52	0.72	0.64
Symphony	0.00	0.56	0.00	0.96	0.82	0.87	0.90	0.65	0.08	0.52	1.00	0.47	0.67	0.59

Fig.S26 Performance of benchmarking methods for mapping the fluidigmc1 batch onto the reference constructed using other batches in the human pancreas data. a, Visualization based on the mapping results of benchmarking methods. UMAP plots are colored by cell identity annotations (top) and batch labels (bottom). b, Metrics for evaluating the mapping performance for benchmarking methods.

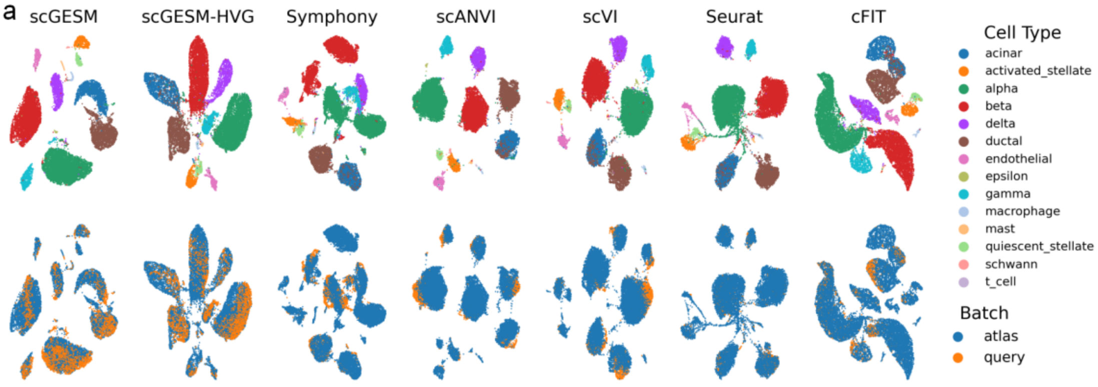

b

Method	PCR batch	Batch ASW	Graph iLISI	Graph connectivity	kBET	NMI cluster/label	ARI cluster/label	Cell type ASW	Isolated label F1	Isolated label silhouette	Graph cLISI	Batch correction	Bio-conservation	Total
	Batch correction					Bio-conservation						Aggregate score		
scGESM	0.91	0.81	0.16	1.00	0.66	0.95	0.97	0.68	0.18	0.83	1.00	0.71	0.77	0.74
Seurat	0.92	0.86	0.29	0.98	0.79	0.89	0.91	0.69	0.08	0.72	1.00	0.77	0.72	0.74
scGESM-HVG	0.91	0.80	0.17	0.98	0.55	0.91	0.94	0.73	0.08	0.62	1.00	0.68	0.71	0.70
cFIT	0.24	0.81	0.21	0.96	0.55	0.91	0.94	0.67	0.10	0.68	1.00	0.55	0.72	0.65
scANVI	0.07	0.88	0.08	0.99	0.49	0.93	0.95	0.62	0.15	0.76	1.00	0.50	0.73	0.64
scVI	0.00	0.89	0.08	0.98	0.59	0.91	0.94	0.60	0.12	0.70	1.00	0.51	0.71	0.63
Symphony	0.66	0.83	0.10	0.95	0.57	0.78	0.62	0.60	0.09	0.60	1.00	0.62	0.62	0.62

Fig.S27 Performance of benchmarking methods for mapping the smartseq2 batch onto the reference constructed using other batches in the human pancreas data. a, Visualization based on the mapping results of benchmarking methods. UMAP plots are colored by cell identity annotations (top) and batch labels (bottom). b, Metrics for evaluating the mapping performance for benchmarking methods.

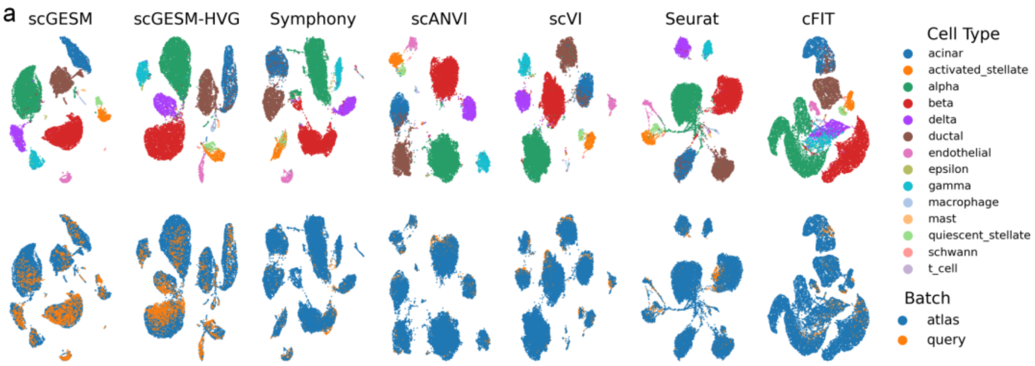

b

Method	Batch correction: PCR batch	Batch correction: Batch ASW	Batch correction: Graph iLISI	Batch correction: Graph connectivity	Batch correction: kBET	Bio-conservation: NMI cluster/label	Bio-conservation: ARI cluster/label	Bio-conservation: Cell type ASW	Bio-conservation: Isolated label F1	Bio-conservation: Isolated label silhouette	Bio-conservation: Graph cLISI	Aggregate score: Batch correction	Aggregate score: Bio-conservation	Aggregate score: Total
scGESM	0.84	0.86	0.13	0.99	0.64	0.95	0.97	0.66	0.89	0.71	1.00	0.69	0.86	0.79
scGESM-HVG	0.77	0.84	0.10	0.98	0.70	0.91	0.95	0.72	0.87	0.70	1.00	0.68	0.86	0.79
Seurat	0.76	0.88	0.19	0.98	0.67	0.89	0.91	0.69	0.72	0.69	1.00	0.70	0.82	0.77
scANVI	0.37	0.88	0.10	0.98	0.60	0.92	0.96	0.63	0.89	0.67	1.00	0.59	0.85	0.74
scVI	0.37	0.89	0.08	0.98	0.57	0.91	0.95	0.60	0.89	0.67	1.00	0.58	0.84	0.73
Symphony	0.49	0.84	0.13	0.96	0.67	0.88	0.91	0.65	0.69	0.63	1.00	0.62	0.79	0.72
cFIT	0.00	0.73	0.05	0.90	0.49	0.81	0.71	0.61	0.72	0.61	1.00	0.43	0.74	0.62

Fig.S28 Performance of benchmarking methods for mapping the inDrop1 batch onto the reference constructed using other batches in the human pancreas data. a, Visualization based on the mapping results of benchmarking methods. UMAP plots are colored by cell identity annotations (top) and batch labels (bottom). b, Metrics for evaluating the mapping performance for benchmarking methods.

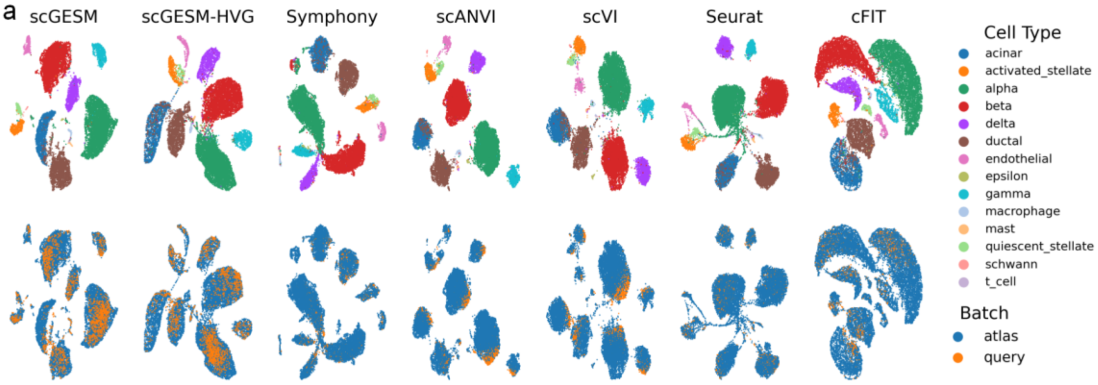

b

Method	Batch correction: PCR batch	Batch correction: Batch ASW	Batch correction: Graph iLISI	Batch correction: Graph connectivity	Batch correction: kBET	Bio-conservation: NMI cluster/label	Bio-conservation: ARI cluster/label	Bio-conservation: Cell type ASW	Bio-conservation: Isolated label F1	Bio-conservation: Isolated label silhouette	Bio-conservation: Graph cLISI	Aggregate score: Batch correction	Aggregate score: Bio-conservation	Aggregate score: Total
scGESM	0.96	0.83	0.14	0.99	0.79	0.94	0.96	0.67	0.84	0.74	1.00	0.74	0.86	0.81
scGESM-HVG	0.96	0.80	0.12	0.98	0.75	0.91	0.94	0.72	0.81	0.70	1.00	0.72	0.85	0.80
Seurat	0.92	0.87	0.11	0.98	0.85	0.89	0.91	0.69	0.72	0.69	1.00	0.75	0.82	0.79
Symphony	0.66	0.82	0.13	0.96	0.62	0.87	0.90	0.65	0.70	0.63	1.00	0.64	0.79	0.73
cFIT	0.49	0.75	0.13	0.95	0.51	0.91	0.94	0.64	0.72	0.61	1.00	0.57	0.80	0.71
scANVI	0.00	0.84	0.04	0.98	0.52	0.92	0.96	0.63	0.88	0.67	1.00	0.48	0.85	0.70
scVI	0.00	0.85	0.05	0.98	0.56	0.90	0.94	0.60	0.85	0.66	1.00	0.49	0.82	0.69

Fig.S29 Performance of benchmarking methods for mapping the inDrop2 batch onto the reference constructed using other batches in the human pancreas data. a, Visualization based on the mapping results of benchmarking methods. UMAP plots are colored by cell identity annotations (top) and batch labels (bottom). b, Metrics for evaluating the mapping performance for benchmarking methods.

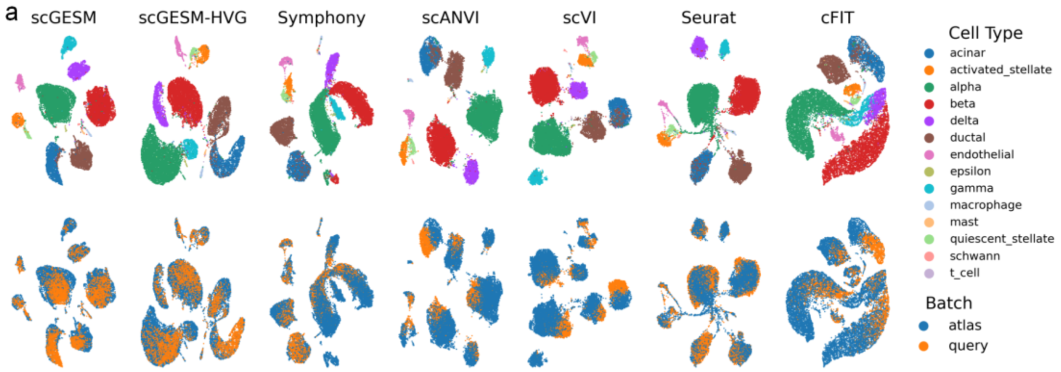

b

Method	Batch correction: PCR batch	Batch correction: Batch ASW	Batch correction: Graph iLISI	Batch correction: Graph connectivity	Batch correction: kBET	Bio-conservation: NMI cluster/label	Bio-conservation: ARI cluster/label	Bio-conservation: Cell type ASW	Bio-conservation: Isolated label F1	Bio-conservation: Isolated label silhouette	Bio-conservation: Graph cLISI	Aggregate score: Batch correction	Aggregate score: Bio-conservation	Aggregate score: Total
scGESM-HVG	0.95	0.80	0.31	0.96	0.62	0.91	0.94	0.75	0.89	0.73	1.00	0.73	0.87	0.81
scGESM	0.93	0.84	0.30	0.98	0.61	0.94	0.96	0.65	0.85	0.72	1.00	0.73	0.85	0.81
Seurat	0.88	0.82	0.15	0.98	0.59	0.89	0.91	0.69	0.72	0.69	1.00	0.68	0.82	0.76
Symphony	0.74	0.85	0.35	0.96	0.44	0.87	0.90	0.64	0.71	0.64	1.00	0.67	0.79	0.74
scANVI	0.28	0.81	0.11	0.99	0.38	0.92	0.95	0.63	0.88	0.67	1.00	0.51	0.84	0.71
scVI	0.14	0.83	0.09	0.98	0.46	0.89	0.94	0.60	0.89	0.66	1.00	0.50	0.83	0.70
cFIT	0.41	0.76	0.15	0.94	0.41	0.87	0.92	0.66	0.68	0.63	1.00	0.53	0.79	0.69

Fig.S30 Performance of benchmarking methods for mapping the inDrop3 batch onto the reference constructed using other batches in the human pancreas data. a, Visualization based on the mapping results of benchmarking methods. UMAP plots are colored by cell identity annotations (top) and batch labels (bottom). b, Metrics for evaluating the mapping performance for benchmarking methods.

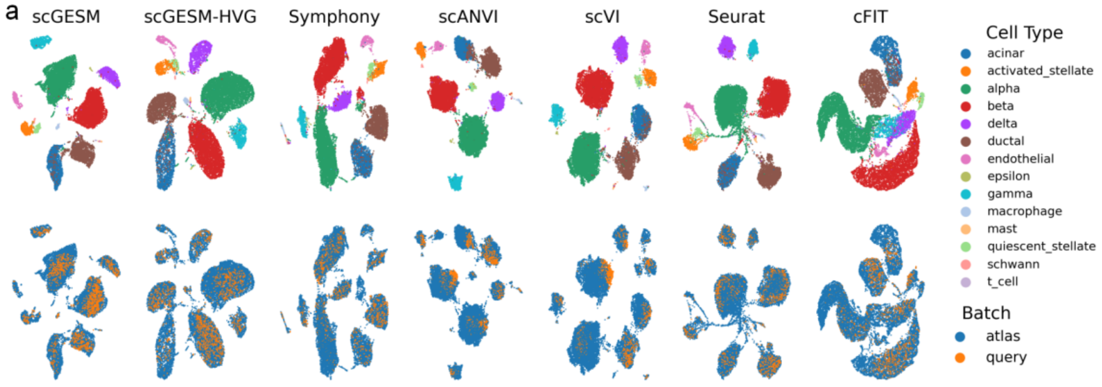

b

Method	Batch correction: PCR batch	Batch correction: Batch ASW	Batch correction: Graph iLISI	Batch correction: Graph connectivity	Batch correction: kBET	Bio-conservation: NMI cluster/label	Bio-conservation: ARI cluster/label	Bio-conservation: Cell type ASW	Bio-conservation: Isolated label F1	Bio-conservation: Isolated label silhouette	Bio-conservation: Graph cLISI	Aggregate score: Batch correction	Aggregate score: Bio-conservation	Aggregate score: Total
scGESM	0.93	0.80	0.10	1.00	0.84	0.94	0.96	0.67	0.92	0.72	1.00	0.73	0.87	0.82
scGESM-HVG	0.94	0.81	0.09	0.97	0.78	0.91	0.95	0.72	0.80	0.69	1.00	0.72	0.84	0.79
Seurat	0.91	0.84	0.06	0.98	0.91	0.89	0.91	0.69	0.72	0.69	1.00	0.74	0.82	0.79
Symphony	0.80	0.86	0.10	0.98	0.80	0.87	0.90	0.66	0.70	0.64	1.00	0.71	0.80	0.76
scANVI	0.00	0.87	0.05	0.98	0.69	0.93	0.96	0.64	0.89	0.69	1.00	0.52	0.85	0.72
scVI	0.00	0.87	0.04	0.99	0.71	0.91	0.95	0.60	0.90	0.66	1.00	0.52	0.84	0.71
cFIT	0.31	0.78	0.09	0.93	0.67	0.82	0.74	0.65	0.72	0.62	1.00	0.56	0.76	0.68

Fig.S31 Performance of benchmarking methods for mapping the inDrop4 batch onto the reference constructed using other batches in the human pancreas data. a, Visualization based on the mapping results of benchmarking methods. UMAP plots are colored by cell identity annotations (top) and batch labels (bottom). b, Metrics for evaluating the mapping performance for benchmarking methods.

a

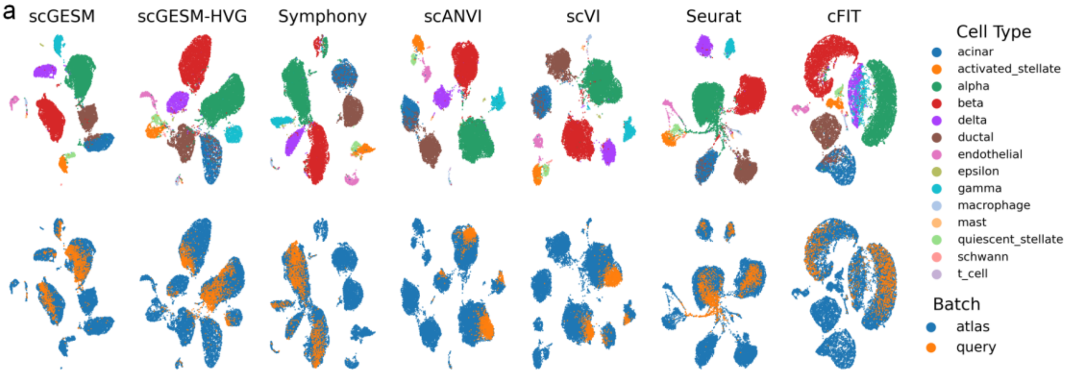

b

Method	Batch correction: PCR batch	Batch correction: Batch ASW	Batch correction: Graph iLISI	Batch correction: Graph connectivity	Batch correction: kBET	Bio-conservation: NMI cluster/label	Bio-conservation: ARI cluster/label	Bio-conservation: Cell type ASW	Bio-conservation: Isolated label F1	Bio-conservation: Isolated label silhouette	Bio-conservation: Graph cLISI	Aggregate score: Batch correction	Aggregate score: Bio-conservation	Aggregate score: Total
scGESM	0.84	0.75	0.02	1.00	0.45	0.93	0.96	0.66	0.89	0.76	1.00	0.61	0.87	0.76
Seurat	0.79	0.90	0.17	0.98	0.42	0.89	0.91	0.69	0.62	0.70	1.00	0.65	0.80	0.74
scGESM-HVG	0.85	0.61	0.01	0.98	0.34	0.90	0.94	0.70	0.78	0.68	1.00	0.56	0.84	0.72
cFIT	0.66	0.85	0.06	0.94	0.62	0.88	0.92	0.65	0.63	0.62	1.00	0.63	0.79	0.72
Symphony	0.55	0.90	0.07	0.96	0.69	0.87	0.90	0.65	0.60	0.64	1.00	0.63	0.77	0.72
scANVI	0.12	0.86	0.00	0.99	0.34	0.92	0.95	0.63	0.86	0.70	1.00	0.46	0.84	0.69
scVI	0.04	0.86	0.00	0.98	0.31	0.91	0.95	0.60	0.78	0.67	1.00	0.44	0.82	0.67

Fig.S32 Performance of benchmarking methods for mapping the Smarter batch onto the reference constructed using other batches in the human pancreas data. a, Visualization based on the mapping results of benchmarking methods. UMAP plots are colored by cell identity annotations (top) and batch labels (bottom). b, Metrics for evaluating the mapping performance for benchmarking methods.

a

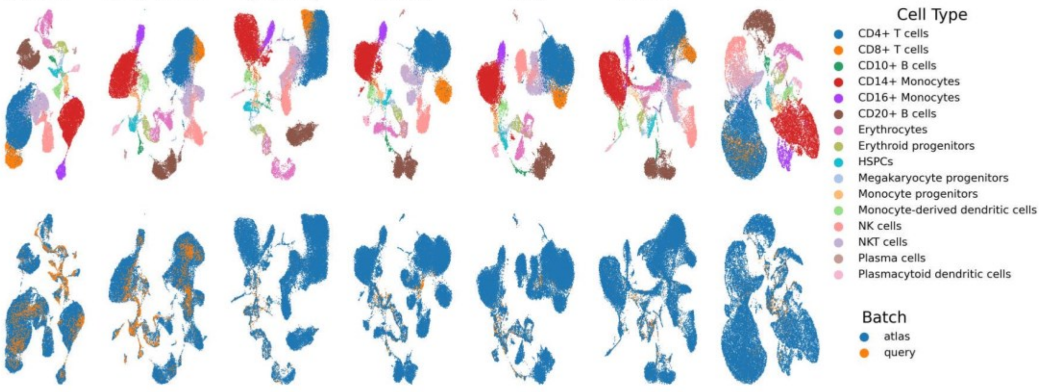

b

Method	Batch correction: PCR batch	Batch correction: Batch ASW	Batch correction: Graph iLISI	Batch correction: Graph connectivity	Batch correction: kBET	Bio-conservation: NMI cluster/label	Bio-conservation: ARI cluster/label	Bio-conservation: Cell type ASW	Bio-conservation: Isolated label F1	Bio-conservation: Isolated label silhouette	Bio-conservation: Graph cLISI	Aggregate score: Batch correction	Aggregate score: Bio-conservation	Aggregate score: Total
scGESM	0.66	0.86	0.03	0.99	0.53	0.87	0.83	0.62	0.88	0.67	1.00	0.61	0.81	0.73
Seurat	0.91	0.84	0.16	0.97	0.59	0.77	0.73	0.57	0.78	0.59	0.99	0.69	0.74	0.72
scGESM-HVG	0.80	0.87	0.05	0.98	0.54	0.80	0.74	0.60	0.85	0.64	1.00	0.65	0.77	0.72
scANVI	0.25	0.89	0.05	0.97	0.55	0.88	0.92	0.61	0.89	0.64	1.00	0.54	0.82	0.71
scVI	0.39	0.90	0.05	0.97	0.57	0.80	0.82	0.56	0.83	0.60	0.99	0.57	0.77	0.69
Symphony	0.39	0.88	0.04	0.97	0.70	0.78	0.73	0.59	0.76	0.62	1.00	0.60	0.75	0.69
cFIT	0.61	0.79	0.04	0.98	0.47	0.76	0.69	0.57	0.74	0.61	0.99	0.58	0.73	0.67

Fig.S33 Performance of benchmarking methods for mapping the Oetjen_A batch onto the reference constructed using other batches in the human PBMC data. a, Visualization based on the mapping results of benchmarking methods. UMAP plots are colored by cell identity annotations (top) and batch labels (bottom). b, Metrics for evaluating the mapping performance for benchmarking methods.

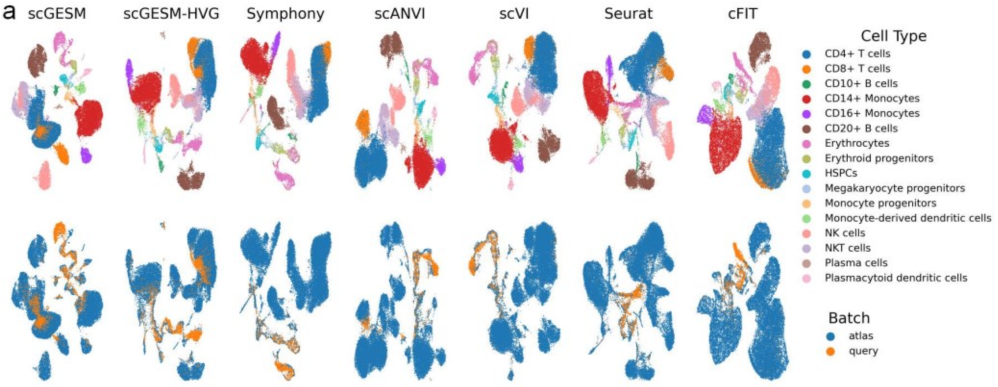

b

Method	Batch correction					Bio-conservation						Aggregate score		
	PCR batch	Batch ASW	Graph iLISI	Graph connectivity	kBET	NMI cluster/label	ARI cluster/label	Cell type ASW	Isolated label F1	Isolated label silhouette	Graph cLISI	Batch correction	Bio-conservation	Total
Seurat	0.97	0.83	0.09	0.97	0.67	0.77	0.73	0.57	0.84	0.59	0.99	0.71	0.75	0.73
scGESM-HVG	0.85	0.80	0.01	0.98	0.49	0.82	0.78	0.60	0.85	0.59	1.00	0.63	0.77	0.71
scGESM	0.66	0.79	0.01	0.99	0.45	0.87	0.88	0.62	0.85	0.58	1.00	0.58	0.80	0.71
scANVI	0.34	0.86	0.01	0.98	0.58	0.88	0.92	0.60	0.85	0.59	1.00	0.55	0.81	0.71
cFIT	0.74	0.79	0.03	0.98	0.52	0.78	0.74	0.58	0.78	0.59	1.00	0.61	0.75	0.69
scVI	0.41	0.87	0.03	0.98	0.59	0.81	0.83	0.55	0.84	0.58	0.99	0.58	0.77	0.69
Symphony	0.00	0.80	0.02	0.97	0.64	0.77	0.74	0.60	0.81	0.59	1.00	0.49	0.75	0.64

Fig.S34 Performance of benchmarking methods for mapping the Oetjen_P batch onto the reference constructed using other batches in the human PBMC data. a, Visualization based on the mapping results of benchmarking methods. UMAP plots are colored by cell identity annotations (top) and batch labels (bottom). b, Metrics for evaluating the mapping performance for benchmarking methods.

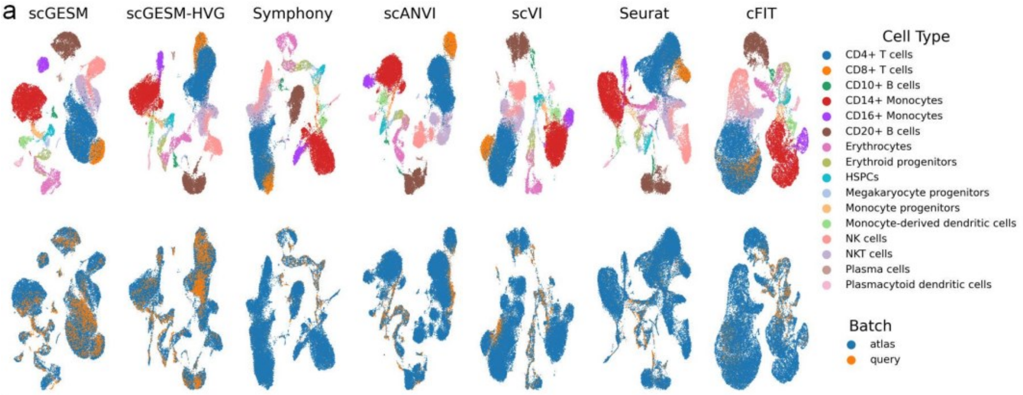

b

Method	Batch correction					Bio-conservation						Aggregate score		
	PCR batch	Batch ASW	Graph iLISI	Graph connectivity	kBET	NMI cluster/label	ARI cluster/label	Cell type ASW	Isolated label F1	Isolated label silhouette	Graph cLISI	Batch correction	Bio-conservation	Total
scGESM	0.90	0.84	0.09	0.98	0.52	0.85	0.71	0.62	0.88	0.67	1.00	0.67	0.79	0.74
scANVI	0.60	0.89	0.11	0.98	0.56	0.87	0.90	0.60	0.86	0.63	1.00	0.63	0.81	0.74
scGESM-HVG	0.92	0.86	0.08	0.97	0.48	0.81	0.77	0.60	0.85	0.64	1.00	0.66	0.78	0.73
Seurat	0.86	0.86	0.16	0.97	0.65	0.77	0.73	0.57	0.78	0.59	0.99	0.70	0.74	0.72
Symphony	0.84	0.89	0.10	0.98	0.64	0.76	0.60	0.59	0.74	0.62	1.00	0.69	0.72	0.71
cFIT	0.52	0.82	0.11	0.98	0.45	0.78	0.73	0.58	0.75	0.61	1.00	0.58	0.74	0.67
scVI	0.28	0.89	0.09	0.99	0.52	0.76	0.64	0.55	0.82	0.59	0.99	0.55	0.73	0.66

Fig.S35 Performance of benchmarking methods for mapping the Oetjen_U batch onto the reference constructed using other batches in the human PBMC data. a, Visualization based on the mapping results of benchmarking methods. UMAP plots are colored by cell identity annotations (top) and batch labels (bottom). b, Metrics for evaluating the mapping performance for benchmarking methods.

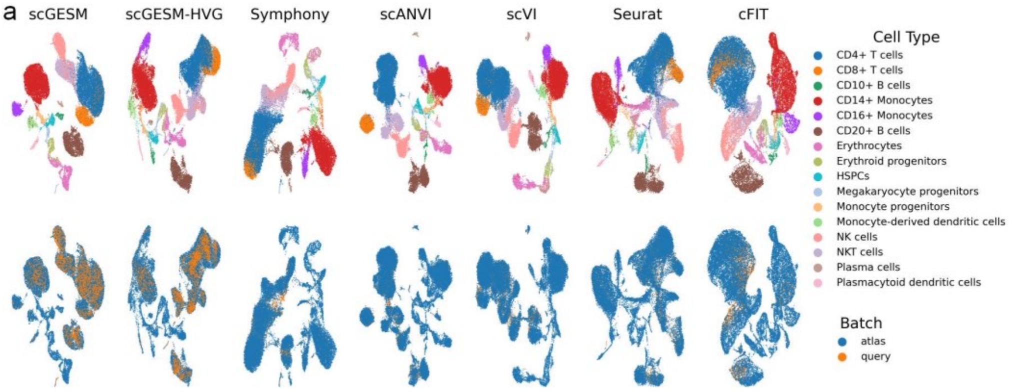

b

Method	Batch correction: PCR batch	Batch correction: Batch ASW	Batch correction: Graph iLISI	Batch correction: Graph connectivity	Batch correction: kBET	Bio-conservation: NMI cluster/label	Bio-conservation: ARI cluster/label	Bio-conservation: Cell type ASW	Bio-conservation: Isolated label F1	Bio-conservation: Isolated label silhouette	Bio-conservation: Graph cLISI	Aggregate score: Batch correction	Aggregate score: Bio-conservation	Aggregate score: Total
scGESM	0.89	0.86	0.10	0.98	0.65	0.89	0.92	0.61	0.93	0.70	1.00	0.70	0.84	0.78
scANVI	0.77	0.89	0.12	0.98	0.60	0.89	0.93	0.62	0.87	0.64	1.00	0.67	0.82	0.76
scGESM-HVG	0.94	0.85	0.09	0.97	0.60	0.78	0.65	0.61	0.85	0.67	1.00	0.69	0.76	0.73
Seurat	0.84	0.85	0.14	0.97	0.73	0.77	0.73	0.57	0.73	0.58	0.99	0.71	0.73	0.72
scVI	0.66	0.88	0.09	0.98	0.57	0.81	0.82	0.55	0.81	0.62	0.99	0.64	0.77	0.71
Symphony	0.82	0.82	0.18	0.98	0.59	0.77	0.61	0.59	0.78	0.66	1.00	0.68	0.74	0.71
cFIT	0.49	0.80	0.07	0.98	0.52	0.80	0.74	0.58	0.74	0.61	1.00	0.57	0.74	0.67

Fig.S36 Performance of benchmarking methods for mapping the Freytag batch onto the reference constructed using other batches in the human PBMC data. a, Visualization based on the mapping results of benchmarking methods. UMAP plots are colored by cell identity annotations (top) and batch labels (bottom). b, Metrics for evaluating the mapping performance for benchmarking methods.

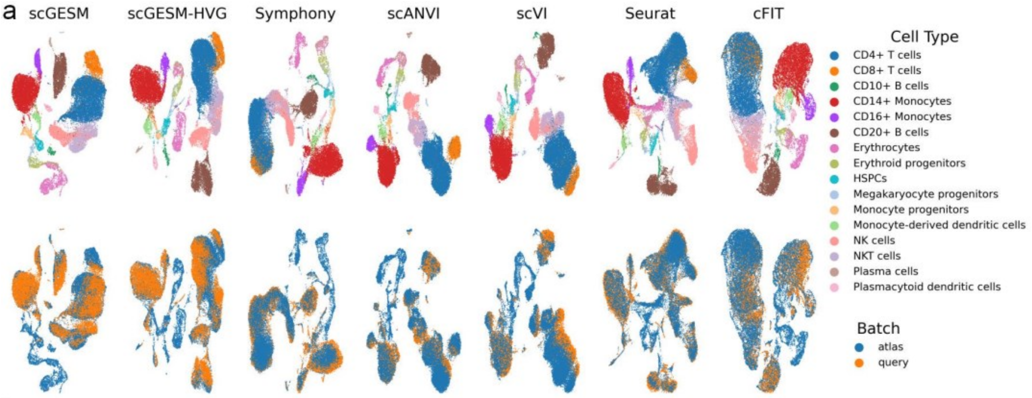

b

Method	Batch correction: PCR batch	Batch correction: Batch ASW	Batch correction: Graph iLISI	Batch correction: Graph connectivity	Batch correction: kBET	Bio-conservation: NMI cluster/label	Bio-conservation: ARI cluster/label	Bio-conservation: Cell type ASW	Bio-conservation: Isolated label F1	Bio-conservation: Isolated label silhouette	Bio-conservation: Graph cLISI	Aggregate score: Batch correction	Aggregate score: Bio-conservation	Aggregate score: Total
scGESM	0.90	0.81	0.45	0.98	0.40	0.89	0.92	0.62	0.89	0.68	1.00	0.71	0.83	0.78
scANVI	0.85	0.86	0.42	0.98	0.41	0.87	0.91	0.62	0.86	0.62	1.00	0.70	0.81	0.77
scGESM-HVG	0.88	0.80	0.49	0.97	0.39	0.80	0.67	0.61	0.82	0.67	1.00	0.71	0.76	0.74
Symphony	0.82	0.84	0.43	0.97	0.55	0.77	0.71	0.59	0.79	0.63	0.99	0.72	0.75	0.74
cFIT	0.81	0.85	0.55	0.98	0.49	0.77	0.60	0.57	0.75	0.57	0.99	0.74	0.71	0.72
Seurat	0.84	0.80	0.37	0.97	0.51	0.77	0.73	0.57	0.63	0.53	0.99	0.70	0.71	0.70
scVI	0.68	0.84	0.26	0.98	0.35	0.81	0.82	0.56	0.76	0.59	0.99	0.62	0.76	0.70

Fig.S37 Performance of benchmarking methods for mapping the 10X batch onto the reference constructed using other batches in the human PBMC data. a, Visualization based on the mapping results of benchmarking methods. UMAP plots are colored by cell identity annotations (top) and batch labels (bottom). b, Metrics for evaluating the mapping performance for benchmarking methods.

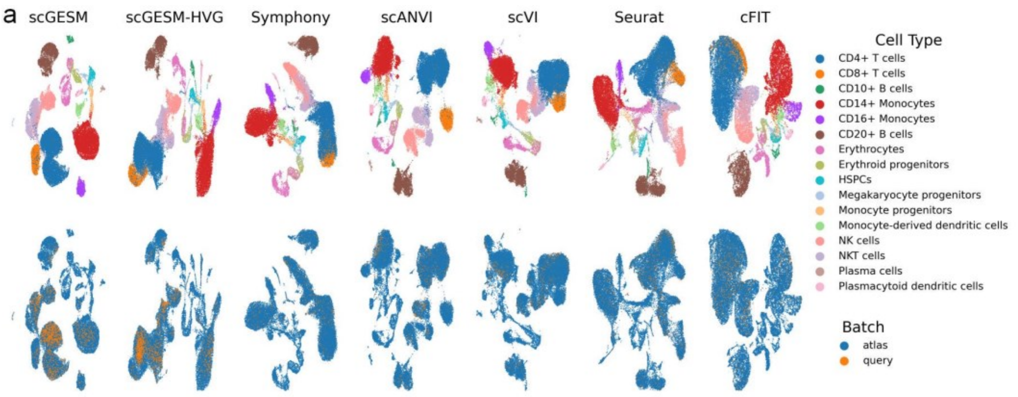

b

Method	Batch correction: PCR batch	Batch ASW	Graph iLISI	Graph connectivity	kBET	Bio-conservation: NMI cluster/label	ARI cluster/label	Cell type ASW	Isolated label F1	Isolated label silhouette	Graph cLISI	Aggregate score: Batch correction	Bio-conservation	Total
scGESM	0.88	0.83	0.03	0.98	0.82	0.86	0.72	0.62	0.93	0.70	1.00	0.71	0.81	0.77
scANVI	0.49	0.88	0.03	0.98	0.78	0.89	0.92	0.62	0.87	0.66	1.00	0.63	0.83	0.75
scGESM-HVG	0.86	0.83	0.03	0.98	0.78	0.79	0.67	0.62	0.85	0.68	1.00	0.70	0.77	0.74
Symphony	0.77	0.83	0.08	0.98	0.74	0.79	0.74	0.60	0.78	0.67	1.00	0.68	0.76	0.73
Seurat	0.75	0.84	0.03	0.97	0.91	0.77	0.73	0.57	0.73	0.60	0.99	0.70	0.73	0.72
cFIT	0.70	0.83	0.04	0.98	0.83	0.79	0.73	0.58	0.76	0.64	1.00	0.67	0.75	0.72
scVI	0.51	0.88	0.03	0.98	0.74	0.81	0.82	0.56	0.84	0.63	0.99	0.63	0.78	0.72

Fig.S38 Performance of benchmarking methods for mapping the Sun_sample1_CS batch onto the reference constructed using other batches in the human PBMC data. a, Visualization based on the mapping results of benchmarking methods. UMAP plots are colored by cell identity annotations (top) and batch labels (bottom). b, Metrics for evaluating the mapping performance for benchmarking methods.

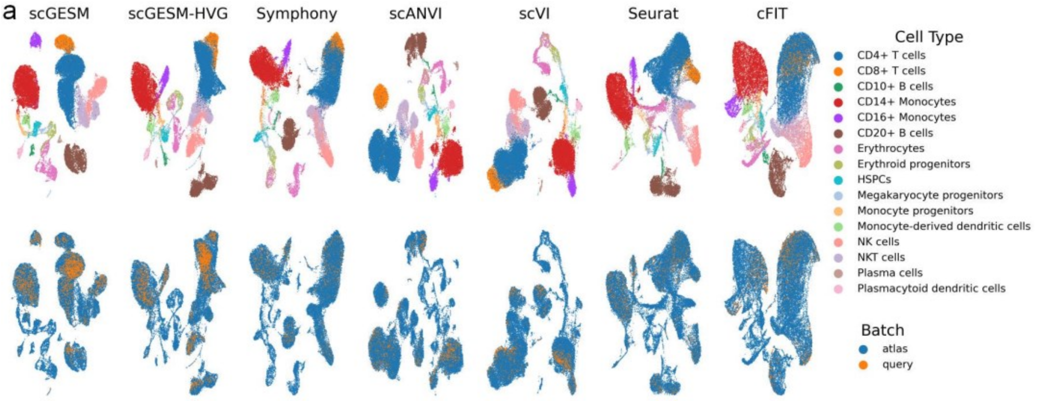

b

Method	Batch correction: PCR batch	Batch ASW	Graph iLISI	Graph connectivity	kBET	Bio-conservation: NMI cluster/label	ARI cluster/label	Cell type ASW	Isolated label F1	Isolated label silhouette	Graph cLISI	Aggregate score: Batch correction	Bio-conservation	Total
scGESM	0.95	0.84	0.06	0.99	0.77	0.86	0.72	0.62	0.94	0.71	1.00	0.72	0.81	0.77
scGESM-HVG	0.93	0.86	0.06	0.97	0.77	0.82	0.78	0.61	0.86	0.67	1.00	0.72	0.79	0.76
scANVI	0.72	0.88	0.04	0.98	0.68	0.87	0.91	0.61	0.88	0.65	1.00	0.66	0.82	0.76
Seurat	0.95	0.82	0.17	0.97	0.63	0.77	0.73	0.57	0.73	0.58	0.99	0.71	0.73	0.72
Symphony	0.83	0.81	0.13	0.98	0.63	0.77	0.61	0.59	0.77	0.65	1.00	0.68	0.73	0.71
scVI	0.56	0.88	0.05	0.98	0.69	0.77	0.72	0.56	0.82	0.63	0.99	0.63	0.75	0.70
cFIT	0.38	0.81	0.04	0.98	0.69	0.75	0.61	0.56	0.79	0.62	0.99	0.58	0.72	0.66

Fig.S39 Performance of benchmarking methods for mapping the Sun_sample1_KC batch onto the reference constructed using other batches in the human PBMC data. a, Visualization based on the mapping results of benchmarking methods. UMAP plots are colored by cell identity annotations (top) and batch labels (bottom). b, Metrics for evaluating the mapping performance for benchmarking methods.

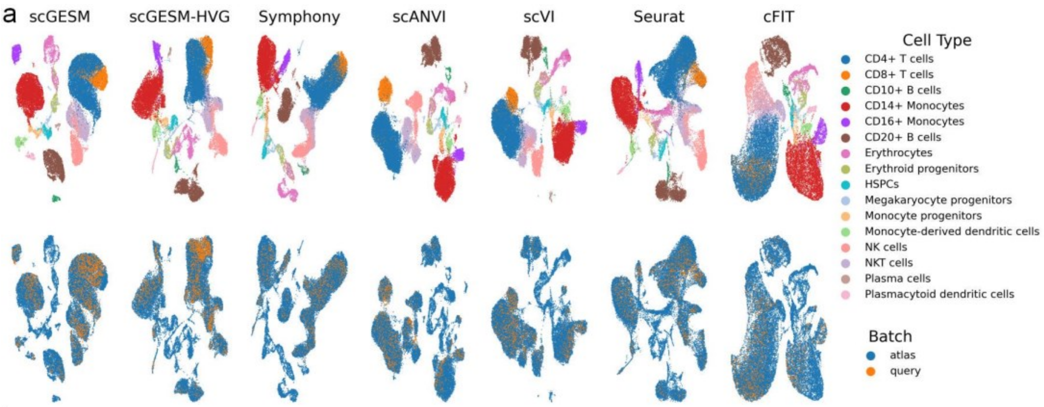

b

Method	Batch correction: PCR batch	Batch correction: Batch ASW	Batch correction: Graph iLISI	Batch correction: Graph connectivity	Batch correction: kBET	Bio-conservation: NMI cluster/label	Bio-conservation: ARI cluster/label	Bio-conservation: Cell type ASW	Bio-conservation: Isolated label F1	Bio-conservation: Isolated label silhouette	Bio-conservation: Graph cLISI	Aggregate score: Batch correction	Aggregate score: Bio-conservation	Aggregate score: Total
scGESM	0.87	0.87	0.06	0.98	0.67	0.91	0.93	0.62	0.87	0.66	1.00	0.69	0.83	0.77
scANVI	0.69	0.91	0.07	0.99	0.66	0.88	0.92	0.61	0.81	0.63	1.00	0.66	0.81	0.75
scGESM-HVG	0.89	0.88	0.06	0.98	0.61	0.81	0.76	0.60	0.78	0.61	1.00	0.68	0.76	0.73
scVI	0.77	0.91	0.09	0.99	0.68	0.81	0.82	0.55	0.77	0.60	0.99	0.69	0.76	0.73
Symphony	0.78	0.83	0.13	0.96	0.59	0.78	0.74	0.59	0.72	0.61	1.00	0.66	0.74	0.71
Seurat	0.83	0.85	0.18	0.97	0.55	0.77	0.73	0.57	0.65	0.53	0.99	0.68	0.71	0.70
cFIT	0.36	0.83	0.06	0.98	0.60	0.76	0.62	0.56	0.71	0.58	0.99	0.57	0.70	0.65

Fig.S40 Performance of benchmarking methods for mapping the Sun_sample1_TB batch onto the reference constructed using other batches in the human PBMC data. a, Visualization based on the mapping results of benchmarking methods. UMAP plots are colored by cell identity annotations (top) and batch labels (bottom). b, Metrics for evaluating the mapping performance for benchmarking methods.

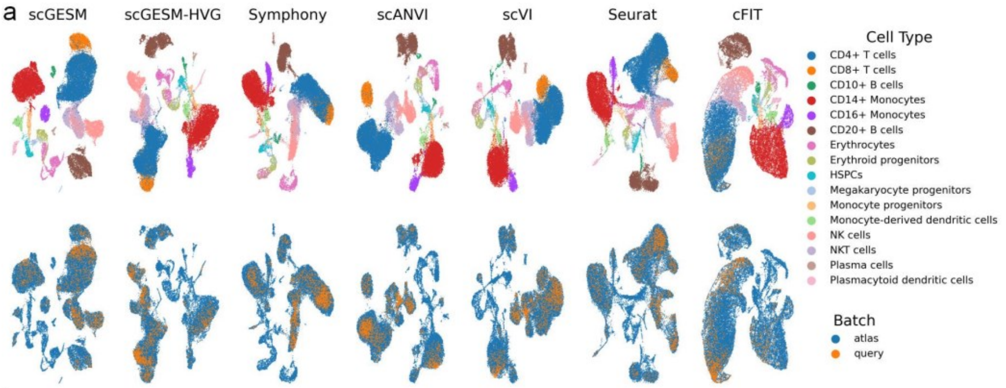

b

Method	Batch correction: PCR batch	Batch correction: Batch ASW	Batch correction: Graph iLISI	Batch correction: Graph connectivity	Batch correction: kBET	Bio-conservation: NMI cluster/label	Bio-conservation: ARI cluster/label	Bio-conservation: Cell type ASW	Bio-conservation: Isolated label F1	Bio-conservation: Isolated label silhouette	Bio-conservation: Graph cLISI	Aggregate score: Batch correction	Aggregate score: Bio-conservation	Aggregate score: Total
scGESM	0.88	0.90	0.06	0.98	0.76	0.90	0.93	0.63	0.89	0.65	1.00	0.71	0.83	0.79
scANVI	0.86	0.92	0.08	0.98	0.75	0.86	0.90	0.61	0.74	0.61	1.00	0.72	0.79	0.76
scVI	0.92	0.92	0.08	0.98	0.74	0.82	0.83	0.55	0.76	0.60	0.99	0.73	0.76	0.75
scGESM-HVG	0.89	0.91	0.06	0.99	0.71	0.79	0.75	0.61	0.78	0.60	1.00	0.71	0.76	0.74
Symphony	0.78	0.84	0.13	0.97	0.73	0.77	0.72	0.59	0.73	0.61	1.00	0.69	0.74	0.72
Seurat	0.83	0.92	0.07	0.97	0.86	0.77	0.73	0.57	0.65	0.53	0.99	0.73	0.71	0.72
cFIT	0.52	0.86	0.05	0.98	0.69	0.76	0.61	0.57	0.72	0.57	0.99	0.62	0.70	0.67

Fig.S41 Performance of benchmarking methods for mapping the Sun_sample1_TC batch onto the reference constructed using other batches in the human PBMC data. a, Visualization based on the mapping results of benchmarking methods. UMAP plots are colored by cell identity annotations (top) and batch labels (bottom). b, Metrics for evaluating the mapping performance for benchmarking methods.

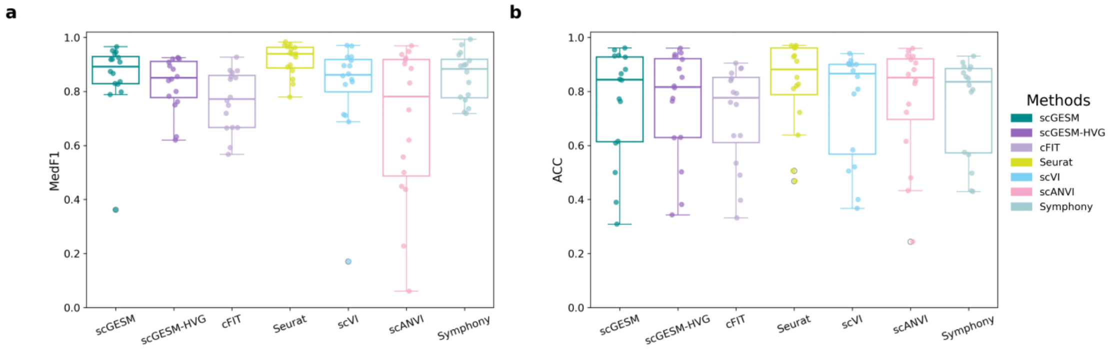

Fig.S42 Performance of benchmarking methods for query data annotation on the human lung data. a, The median cell-type F1-scores. b, The accuracy of the predicted cell type labels. Each point in the box plot represents a batch.

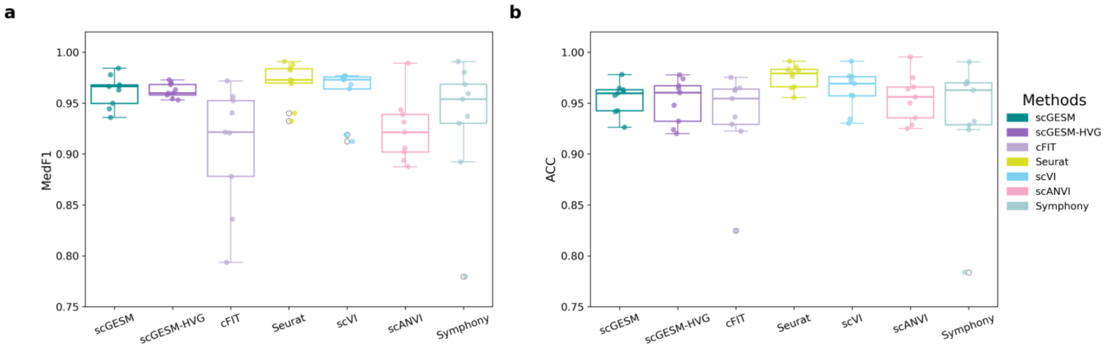

Fig.S43 Performance of benchmarking methods for query data annotation on the pancreas data. a, The median cell-type F1-scores. b, The accuracy of the predicted cell type labels. Each point in the box plot represents a batch.

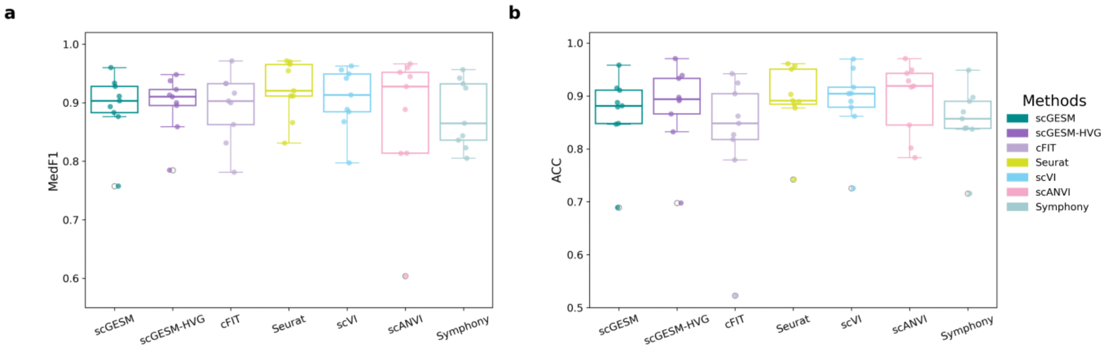

Fig.S44 Performance of benchmarking methods for query data annotation on the PBMC data. a, The median cell-type F1-scores. b, The accuracy of the predicted cell type labels. Each point in the box plot represents a batch.

Method	Batch correction: PCR batch	Batch correction: Batch ASW	Batch correction: Graph iLISI	Batch correction: Graph connectivity	Batch correction: kBET	Bio-conservation: NMI cluster/label	Bio-conservation: ARI cluster/label	Bio-conservation: Cell type ASW	Bio-conservation: Isolated label F1	Bio-conservation: Isolated label silhouette	Bio-conservation: Graph cLISI	Aggregate score: Batch correction	Aggregate score: Bio-conservation	Aggregate score: Total
scGESI	0.86	0.81	0.29	0.99	0.50	0.96	0.98	0.65	0.10	0.76	1.00	0.69	0.74	0.72
Scanorama	0.19	0.81	0.02	0.98	0.27	0.75	0.52	0.61	0.56	0.75	1.00	0.45	0.70	0.60
CarDEC	0.00	0.85	0.13	0.94	0.31	0.89	0.93	0.55	0.07	0.69	1.00	0.45	0.69	0.59
scGESI-HVG	0.88	0.77	0.30	0.98	0.49	0.91	0.94	0.73	0.10	0.59	1.00	0.68	0.71	0.70
CarDEC-HVG	0.62	0.79	0.21	0.97	0.40	0.91	0.95	0.70	0.19	0.77	1.00	0.60	0.75	0.69
Scanorama-HVG	0.46	0.79	0.12	0.92	0.24	0.79	0.63	0.64	0.13	0.71	1.00	0.51	0.65	0.59
scGESI-LVG	0.86	0.80	0.29	0.99	0.50	0.95	0.97	0.65	0.19	0.77	1.00	0.69	0.75	0.73
CarDEC-LVG	0.00	0.85	0.14	0.91	0.30	0.84	0.90	0.53	0.03	0.60	1.00	0.44	0.65	0.57
Scanorama-LVG	0.00	0.77	0.09	0.74	0.15	0.64	0.42	0.52	0.15	0.71	1.00	0.35	0.57	0.48

Fig.S45 Performance of scGESI, Scanorama and CarDEC for whole genome denoising analysis on the pancreas data. Integration performance metrics based on denoised gene expression data for the entire gene set, HVGs and LVGs.

a

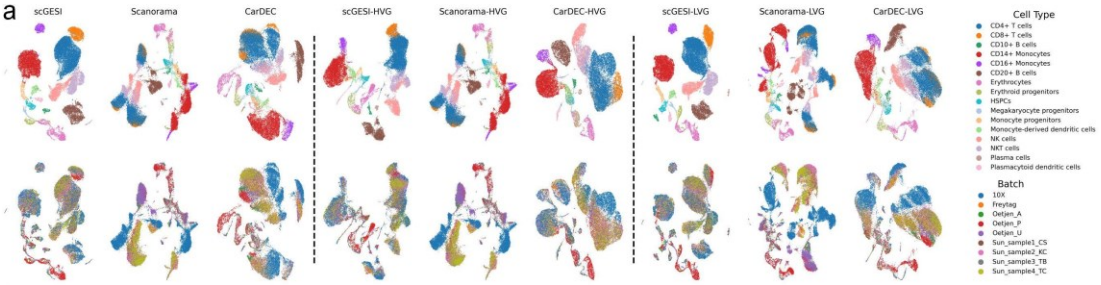

b

Method	Batch correction: PCR batch	Batch correction: Batch ASW	Batch correction: Graph iLISI	Batch correction: Graph connectivity	Batch correction: kBET	Bio-conservation: NMI cluster/label	Bio-conservation: ARI cluster/label	Bio-conservation: Cell type ASW	Bio-conservation: Isolated label F1	Bio-conservation: Isolated label silhouette	Bio-conservation: Graph cLISI	Aggregate score: Batch correction	Aggregate score: Bio-conservation	Aggregate score: Total
scGESI	0.80	0.85	0.24	0.98	0.22	0.91	0.93	0.62	0.91	0.67	1.00	0.62	0.84	0.75
CarDEC	0.26	0.84	0.18	0.97	0.17	0.74	0.62	0.57	0.79	0.66	1.00	0.49	0.73	0.63
Scanorama	0.43	0.88	0.09	0.99	0.14	0.71	0.45	0.58	0.85	0.64	1.00	0.51	0.71	0.63
scGESI-HVG	0.89	0.87	0.25	0.98	0.20	0.83	0.85	0.60	0.82	0.63	1.00	0.64	0.79	0.73
CarDEC-HVG	0.66	0.80	0.22	0.96	0.26	0.77	0.66	0.65	0.69	0.69	1.00	0.58	0.74	0.68
Scanorama-HVG	0.50	0.92	0.19	0.97	0.28	0.66	0.43	0.54	0.75	0.59	0.99	0.57	0.66	0.62
scGESI-LVG	0.79	0.85	0.25	0.98	0.24	0.94	0.96	0.62	0.93	0.68	1.00	0.62	0.85	0.76
CarDEC-LVG	0.27	0.85	0.18	0.97	0.18	0.74	0.63	0.55	0.79	0.64	0.99	0.49	0.72	0.63
Scanorama-LVG	0.36	0.88	0.11	0.93	0.23	0.68	0.42	0.57	0.84	0.64	1.00	0.50	0.69	0.62

Fig.S46 Performance of scGESI, Scanorama and CarDEC for whole genome denoising analysis on the PBMC data. a, Visualization based on the denoised gene expression data for the entire gene set, HVGs and LVGs. UMAP plots are colored by cell identity annotations (top) and batch labels (bottom). b, Integration performance metrics based on denoised gene expression data for the entire gene set, HVGs and LVGs.

a

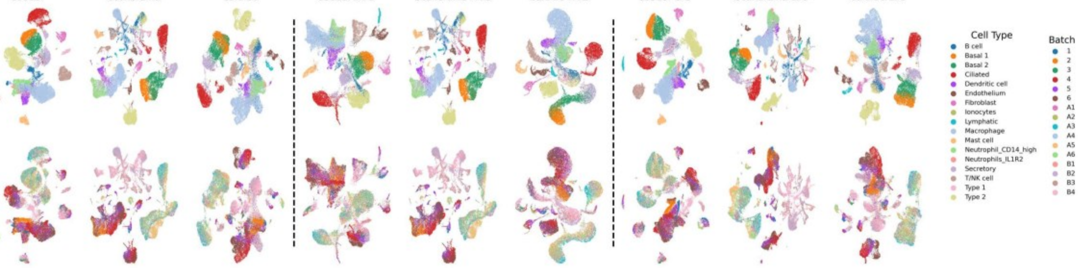

b

Method	Batch correction					Bio-conservation						Aggregate score		
	PCR batch	Batch ASW	Graph iLISI	Graph connectivity	kBET	NMI cluster/label	ARI cluster/label	Cell type ASW	Isolated label F1	Isolated label silhouette	Graph cLISI	Batch correction	Bio-conservation	Total
scGESI	0.67	0.84	0.13	0.99	0.38	0.84	0.76	0.62	0.99	0.76	1.00	0.60	0.83	0.74
Scanorama	0.31	0.87	0.03	0.99	0.26	0.75	0.52	0.62	0.92	0.66	1.00	0.49	0.74	0.64
CarDEC	0.26	0.90	0.10	0.97	0.34	0.71	0.50	0.57	0.64	0.65	0.99	0.51	0.68	0.61
scGESI-HVG	0.84	0.84	0.14	0.96	0.38	0.73	0.57	0.59	0.86	0.69	0.99	0.63	0.74	0.70
Scanorama-HVG	0.41	0.83	0.10	0.96	0.31	0.72	0.48	0.60	0.88	0.74	1.00	0.52	0.74	0.65
CarDEC-HVG	0.47	0.81	0.11	0.96	0.37	0.72	0.57	0.62	0.58	0.74	0.99	0.54	0.70	0.64
scGESI-LVG	0.65	0.83	0.13	0.99	0.40	0.85	0.76	0.63	0.99	0.77	1.00	0.60	0.83	0.74
Scanorama-LVG	0.13	0.86	0.09	0.78	0.32	0.69	0.45	0.52	0.83	0.52	1.00	0.44	0.67	0.57
CarDEC-LVG	0.28	0.91	0.10	0.96	0.34	0.70	0.49	0.56	0.29	0.63	0.99	0.52	0.61	0.57

Fig.S47 Performance of scGESI, Scanorama and CarDEC for whole genome denoising analysis on the lung data. a, Visualization based on the denoised gene expression data for the entire gene set, HVGs and LVGs. UMAP plots are colored by cell identity annotations (top) and batch labels (bottom). b, Integration performance metrics based on denoised gene expression data for the entire gene set, HVGs and LVGs.

a

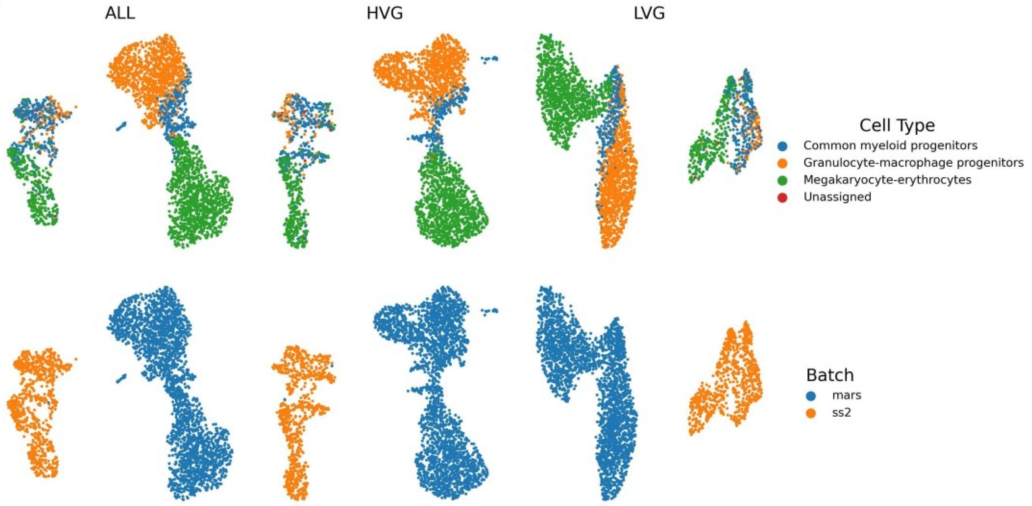

b

Method	Batch correction					Bio-conservation						Aggregate score		
	PCR batch	Batch ASW	Graph iLISI	Graph connectivity	kBET	NMI cluster/label	ARI cluster/label	Cell type ASW	Isolated label F1	Isolated label silhouette	Graph cLISI	Batch correction	Bio-conservation	Total
HVG	0.00	0.58	0.00	0.80	0.01	0.50	0.56	0.61	0.04	0.48	0.98	0.28	0.53	0.43
ALL	0.00	0.53	0.00	0.71	0.00	0.51	0.54	0.57	0.03	0.50	0.98	0.25	0.52	0.41
LVG	0.00	0.46	0.00	0.58	0.25	0.48	0.48	0.53	0.05	0.56	0.98	0.26	0.51	0.41

Fig.Fig.S48 Analysis on the raw human HSC data. a, Visualizations using all genes, HVGs and LVGs with the Scanpy Louvain workflow. UMAP plots are colored by cell identity annotations (top) and batch labels (bottom). b, Metrics for evaluating the integration performance.

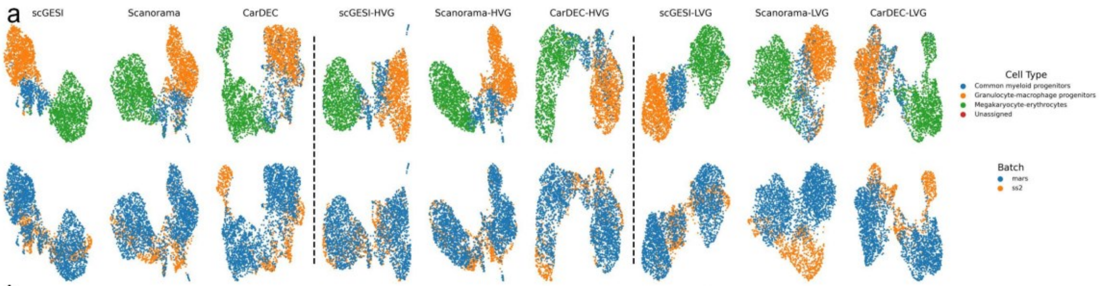

b

Method	Batch correction: PCR batch	Batch correction: Batch ASW	Batch correction: Graph iLISI	Batch correction: Graph connectivity	Batch correction: kBET	Bio-conservation: NMI cluster/label	Bio-conservation: ARI cluster/label	Bio-conservation: Cell type ASW	Bio-conservation: Isolated label F1	Bio-conservation: Isolated label silhouette	Bio-conservation: Graph cLISI	Aggregate score: Batch correction	Aggregate score: Bio-conservation	Aggregate score: Total
**scGESI**	0.77	0.88	0.34	0.78	0.48	0.76	0.85	0.62	0.04	0.38	0.99	0.65	0.60	0.62
**CarDEC**	0.56	0.75	0.06	0.76	0.20	0.60	0.68	0.59	0.03	0.43	0.97	0.47	0.55	0.52
**Scanorama**	0.24	0.57	0.00	0.77	0.00	0.50	0.56	0.62	0.03	0.43	0.98	0.32	0.52	0.44
**scGESI-HVG**	0.81	0.86	0.46	0.77	0.56	0.57	0.62	0.64	0.03	0.34	0.96	0.69	0.53	0.59
**Scanorama-HVG**	0.95	0.75	0.41	0.77	0.48	0.58	0.63	0.65	0.05	0.34	0.98	0.67	0.54	0.59
**CarDEC-HVG**	0.89	0.82	0.27	0.76	0.35	0.53	0.52	0.64	0.03	0.33	0.94	0.62	0.50	0.55
**scGESI-LVG**	0.77	0.89	0.30	0.81	0.53	0.83	0.90	0.60	0.03	0.40	0.99	0.66	0.62	0.64
**Scanorama-LVG**	0.82	0.78	0.02	0.77	0.11	0.55	0.62	0.57	0.04	0.46	0.97	0.50	0.53	0.52
**CarDEC-LVG**	0.43	0.74	0.05	0.76	0.19	0.54	0.54	0.56	0.04	0.48	0.96	0.44	0.52	0.49

Fig.S49 Performance of scGESI, Scanorama and CarDEC for whole genome denoising analysis on the HSC data. a, Visualization based on the denoised gene expression data for the entire gene set, HVGs and LVGs. UMAP plots are colored by cell identity annotations (top) and batch labels (bottom). b, Integration performance metrics based on denoised gene expression data for the entire gene set, HVGs and LVGs.

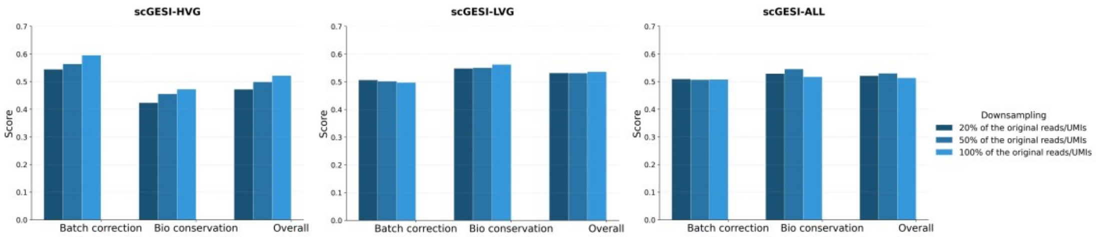

Fig. S50 Integration performance of scGESI on the HSC data after downsampling with different levels, where the sequencing depth is changed from 100% (full data) to 50% and 20% of the original counts. Integration performance metrics calculated based on HVGs (left), LVGs (middle) and the entire gene set (right).

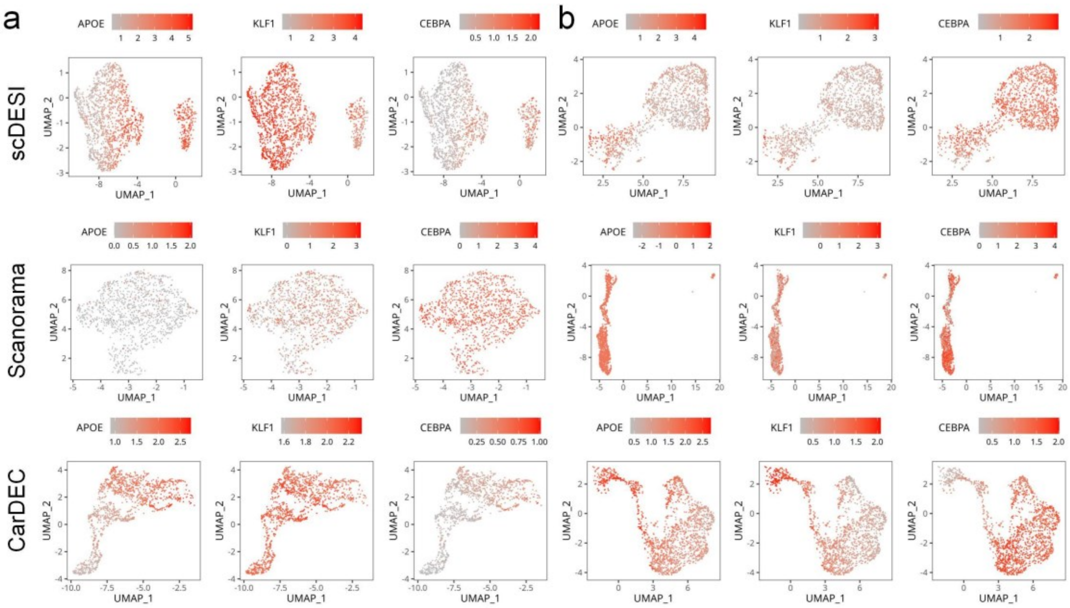

Fig.S51 Visualization based on denoised data obtained by scGESI, Scanorama and CarDEC for cells in the CMP-MEP (a) and CMP-GMP (b) trajectory. UMAP plots are colored by denoised expression of marker genes KLF1, APOE and CEBPA.

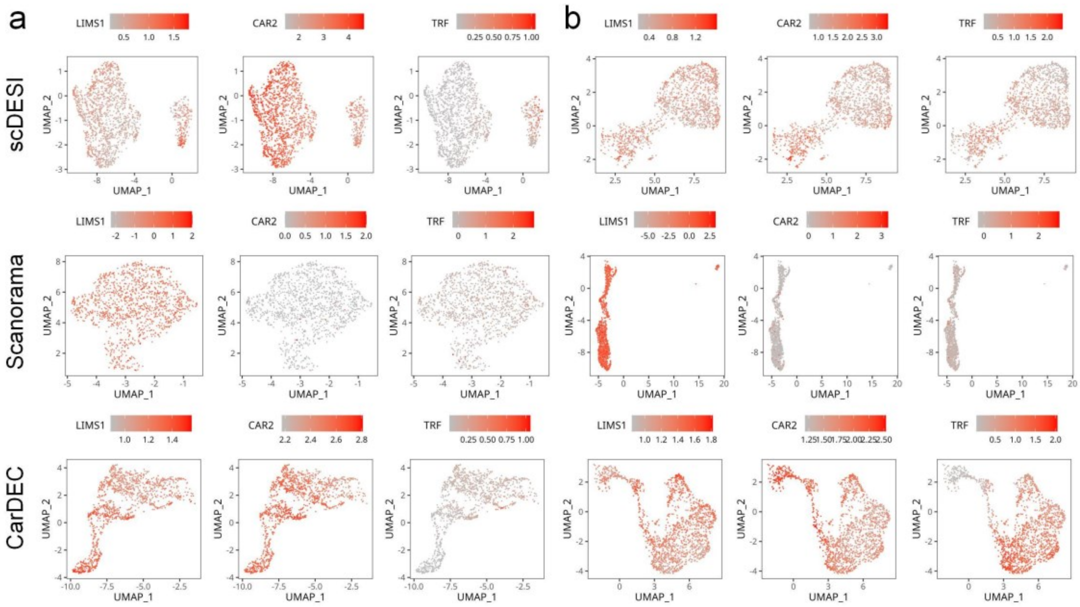

Fig.S52 The distributions of LIMS1, CAR2 and TRF in the LVG set against pseudotime from CMP to MEP (a) and GMP (b) based on the denoised gene expression data.

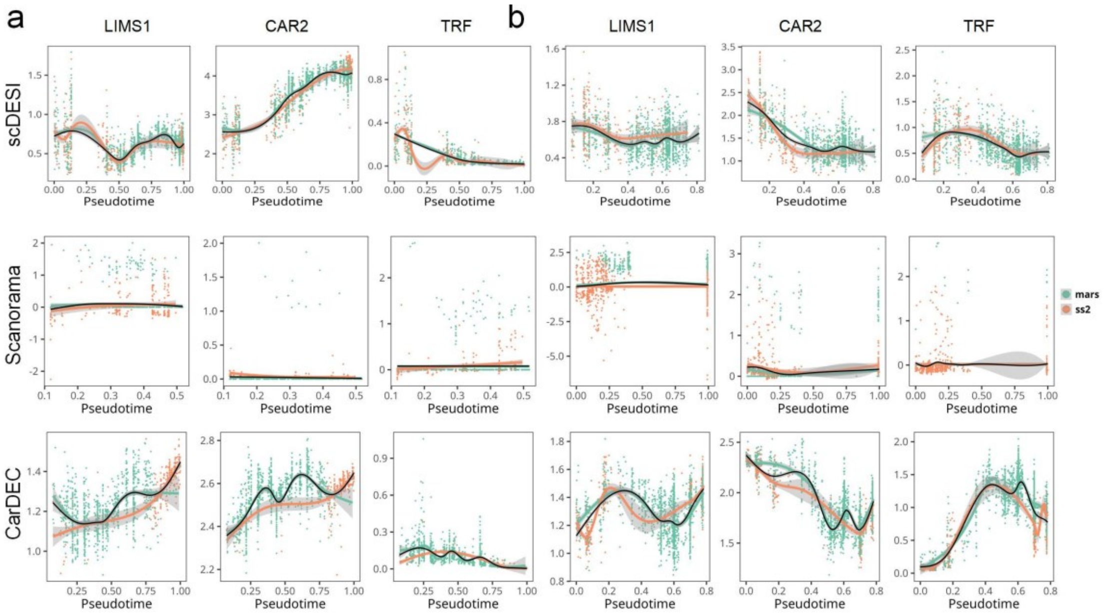

Fig.S53 Visualization based on denoised data obtained by scGESI, Scanorama and CarDEC for cells in the CMP-MEP (a) and CMP-GMP (b) trajectory. UMAP plots are colored by denoised expression of LIMS1, CAR2 and TRF.

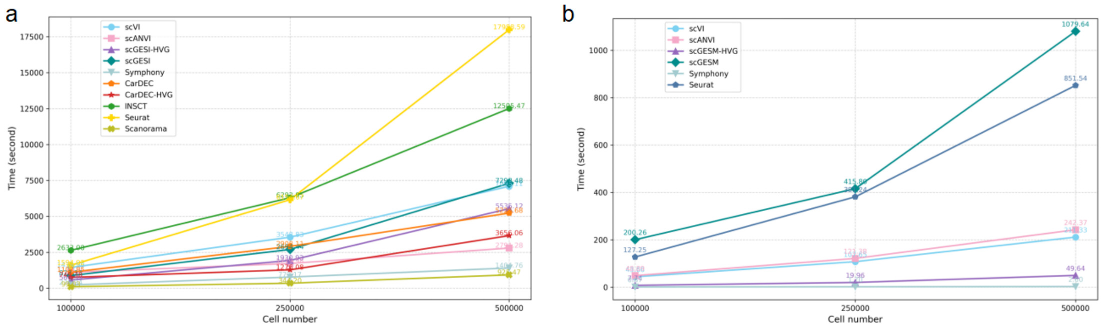

Fig.S54 Training speed of build a mapping for query data. (a: integration time; b: mapping time)

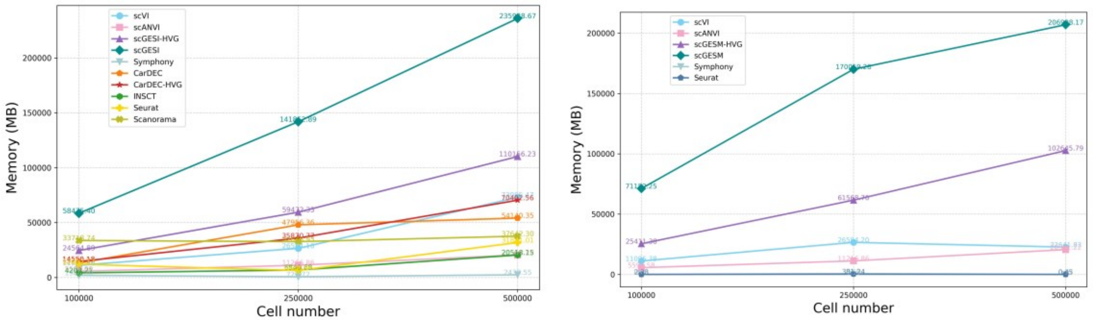

Fig. S55 Memory usage in integration (left) and mapping (right).

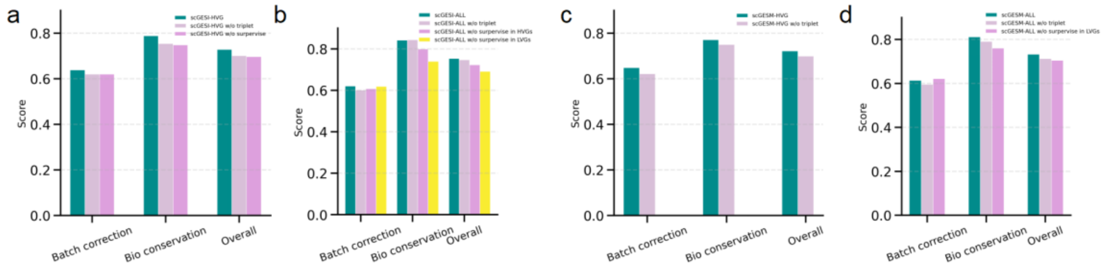

Fig.S56 Ablation study of the scGES model. a, The quantitative benchmark evaluation scores of the scGESI-HVG model and two ablated versions in the pbmc dataset. b, The quantitative benchmark evaluation scores of the scGESI model and three ablated versions in the pbmc dataset. c, The quantitative benchmark evaluation scores of the scGESM-HVG model and one ablated versions in the pbmc dataset. d, The quantitative benchmark evaluation scores of the scGESM model and two ablated versions in the pbmc dataset.

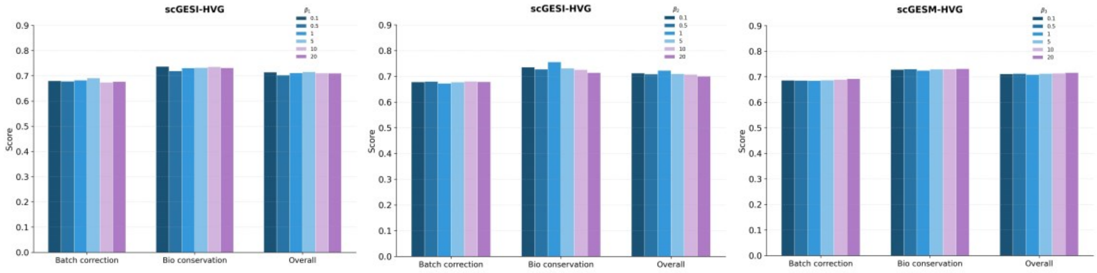

Fig. S57 Integration and mapping performance based on HVGs for the pancreas data with different β_1 (left), β_2 (middle) and β_3 (right) settings.

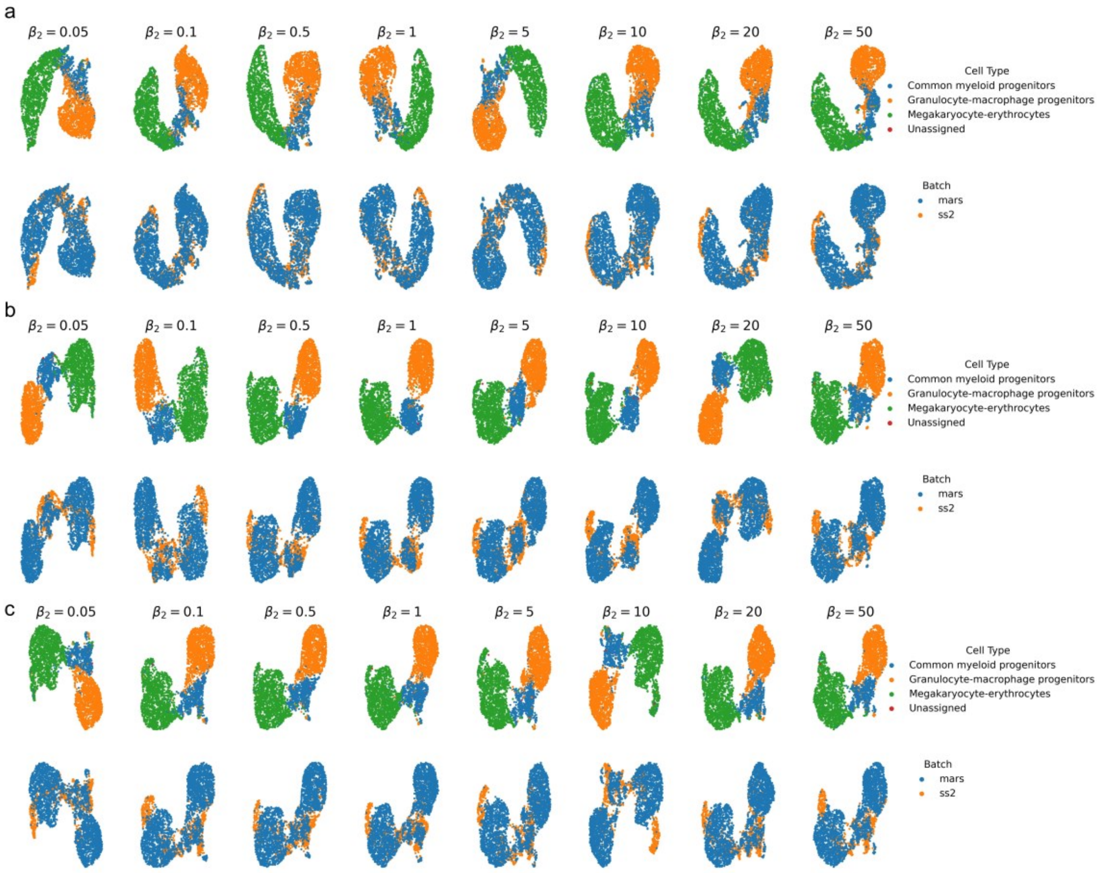

Fig. S58 Integration performance of scGESI on the HSC data with different β_2 settings. Visualizations of the integrated data based on HVGs (a), LVGs (b) and the entire gene set (c). UMAP plots are colored by cell identity annotations (top) and batch labels (bottom).

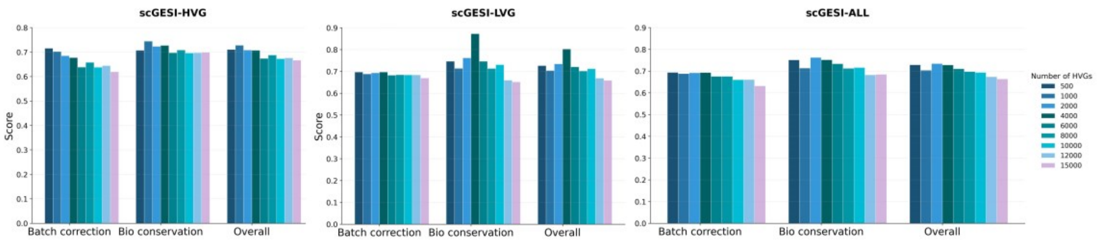

Fig. S59 Integration performance of scGESI on the pancreas data with different numbers of selected HVGs. Integration performance metrics calculated based on HVGs (left), LVGs (middle) and the entire gene set (right).

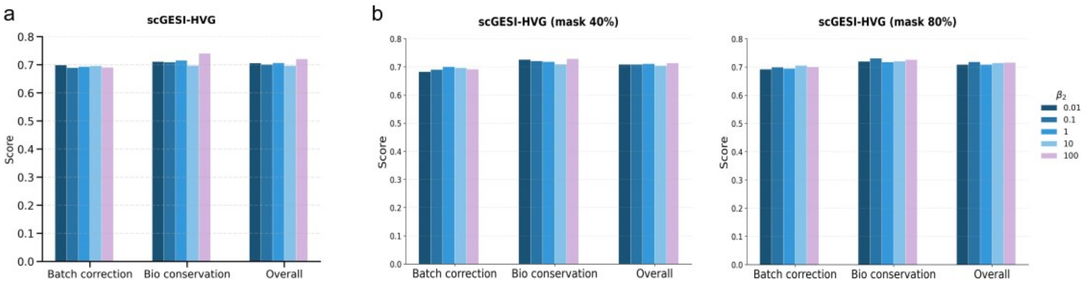

Fig. S60 Integration performance of scGESI on the pancreas data with different β_2 settings. Integration performance metrics calculated based on HVGs (left), LVGs (middle) and the entire gene set (right).

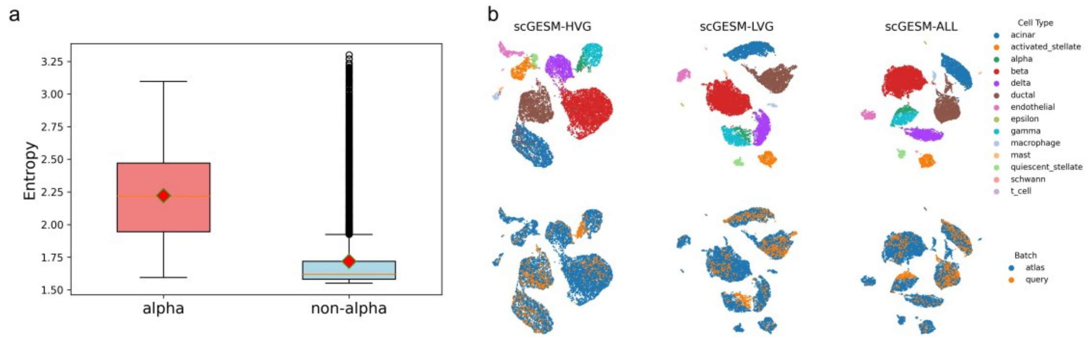

Fig. S61 Mapping performance of scGESM on the pancreas data with unseen cell state (alpha) in reference data. a, Comparison of prediction entropy in HVG branch between alpha cells and non-alpha cells. b, Visualizations of mapping the celseq batch onto the reference constructed using other batches in human pancreas data. UMAP plots are colored by cell identity annotation (top) and batch labels (bottom).

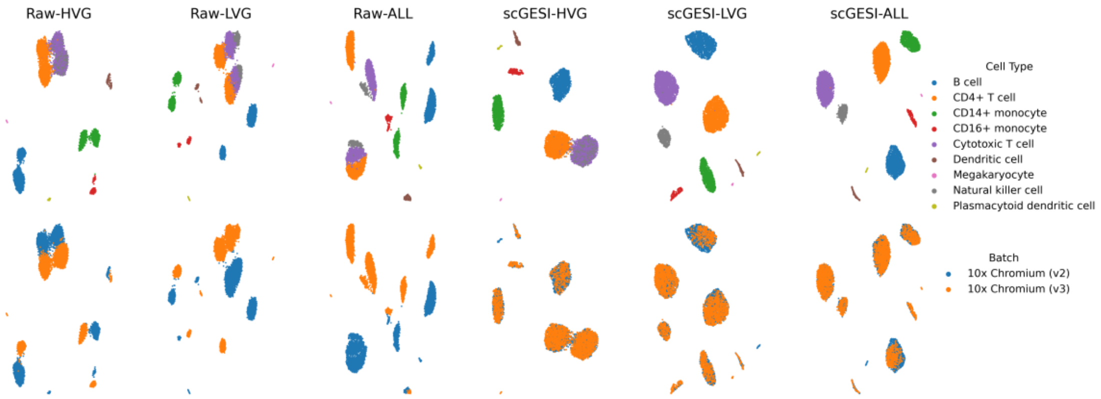

Fig. S62 Performance of scGESI in integrating on synthetic data with LVG-only biology. Visualizations based on the raw and the integrated data using scGESI for HVGs, LVGs and the entire gene set. UMAP plots are colored by cell identity annotations (top) and batch labels (bottom).

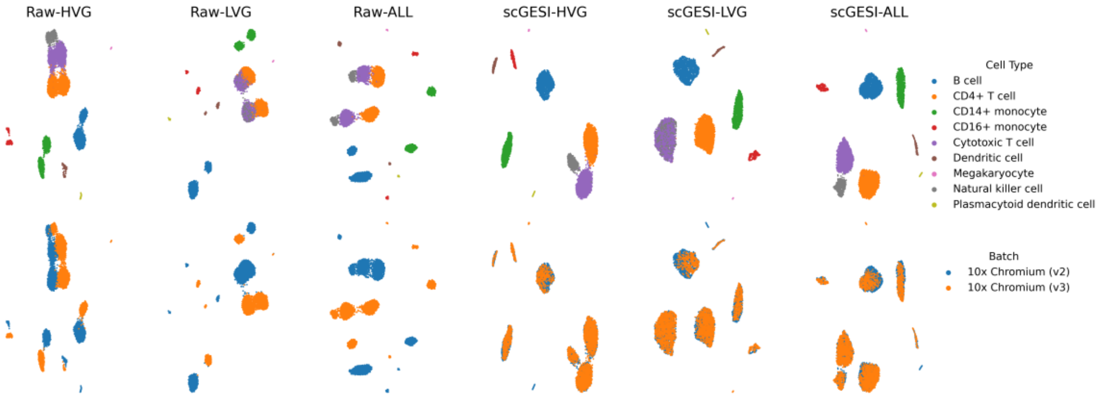

Fig. S63 Performance of scGESI in integrating on synthetic data with HVG-only biology. Visualizations based on the raw and the integrated data using scGESI for HVGs, LVGs and the entire gene set. UMAP plots are colored by cell identity annotations (top) and batch labels (bottom).

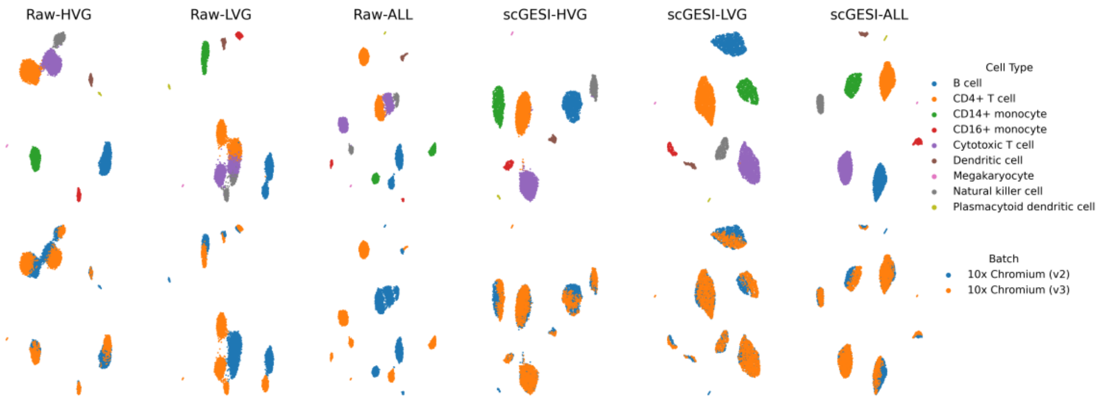

Fig. S64 Performance of scGESI in integrating on synthetic data with shared batch effects of HVGs and LVGs. Visualizations based on the raw and the integrated data using scGESI for HVGs, LVGs and the entire gene set. UMAP plots are colored by cell identity annotations (top) and batch labels (bottom).

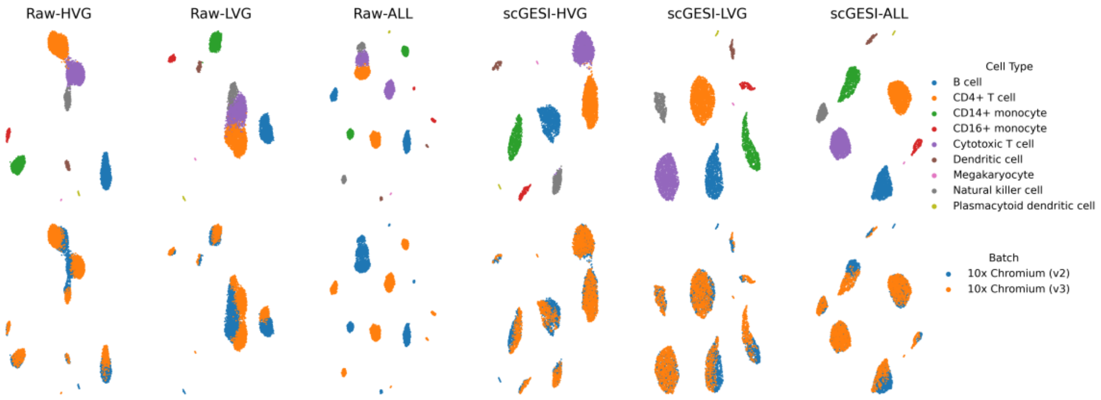

Fig. S65 Performance of scGESI in integrating on synthetic data with independent batch effects of HVGs and LVGs. Visualizations based on the raw and the integrated data using scGESI for HVGs, LVGs and the entire gene set. UMAP plots are colored by cell identity annotations (top) and batch labels (bottom).

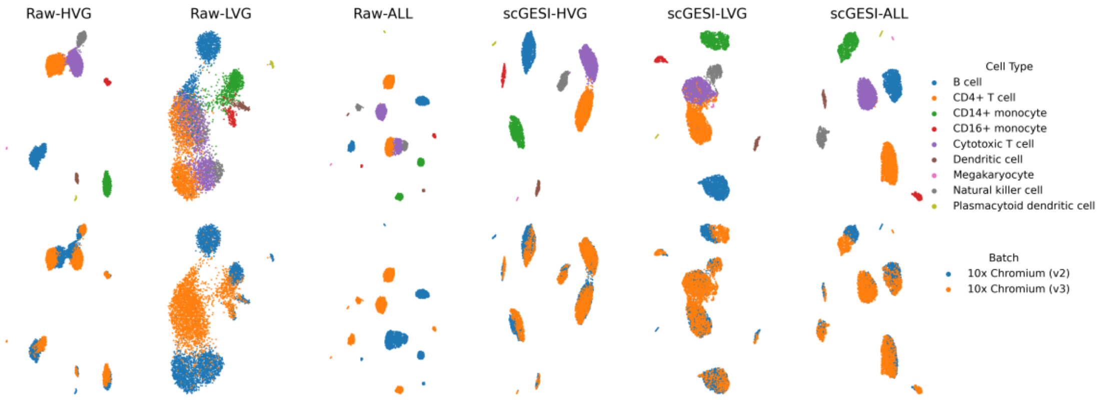

Fig. S66 Performance of scGESI in integrating on synthetic data with antagonistic batch effects of HVGs and LVGs. Visualizations based on the raw and the integrated data using scGESI for HVGs, LVGs and the entire gene set. UMAP plots are colored by cell identity annotations (top) and batch labels (bottom).

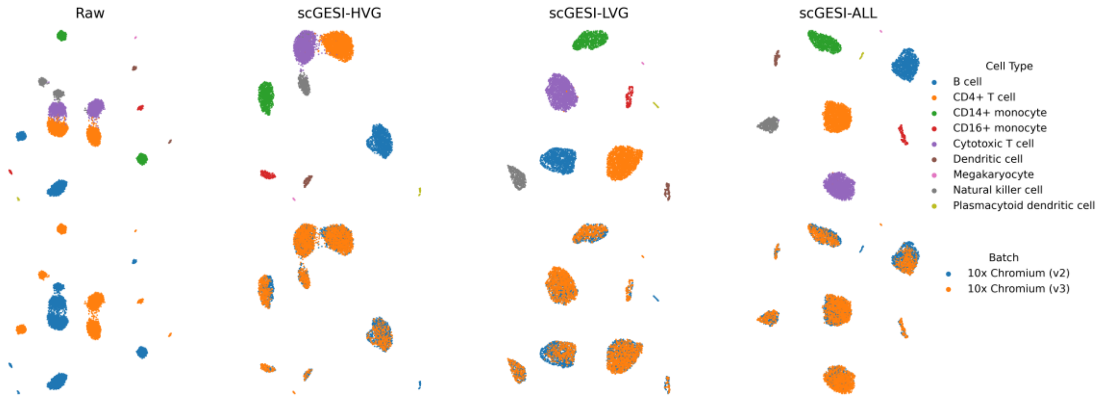

Fig. S67 Performance of scGESI in integrating on synthetic data with batch-specific dropout setting. Visualizations based on the raw and the integrated data using scGESI for HVGs, LVGs and the entire gene set. UMAP plots are colored by cell identity annotations (top) and batch labels (bottom).

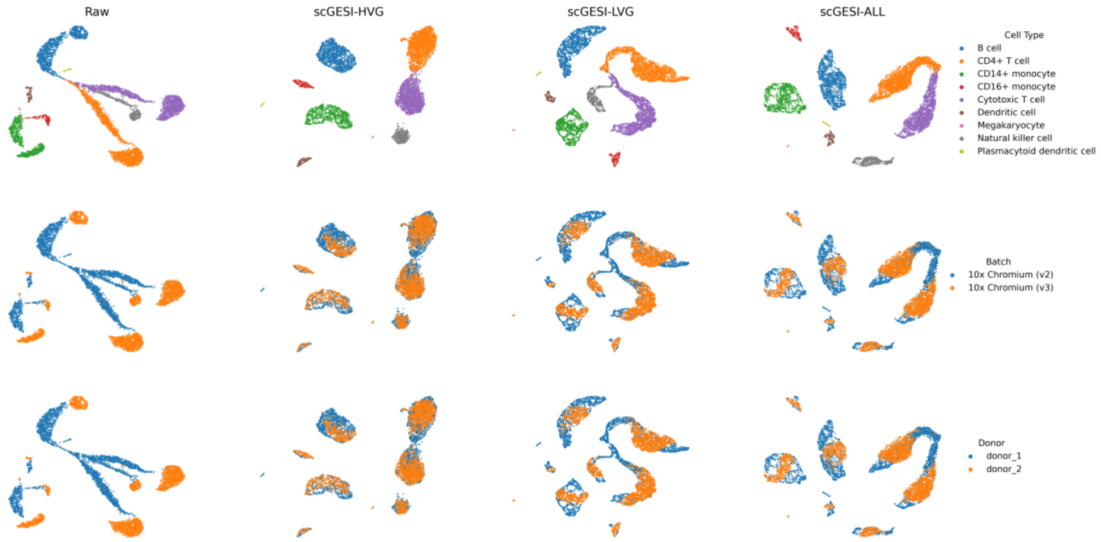

Fig. S68 Performance of scGESI in integrating on synthetic data with complete confounding. Visualizations based on the raw and the integrated data using scGESI for HVGs, LVGs and the entire gene set. UMAP plots are colored by cell identity annotations (top), batch labels (Middle) and donor labels (bottom).

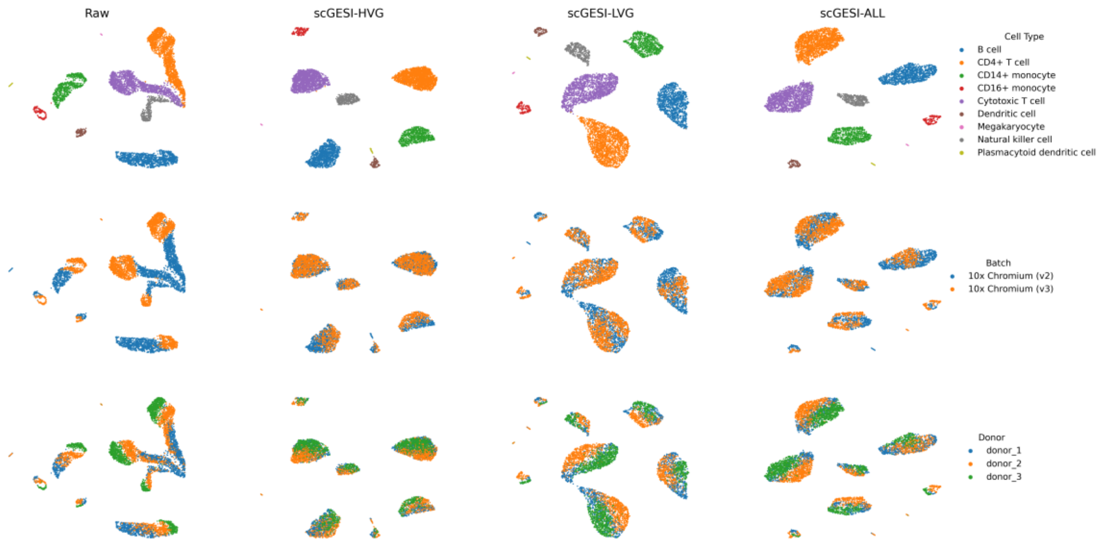

Fig. S69 Performance of scGESI in integrating on synthetic data with partial Confounding. Visualizations based on the raw and the integrated data using scGESI for HVGs, LVGs and the entire gene set. UMAP plots are colored by cell identity annotations (top), batch labels (Middle) and donor labels (bottom).

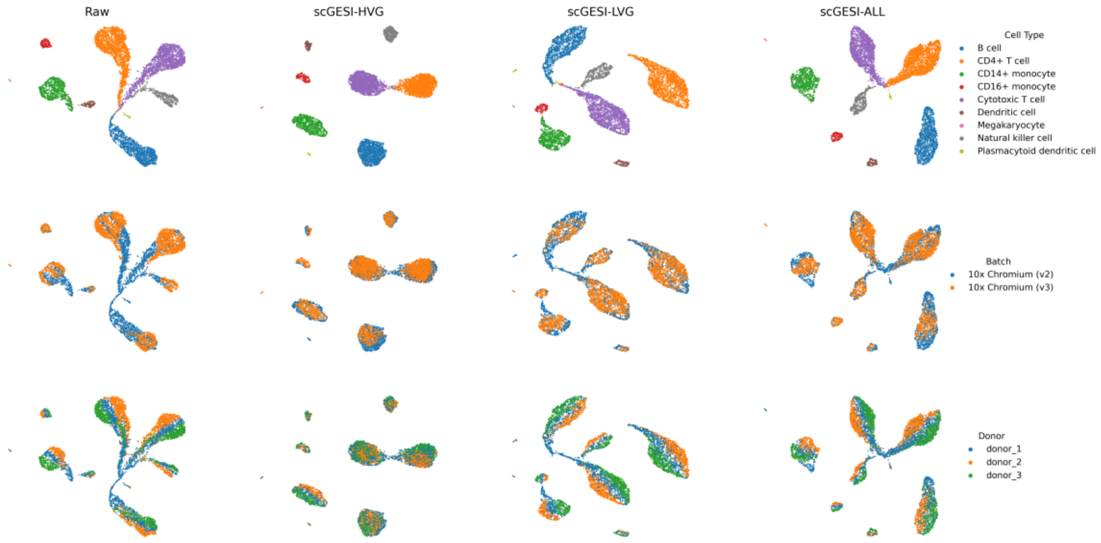

Fig. S70 Performance of scGESI in integrating on synthetic data with fully balanced. Visualizations based on the raw and the integrated data using scGESI for HVGs, LVGs and the entire gene set. UMAP plots are colored by cell identity annotations (top), batch labels (Middle) and donor labels (bottom).

Reference

[1] Balntas, V., Riba, E., Ponsa, D., & Mikolajczyk, K. (2016, September). Learning local feature descriptors with triplets and shallow convolutional neural networks. In Bmvc (Vol. 1, No. 2, p. 3).

[2] Schroff, F., Kalenichenko, D., & Philbin, J. (2015). Facenet: A unified embedding for face recognition and clustering. In Proceedings of the IEEE conference on computer vision and pattern recognition (pp. 815-823).

[3] Wang, J., Song, Y., Leung, T., Rosenberg, C., Wang, J., Philbin, J., ... & Wu, Y. (2014). Learning fine-grained image similarity with deep ranking. In Proceedings of the IEEE conference on computer vision and pattern recognition (pp. 1386-1393).
[4] Szubert, B., Cole, J. E., Monaco, C., & Drozdov, I. (2019). Structure-preserving visualisation of high dimensional single-cell datasets. Scientific reports, 9(1), 8914.
[5] Chen, W., Li, Y., Easton, J., Finkelstein, D., Wu, G., & Chen, X. (2018). UMI-count modeling and differential expression analysis for single-cell RNA sequencing. Genome biology, 19, 1-17.
[6] Wang, J., Huang, M., Torre, E., Dueck, H., Shaffer, S., Murray, J., ... & Zhang, N. R. (2018). Gene expression distribution deconvolution in single-cell RNA sequencing. Proceedings of the National Academy of Sciences, 115(28), E6437-E6446.
[7] Svensson, V. (2020). Droplet scRNA-seq is not zero-inflated. Nature Biotechnology, 38(2), 147-150.
[8] Luecken, M. D., Büttner, M., Chaichoompu, K., Danese, A., Interlandi, M., Müller, M. F., ... & Theis, F. J. (2022). Benchmarking atlas-level data integration in single-cell genomics. Nature methods, 19(1), 41-50.
[9] Lakkis, J., Wang, D., Zhang, Y., Hu, G., Wang, K., Pan, H., ... & Li, M. (2021). A joint deep learning model enables simultaneous batch effect correction, denoising, and clustering in single-cell transcriptomics. Genome research, 31(10), 1753-1766.
[10] Korsunsky, I., Millard, N., Fan, J., Slowikowski, K., Zhang, F., Wei, K., ... & Raychaudhuri, S. (2019). Fast, sensitive and accurate integration of single-cell data with Harmony. Nature methods, 16(12), 1289-1296.
[11] Xu, C., Lopez, R., Mehlman, E., Regier, J., Jordan, M. I., & Yosef, N. (2021). Probabilistic harmonization and annotation of single-cell transcriptomics data with deep generative models. Molecular systems biology, 17(1), e9620.
[12] Lopez, R., Regier, J., Jordan, M. I., & Yosef, N. (2018). Information constraints on auto-encoding variational bayes. Advances in neural information processing systems, 31.
[13] Hie, B. L., Kim, S., Rando, T. A., Bryson, B., & Berger, B. (2024). Scanorama: integrating large and diverse single-cell transcriptomic datasets. Nature protocols, 19(8), 2283-2297.
[14] Peng, M., Li, Y., Wamsley, B., Wei, Y., & Roeder, K. (2021). Integration and transfer learning of single-cell transcriptomes via cFIT. Proceedings of the National Academy of Sciences, 118(10), e2024383118.
[15] Stuart, T., Butler, A., Hoffman, P., Hafemeister, C., Papalexi, E., Mauck, W. M., ... & Satija, R. (2019). Comprehensive integration of single-cell data. cell, 177(7), 1888-1902.
[16] Simon, L. M., Wang, Y. Y., & Zhao, Z. (2021). Integration of millions of transcriptomes using batch-aware triplet neural networks. Nature Machine Intelligence, 3(8), 705-715.
[17] Kang, J. B., Nathan, A., Weinand, K., Zhang, F., Millard, N., Rumker, L., ... & Raychaudhuri, S. (2021). Efficient and precise single-cell reference atlas mapping with Symphony. Nature communications, 12(1), 5890.
[18] Lotfollahi, M., Naghipourfar, M., Luecken, M. D., Khajavi, M., Büttner, M., Wagenstetter, M., ... & Theis, F. J. (2022). Mapping single-cell data to reference atlases by transfer learning. Nature biotechnology, 40(1), 121-130.
[19] Büttner, M., Miao, Z., Wolf, F. A., Teichmann, S. A., & Theis, F. J. (2019). A test metric for assessing single-cell RNA-seq batch correction. Nature methods, 16(1), 43-49.
[20] Korsunsky, I., Millard, N., Fan, J., Slowikowski, K., Zhang, F., Wei, K., ... & Raychaudhuri, S. (2019). Fast, sensitive and accurate integration of single-cell data with Harmony. Nature methods, 16(12), 1289-1296.
[21] Hubert, L., & Arabie, P. (1985). Comparing partitions. Journal of classification, 2, 193-218.
[22] Pedregosa, F., Varoquaux, G., Gramfort, A., Michel, V., Thirion, B., Grisel, O., ... & Duchesnay, É. (2011). Scikit-learn: Machine learning in Python. the Journal of machine Learning research, 12, 2825-2830.
[23] Song, D., Wang, Q., Yan, G., Liu, T., Sun, T., & Li, J. J. (2024). scDesign3 generates realistic in silico data for multimodal single-cell and spatial omics. Nature Biotechnology, 42(2), 247-252.
